# Methanol-Enhanced Low-Cell-Voltage Hydrogen Generation at Industrial-Grade Current Density by Triadic Active Sites of Pt_1_–Pd*_n_*–(Ni,Co)(OH)_*x*_

**DOI:** 10.1021/jacs.4c12665

**Published:** 2025-01-13

**Authors:** An Pei, Ruikuan Xie, Lihua Zhu, Fengshun Wu, Zinan Huang, Yongyu Pang, Yu-Chung Chang, Guoliang Chai, Chih-Wen Pao, Qingsheng Gao, Congxiao Shang, Guang Li, Jinyu Ye, Huaze Zhu, Zhiqing Yang, Zhengxiao Guo

**Affiliations:** † Jiangxi Province Key Laboratory of Functional Crystalline Materials Chemistry, College of Chemistry and Chemical Engineering, Faculty of Materials Metallurgy and Chemistry, Jiangxi University of Science and Technology, Ganzhou 341000, Jiang Xi, China; ‡ Department of Chemistry, The University of Hong Kong, Hong Kong Island 000000, Hong Kong SAR, China; § State Key Laboratory of Structural Chemistry, Fujian Institute of Research on the Structure of Matter, Chinese Academy of Sciences (CAS), Fuzhou 350002, Fujian, China; ∥ State Key Laboratory for Physical Chemistry of Solid Surfaces, College of Chemistry and Chemical Engineering, Xiamen University, Xiamen 361005, China; ⊥ College of Chemistry and Materials Science and Guangdong Provincial Key Laboratory of Functional Supramolecular Coordination Materials and Applications, Jinan University, Guangzhou 510632, China; # National Synchrotron Radiation Research Center, Hsinchu 30076, Taiwan; ∇ Ji Hua Laboratory, Foshan 528200, China

## Abstract

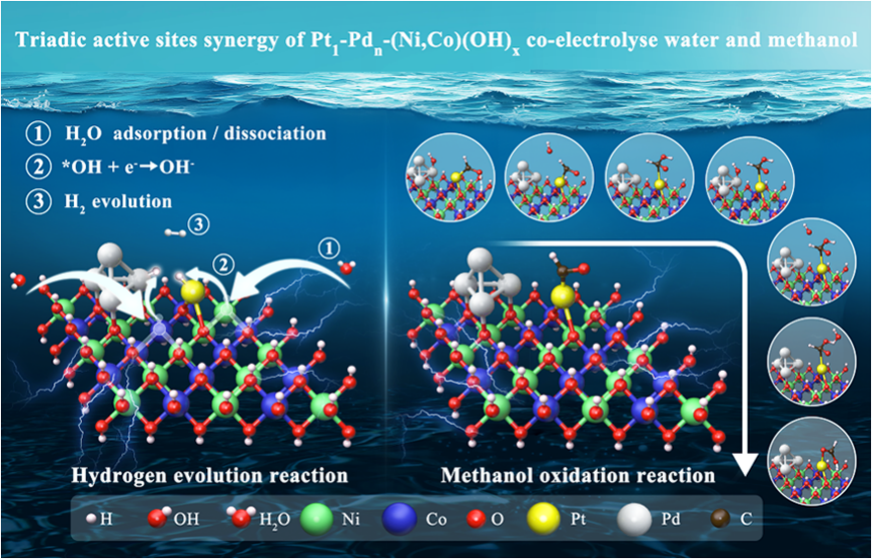

Methanol (ME) is a liquid hydrogen carrier, ideal for on-site-on-demand H_2_ generation, avoiding its costly and risky distribution issues, but this “ME-to-H_2_” electric conversion suffers from high voltage (energy consumption) and competitive oxygen evolution reaction. Herein, we demonstrate that a synergistic cofunctional Pt_1_Pd*_n_*/(Ni,Co)(OH)_*x*_ catalyst with Pt single atoms (Pt_1_) and Pd nanoclusters (Pd*_n_*) anchored on OH-vacancy(V_OH_)-rich (Ni,Co)(OH)_*x*_ nanoparticles create synergistic triadic active sites, allowing for methanol-enhanced low-voltage H_2_ generation. For MOR, OH* is preferentially adsorbed on Pd*_n_* and then interacts with the intermediates (such as *CHO or *CHOOH) adsorbed favorably on neighboring Pt_1_ with the assistance of hydrogen bonding from the surface hydrogen of (Ni,Co)(OH)_*x*_. The enhanced selectivity of the *CHOOH pathway, instead of *CO, sustains the MOR activity to a practically high current density. For HER, triadic Pt_1_, Pd*_n_*, and OH-vacancy sites on (Ni,Co)(OH)_*x*_ create an “acid–base” microenvironment to facilitate water adsorption and splitting, forming H* species on Pt_1_ and Pd*_n_*, and *OH at the vacancy, to promote efficient H_2_ evolution from the asymmetric Pt_1_ and Pd*_n_* sites via the Tafel mechanism. The triadic-site synergy opens new avenues for the design and synthesis of highly efficient and stable cofunctional catalysts for “on-site-on-demand” H_2_ production, here facilitated by liquid methanol.

## 1 Introduction

Wide spatial disparities between supply and demand hamper the wide utilization of hydrogen (H_2_) as a “net-zero or “carbon-negative” energy carrier. Methanol (ME) is a desirable liquid hydrogen carrier for energy storage and distribution, and its efficient conversion to H_2_ is of great significance as the demand for hydrogen increases at least by 8-fold for targeted net-zero emissions in coming decades.^1,2^ It can be used directly in direct methanol fuel cells (DMFCs) but with a relatively low power rating or indirectly in reformed methanol fuel cells (RMFCs) after conversion or reforming into H_2_ to enhance the power capacity. However, conventional methanol (steam) reforming for H_2_ (CH_3_OH (g) + H_2_O (g) = CO_2_ (g) + 3 H_2_ (g), Δ*H* = +49.4 kJ mol^–1^) requires high temperature (250–300 °C) and pressure (*e.g.*, 5.0 MPa), often with impurities of CO, HCOOH, and CH_3_OH (unreacted) steam that are hard to remove and harmful to the catalyst.^1−4^ Indeed, a thermal reformer can only attain a net thermal efficiency of 45%, even with high methanol conversion (>99%), along with a residual CO content of 0.8%.^1^ Moreover, further purification of hydrogen is difficult and costly; thus, it is highly desirable to develop a cost-effective reforming approach to generate high-purity H_2_ from methanol,^3,4^*e.g.*, electrochemically under ambient conditions, ideally without the release of CO_2_, or better still transforming CO_2_ into high-value-added products.^5−7^ Here, we propose a new cost-effective approach from methanol-mediated electrochemical H_2_ production under ambient conditions by “methanol and water co-electrolysis,” which is particularly suitable for distributed generation of hydrogen on-site and on-demand, addressing the end-user supply challenges of hydrogen,^5−7^ particularly for marine shipping and geographically not-so-densely populated sites.

Compared with traditional alkaline water electrolysis (AWE, H_2_O = H_2_ (g) + 1/2 O_2_ (g), Δ*H* = +285.84 kJ mol^–1^ H_2_) for H_2_ release, anodic methanol electro-oxidation reaction (MOR) can be favorably utilized to replace the sluggish oxygen evolution reaction (OER) due to its lower theoretical standard electro-oxidation potential (∼0.016 V) than H_2_O (>1.230 V).^8,9^ Moreover, the reactive oxygen species of the OER process also suffers from the propensity for membrane-crossing to the cathode, causing potential safety hazards, whereas a [MOR||HER] cell is much safer to operate without oxygen generation. However, non-noble metals require a relatively high voltage (>1.230 V) for anodic MOR, and the cell voltage is even higher (>1.600–2.800 V) for [MOR||HER] at a practically viable current density of >200 mA cm^–2^, which causes extensive energy consumption.^10−12^ Though noble Pt (Pd)-based catalysts can activate MOR at ∼0.300 V,^13−15^ low-voltage [MOR||HER] coupling has not been effectively achieved with a sufficiently high current density due to multiple issues, such as weak adsorption of reaction intermediates (CHO*, COOH*), the CO-mediated pathway, and the electro-oxidization of Pt (Pd) to PtO_2_ (PdO).^13−17^ For the HER, the Pt-based catalyst still shows sluggish kinetics for both the Volmer step (breaking of the H–OH bond) and the Heyrovsky step (too strong hydrogen adsorption).^18−25^ Such limitations call for the development of more cost-effective MOR and HER catalysts, ideally with “cofunctionality” that can directly synchronize the coupling of MOR and HER into a “methanol and water co-electrolyzer,” or fuel processing system, for distributed hydrogen generation “on demand and on-site,” efficiently at a low cell voltage.

Since both the HER and MOR are multistep reactions, the judicial design of multiple active sites can avoid the binding-energy scaling limitation of reaction intermediates and make full use of the synergistic effects to enhance reactivity and selectivity.^26−32^ Generally speaking, Pt single atoms demonstrate excellent HER activity, and the Pd clusters are more effective in molecular electro-oxidation with high resistance to CO poisoning,^33−36^ and a rational combination of the two is expected to enhance the activity and selectivity of the two different electro redox reactions. However, water splitting on bare Pt/Pd surfaces suffers from poor adsorption/activation of water molecules and inadequate H* binding for HER.^21,22^ Therefore, it is envisaged that creating a strong “acid–base separation microenvironment” at the active site should be effective in facilitating the electrochemical splitting of polar molecules such as water and methanol. Bimetallic hydroxides stand out as a suitable support to enrich surface defects and provide adjustable coordination for the catalytic species,^21−25^*e.g.*, the acidic site at the surface vacancies or hydrogen bonding at the hydroxyls, as clarified in a later section.

Here, we propose an efficient, stable, and cofunctional “triadic site” catalyst, Pt_1_Pd*_n_*/(Ni,Co)(OH)_*x*_/C (termed as “TS catalyst”), to directly couple HER and MOR, addressing the challenges of achieving high current density. The key features include (1) high surface area carbon black matrix providing accessible active sites and conductivity; (2) bimetallic (Ni,Co)(OH)_*x*_ nanoparticles for tuning surface electronic and chemical properties, enhancing OH defects generation; and (3) cooperative triactive sites of Pt single atoms (Pt_1_), Pd atomic nanoclusters (Pd*_n_*), and surface vacancy or hydrogen sites on the hydroxide support for MOR and HER coactivation. *In situ* characterizations and density functional theory (DFT) calculations revealed the specific synergistic mechanisms of such triadic sites for both MOR and HER. For MOR, the catalyst improves the adsorption of reaction intermediates, favoring *CHOOH over *CO pathway and preventing the electro-oxidation of Pt_1_ and Pd*_n_* to PtO_2_ and PdO due to local electronic redistribution, creating a fitting “acid–base” microenvironment among Pt_1_, Pd*_n_*, and the surface hydrogen sites of (Ni,Co)(OH)_*x*_. For HER, the synergy of Pt_1_, Pd*_n_*, and OH vacancy sites facilitates water activation, leading to H_2_ formation via the Tafel step from asymmetrically adsorbed *H on Pt_1_ and Pd*_n_* (*H_Pt_ + *H_Pd_ → H_2_). Simultaneous desorption of *OH regenerates the vacancy sites for the next HER cycle. Such synergistic coordinations of transient species make the TS catalyst an excellent candidate for HER in alkaline solutions. Consequently, the TS catalyst achieves a high current density of 700 mA cm^–2^ for efficient pure hydrogen production under an ultralow cell voltage.

## 2 Results and Discussion

### 2.1 Synthesis and Characterization of the Catalysts

**Figure 1:**
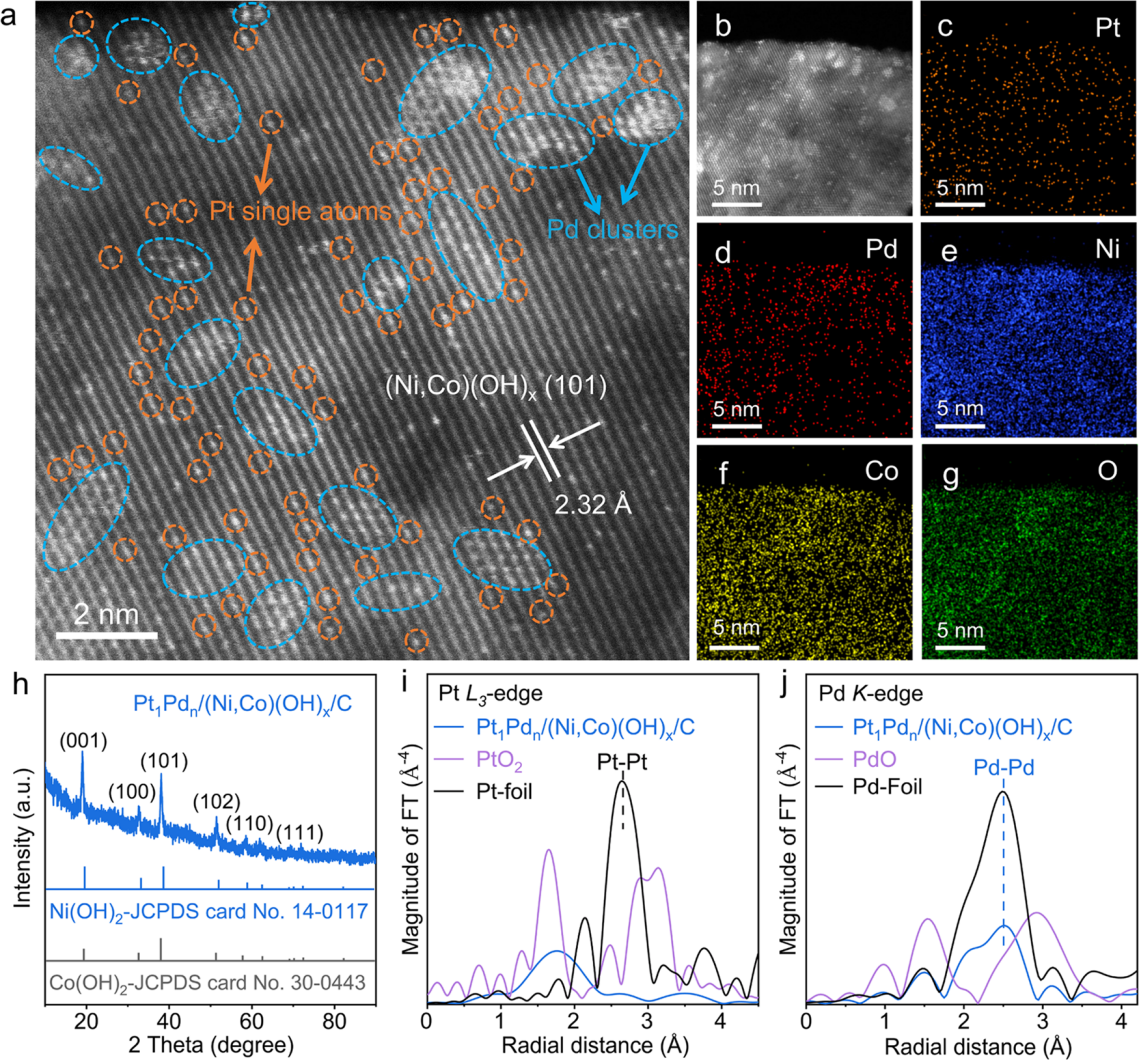
Preparation and structural analysis of the TS catalyst. (a) AC-STEM images of the TS catalyst. Scale bar: 2 nm. (b) Representative AC-STEM image and corresponding (c) Pt, (d) Pd, (e) Ni, (f) Co, and (g) O elemental maps. Scale bars: (b–g) 5 nm. (h) XRD patterns of the TS catalyst. Fourier transformed EXAFS spectra for the TS catalyst at (i) Pt *L*_3_-edge and (j) Pd *K*-edge.

The NiCo/(Ni,Co)(OH)_*x*_/C was synthesized via chemical reduction, and Pt_a_Pd_b_/(Ni,Co)(OH)_*x*_/C was obtained by a galvanic replacement synthesis method (Figure S1, method). Aberration-corrected scanning transmission electron microscopy (AC-STEM) images confirm the formation of Pt single atoms and Pd atomic clusters on (Ni,Co)(OH)_*x*_/C (Figures 1a–g and S2). The X-ray diffraction (XRD) results are consistent with the AC-STEM observations (Figures 1h and S3). Electron paramagnetic resonance (EPR) spectra for the Pt_1_Pd*_n_*/(Ni,Co)(OH)_*x*_/C and NiCo/(Ni,Co)(OH)_*x*_/C samples confirm that the OH defects are abundant, which are further enriched by the Pt_1_ and Pd*_n_* species (Figure S4). The X-ray absorption near-edge structure (XANES) spectra of the TS catalyst show a zero-energy shift from Pt(0) to Pt^4+^ (Figure S5a). The Pt *L*_3_-edge extended X-ray absorption fine structure (EXAFS) spectra of the TS catalyst show a Pt–O contribution peak at 2.1 Å, but no contribution peak of the Pt–Pt bond (2.7 Å) is observed (Figure 1i), implying that the Pt single atoms are trapped and stabilized on the (Ni,Co)(OH)_*x*_ surface. The best-fit EXAFS parameters of these catalysts and the reference compound are given in Table S1, and the Fourier transformed EXAFS data and the best-fit results are also shown in Figures S6–S9. The coordination number (CN) of Pt–O in the TS catalyst, Pt_1_Pd*_n_*/Ni(OH)_*x*_/C, and Pt_1_Pd*_n_*/Co(OH)_*x*_/C are 2.7, 4.1, and 3.2, with a distance of 2.09, 2.08, and 2.08 Å, respectively, for these three catalysts. There is no other contribution peak, indicating Pt with single atoms. In Figure S5b, the XANES spectra demonstrate that the Pd-related species are mainly in the form of Pd(0). Pt *L*_3_- and Pd *K*-edge EXAFS spectra of the PtPdNiCo/C (without the hydroxide) show that the Pt atoms mainly coordinate with Pt, O while Pd coordinates with Pd, Co(Ni) atoms in PtPdNiCo/C. Ni *K*-edge XANES spectrum of the TS catalyst shows Ni(OH)_2_ as the feature but slightly reduced to the metallic state (Figure S5c). Metallic Pd–Pd coordination of the TS catalyst (or Pt_1_Pd*_n_*/Ni(OH)_*x*_/C and Pt_1_Pd*_n_*/Co(OH)_*x*_/C) shows a prominent peak at 2.7 Å. A peak at 2.6 Å presents the Pd–Co or Pd–Ni coordination of the TS catalyst (or Pt_1_Pd*_n_*/Ni(OH)_*x*_/C, Pt_1_Pd*_n_*/Co(OH)_*x*_/C), implying the Pd clusters on the (Ni,Co)(OH)_*x*_ (or Co(OH)_*x*_, Ni(OH)_*x*_) surface (Figure 1j). The contribution at a distance of *ca.* 2.0 Å for the TS catalyst (or Pt_1_Pd*_n_*/Ni(OH)_*x*_/C, Pt_1_Pd*_n_*/Co(OH)_*x*_/C) is attributed to the Pd–O bond (Figure S8). The Co *K*-edge XANES spectra indicate the Co^2+^ state (Figure S5d). The TS catalyst shows Ni–O (2.1 Å) and Ni–Ni (2.5 Å) bonds (Figure S5e and Table S1). The two bonding distances at 2.2 and 2.4 Å correspond to the Co–O and Co–Co bonds, respectively (Figure S5f and Table S1). The binding energies of Pt 4f of Pt_1_Pd*_n_*/(Ni,Co)(OH)_*x*_/C demonstrate a negative shift compared with Pt_1_Pd*_n_*/Ni(OH)_*x*_/C, confirming the successful combination of Ni(OH)_*x*_ and Co(OH)_*x*_ to form (Ni,Co)(OH)_*x*_, and further increasing Pt electron density (Figures S10 and S11).^37^

Pd 3d X-ray photoelectron spectroscopy (XPS) spectrum for the TS catalyst is positively shifted relative to Pd_n_/(Ni,Co)(OH)_*x*_/C, implying that Pd donates some electrons to Pt. High-sensitivity low-energy ion scattering spectra (HS-LEIS) for the catalysts confirm that the concentrations of Pt, Pd, Co, and Ni on the surface agree with other results (Figure S12).

### 2.2 Electrocatalytic Performance of MOR

**Figure 2:**
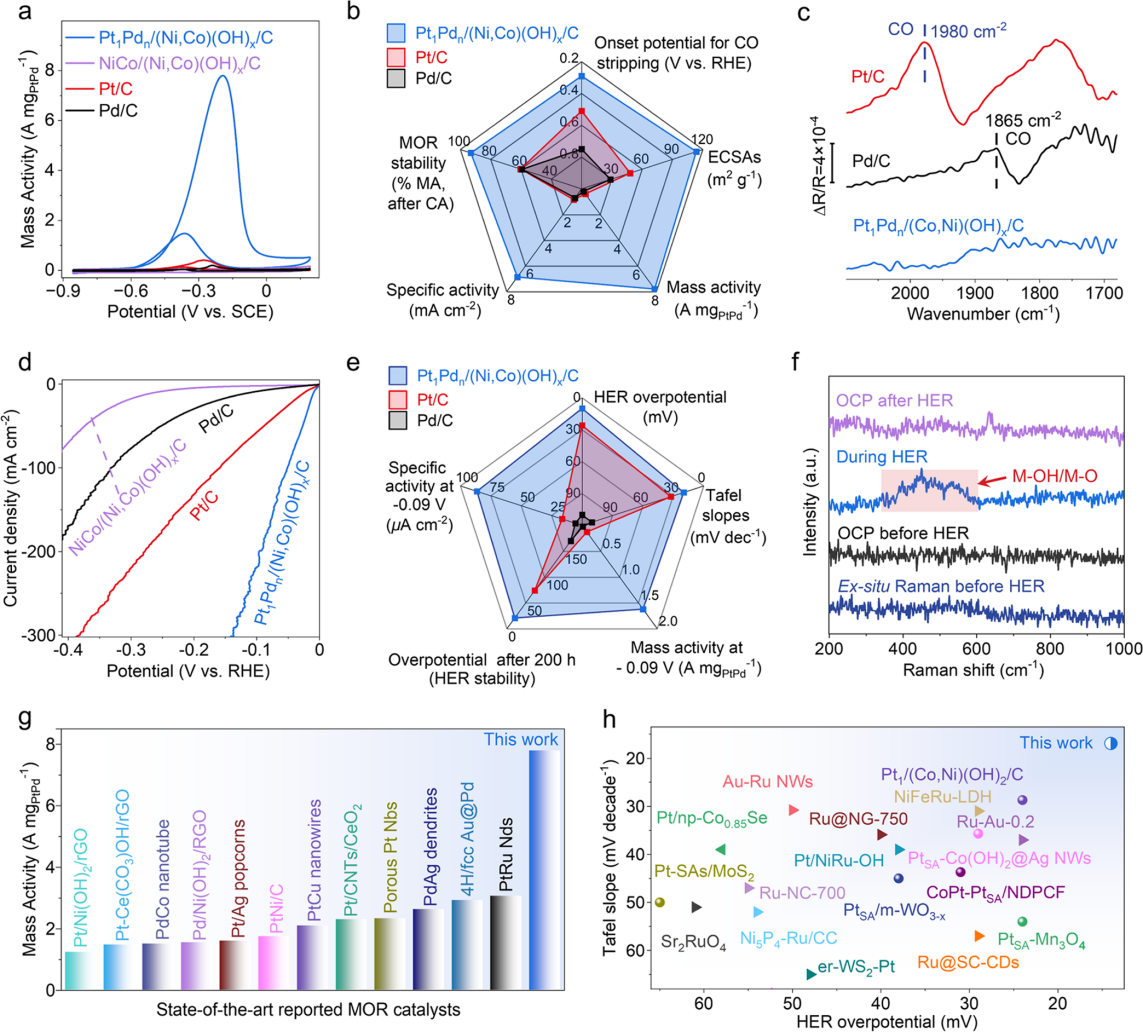
Electrocatalytic performance of the TS catalyst toward the MOR and HER. (a) PtPd content-normalized cyclic voltammograms for the MOR in 1.0 M KOH + 1.0 M CH_3_OH solution. (b) Comparison of the catalytic MOR performances of the TS catalyst, Pt/C catalyst, and Pd/C catalysts. (c) Electrochemical *in situ* FTIR spectra of the catalysts during the MOR. (d) HER polarization curves of as-obtained catalysts in 1.0 M KOH solution. Scanning rate, 5 mV s^–1^. (e) Comparison of the catalytic HER performances of the TS catalyst, Pt/C catalyst, and Pd/C catalysts. (f) *Ex situ* Raman spectra before HER, *Operando* Raman spectra at OCP before HER, during HER at −1.3 V (*vs* Hg/HgO), and open-circuit potential (OCP) after HER for the TS catalyst. Comparison of the catalytic (g) MOR and (h) HER performances of the TS catalyst and state-of-the-art catalysts.

The TS catalyst afforded significantly enhanced mass activity (MA) compared with Pt_a_Pd_b_/(Ni,Co)(OH)_*x*_/C, Pt_1_/(Ni,Co)(OH)_*x*_/C, and Pd*_n_*/(Ni,Co)(OH)_*x*_/C (Figures 2a and S13). Moreover, the MOR efficiency of the TS catalyst was also the highest among Pt_1_Pd*_n_*/Ni(OH)_*x*_/C, Pt_1_Pd*_n_*/Co(OH)_*x*_/C, PtPdNiCo/C, and PtPd/C, indicating that (Ni,Co)(OH)_*x*_ was crucial for efficient MOR (Figure S13). The MA of the TS catalyst was 7.796 A mg_PtPd_^–1^, which was 19.34 times over Pt/C and 38.98 times over Pd/C, respectively (Figures 2b and S14, and Table S2). Besides, the TS catalyst displayed a MOR MA larger than those of other state-of-the-art electrocatalysts reported to date (Table S3).

The catalytic performance of PtPdNiCo/C (*i.e.*, replacing the hydroxide support with the metallic counterpart) dropped significantly to 0.281 A mg_PtPd_^–1^, indicating further the importance of the hydroxide for effective MOR. Besides, the TS catalyst shows the lowest onset potential (0.290 V) for CO-stripping, demonstrating its enhanced CO electro-oxidation activity than PtPdNiCo/C (0.480 V), Pt/C (0.510 V), and Pd/C (0.570 V) (Figures 2b and S15). Furthermore, the electrochemical active surface areas (ECSA) of TS catalyst (113.6 m^2^ g_PtPd_^–1^), Pt/C (48.1 m^2^ g_Pt_^–1^), and Pd/C (28.6 m^2^ g_Pd_^–1^) were determined, and the specific activity (ECSA normalized) of the TS catalyst (6.86 mA cm^–2^) was 8.17 times over Pt/C (0.84 mA cm^–2^) and 9.80 times over Pd/C (0.70 mA cm^–2^) (Figure S14). The optimized specific activity of the TS catalyst indicated the enhanced intrinsic activity of the Pt and Pd species by their effective integration with (Ni,Co)(OH)_*x*_. The TS catalyst also exhibited improved MOR stability (93.1% reserved after 4000 s chronoamperometry) compared to Pt/C (60.3%) and Pd/C (59.5%) (Figure S16).

In addition, the initial TS catalyst reactivated after MOR confirmed that there was no significant structural or chemical change of the catalyst, confirming the suppression of the electro-oxidation of Pt (Pd) species to PtO_2_ (PdO) because of the strong interaction of the Pt (Pd) species with the hydroxide substrate (Figures S17–S19). Overall, the TS catalyst shows all-around improvement over Pt/C and Pd/C for MOR (Figure 2b) and is much superior to its state-of-the-art counterparts (Figure 2g and Table S3). The electrochemical *in situ* Fourier transform infrared (FTIR) spectra of the catalysts show that the oxidation of methanol molecule prefers the HCOO^–^ pathway,^32,33^ rather than the CO pathway, to generate CO_2_ in terms of the reaction kinetics on the TS catalyst (Figures 2c and S20). The linearly bonded CO_ad_ species (1980 cm^–1^) on Pt/C and bridge-bound CO_ad_ species on Pd/C were probed, while the CO species were not detected on the TS catalyst, indicating that a CO-free pathway was realized here.^33−35^ The potential dependence of relative concentrations (CR) of HCOO^–^, CO_3_^2–^, and CO_2_ from the MOR on the TS catalyst, Pt/C catalyst, and Pd/C catalyst were obtained (Figures S21 and S22).^35^ Pt_1_Pd*_n_*/(Ni,Co)(OH)_*x*_/C requires the lowest voltage to produce those species, which further confirms its enhanced MOR activity.

### 2.3 Electrocatalytic Performance of HER

The PtPd/(Ni,Co)(OH)_*x*_/C with different Pt/Pd atomic ratios show superior activity to Pt/C (Figures S23 and S24) and other reported electrocatalysts (Table S4). Particularly, the TS catalyst yielded an extremely high HER activity that required an ultralow overpotential of 10 and 51 mV to obtain 10 and 100 mA cm^–2^, respectively, compared to those of 26 and 158 mV on Pt/C (Figures 2d, S23, and S24 and Table S4). The Tafel slope of the TS catalyst was merely 19.8 mV dec^–1^ (Figure S24), indicating that the overall HER over this catalyst followed the Volmer–Tafel mechanism while Pt/C (32.5 mV dec^–1^) and Pd/C (110.4 mV dec^–1^) followed the Volmer–Heyrovsky mechanism.^21−24^ The HER polarization curves normalized by the ECSAs clearly show the highest intrinsic activity of the TS catalyst among the as-synthesized samples (Figures S25–S28). Compared with Pt/C and Pd/C catalysts and other state-of-the-art HER electrocatalysts, the TS catalyst again shows all-around improvement for HER (Figure 2e,h).

*Operando* Raman spectra under HER reveal a newly generated band at 400–600 cm^–1^ on the TS catalyst, corresponding to the *in situ* formation of M–OH and M–O (M = Co, Ni) with the increasing negative potentials, which is negligible in PtPdNiCo/C (Figure S29). The signal strength of M–OH and M–O drops when the negative potential is switched off (Figure 2f), which implies that the water molecules are first adsorbed and then dissociated at the OH vacancy sites of (Ni,Co)(OH)_*x*_, and the adsorbed hydrogen may be transferred to Pt single atoms and Pd clusters by the Volmer–Tafel mechanism.^23−26^ The sluggish water splitting (Volmer step) was largely expedited, and the rate-determining step was transferred to the Tafel step (conversion of two adsorbed *H to H_2_). The TS catalyst showed better long-term stability (100 mA cm^–2^, 200 h) for the HER than Pt/C and Pd/C (Figure S30). Impressively, the polarization potential of the TS catalyst increased by 45 mV after delivering 100 mA cm^–2^ for 200 h, much lower than those of Pt/C (increased 156 mV) and Pd/C (increased 177 mV). It was noted that the polarization potential of the TS catalyst increased with 43 and 50 mV, respectively, at 100 and 380 mA cm^–2^ after the HER for 20,000 cycles, lower than Pt/C and Pd/C (Figure S32). In contrast, the polarization potential of Pt/C increased by 167 mV after cyclic voltammetry scanning for only 5000 cycles (Figure S32). The TS catalyst exhibited the smallest intrinsic resistance and charge transfer resistance, facilitating electron transfer (Figure S33). The increase in the effective surface area was probably due to the full activation of the active sites (Figure S34). It confirms that the interfacial synergism interactions of Pt single atoms, Pd clusters, and (Ni,Co)(OH)_*x*_ nanoparticles (NPs) create cooperative multiple active sites, which are conducive to the optimized HER activity. The phase structures and size of the (Ni,Co)(OH)_*x*_ NPs are well maintained even after the HER for 20,000 cycles (Figures S35 and S36). The exposure of the surface metal content increased on account of the long-term HER measurement (Figure S37).

### 2.4 Electrocatalytic Performance of Coupled MOR and HER

**Figure 3:**
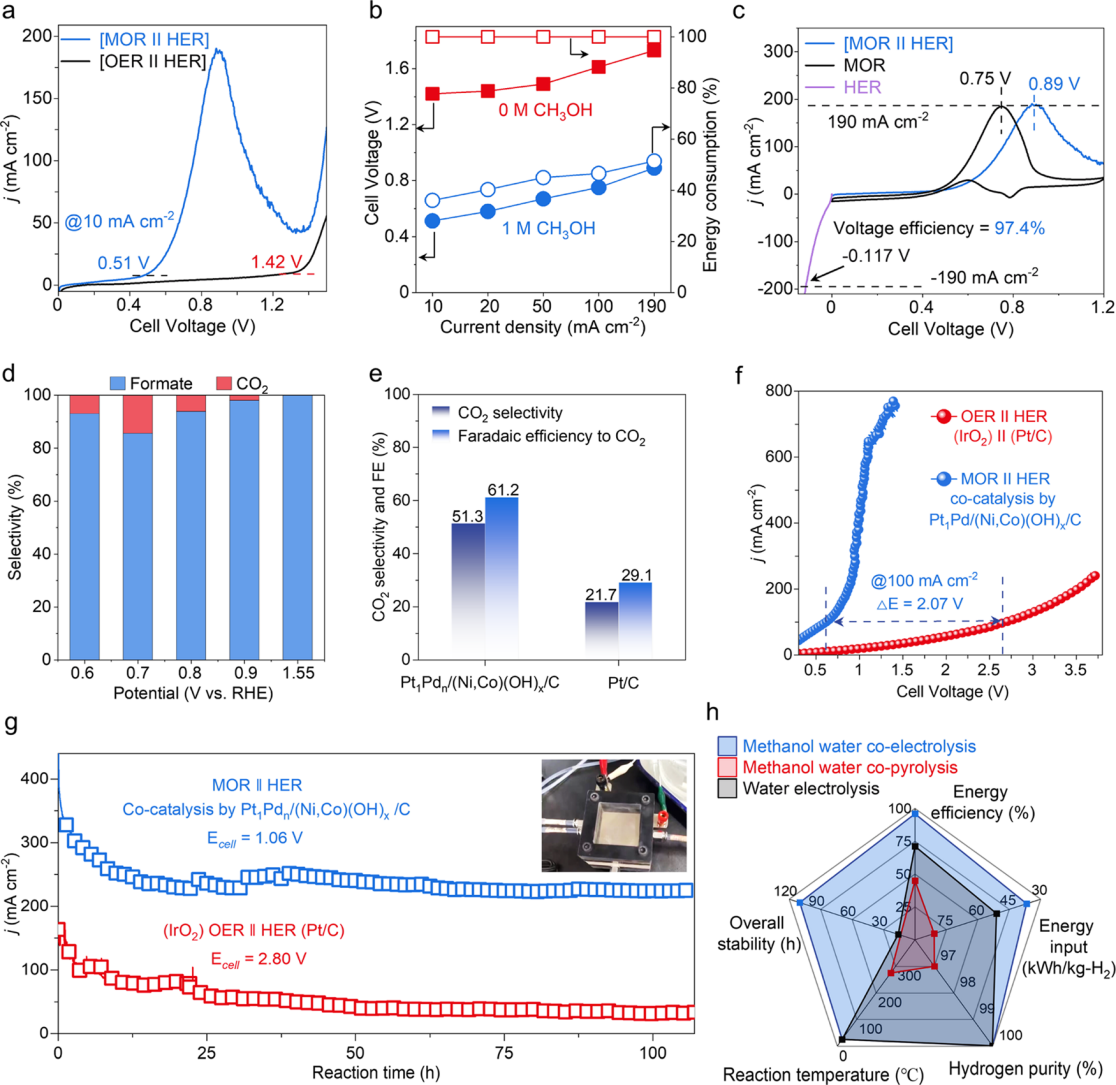
Electrocatalytic performance toward the integrated cell of the TS catalyst coupling MOR and HER. (a) Linear sweep voltammetry curves for the MOR coupling HER and water splitting [OER||HER] electrolytic cell cocatalyzed by the TS catalyst in 1.0 M KOH with/without 1.0 M CH_3_OH in H-type cell, 10 mV s^–1^. (b) Comparison of the coupling cell potentials and the energy consumption of the [MOR||HER] cell and [OER||HER] cell achieving varied current density by the TS catalyst in 1.0 M KOH with/without 1.0 M CH_3_OH. (c) Polarization curve of individual HER and MOR curves, and after coupling to the [MOR||HER] cell. (d) Formate selectivity of the TS catalyst after 1.0 M KOH + 1.0 M CH_3_OH solution (25 mL) electrolysis for the initial 10,000 s at different voltages of 0.600 to 1.550 V (*vs* RHE). (e) CO_2_ selectivity and faradic efficiency (FE) of the TS catalyst and Pt/C while the methanol conversion approached 100% at 0.800 V *vs* RHE. (f) Polarization curves for the [MOR||HER] flow cell and [OER||HER] (water splitting) flow cell cocatalyzed by the TS catalyst in 1.0 M KOH with/without 1.0 M CH_3_OH at a scanning rate of 50 mV s^–1^. (g) Stability test of the TS catalyst under a fixed cell voltage of 1.060 V during 110 h of continuous [MOR||HER] flow cell operation. Reaction conditions: 1.0 M KOH + 1.0 M CH_3_OH solution circulation flow (25 mL min^–1^) in anode for MOR, 1.0 M KOH solution circulation flow (25 mL min^–1^) in cathodic HER. Both anode and cathode were the TS catalysts. (h) Comparison of the three different pathways for hydrogen production.

An H-type cell was first constructed to develop a two-electrode configuration by coating the TS catalyst on carbon paper for both the anode and cathode (Figures 3a and S38). In the H-type cell, the [MOR||HER] required a much lower voltage (0.510 V) than the water-splitting [OER||HER] cell (1.420 V) to reach a reference current density of 10 mA cm^–2^ (Figure 3a,b). The [MOR||HER] cell reached 100 mA cm^–2^ at 0.751 V, corresponding to a voltage reduction/saving of 0.861 V, compared to that of the [OER||HER] cell (1.612 V), which demonstrated the practical viability of the energy-saving [MOR||HER] cell for hydrogen production even at a relatively low voltage (Figure 3b). CO_2_ and H_2_ bubbles were observed at the anode and the cathode chambers, respectively, and CO_2_ was further captured by an additional conversion chamber in 1.0 M Ca(OH)_2_ solution to obtain high-value-added superpure CaCO_3_ (Figure S38). The polarization curves of the HER, MOR, and the coupled [MOR||HER] cell are shown in Figure 3c, where the [MOR||HER] cell exhibits a voltage efficiency of up to 97.4% with a negligible loss after the coupling. Chronopotentiometry responses show enhanced stability of [MOR||HER], in contrast with [OER||HER], at a constant current density of 10 mA cm^–2^ for 10 h (Figure S39).

To further assess the utilization of methanol for hydrogen production, the anodic methanol oxidation products were determined in a three-electrode system, as detailed in the Supporting Information. Nuclear magnetic resonance (NMR) spectra of the TS catalyst confirmed its high formate selectivity (98.03%) and faradic efficiency (91.54%) at 0.900 V for MOR, measured for the initial 10,000 s from 0.600 to 1.550 V (*vs* RHE) (Figure 3d). With methanol conversion increased, the formate intermediate can be further electrooxidized to CO_2_ to impart full utilization of the electronic driving force from methanol. The TS catalyst demonstrated its higher CO_2_ selectivity (51.3%) and faradic efficiency (61.2%) than Pt/C (21.7 and 29.1%, respectively) at 0.800 V *vs* RHE (Figures 3e and S40).

To further evaluate the performance of the catalyst under practical working conditions, the TS catalyst was loaded onto both the anode and the cathode in a [MOR||HER] flow cell (Figure S41). For comparison, an [OER||HER] flow cell was also constructed using the state-of-the-art catalyst IrO_2_ for the OER and Pt/C for the HER, respectively. As noted in Figure 3f, the TS catalyst required a low cell voltage (0.621 V), only 23.1% of that of the [OER||HER] (IrO_2_||Pt/C = 2.690 V), to achieve a current density of 100 mA cm^–2^, and attains a high current density of 700 mA cm^–2^ at only 1.271 V, much superior to previously reported noble and non-noble metal-based bifunctional catalysts for methanol-assisted hydrogen evolution (Tables S5 and S6). The TS catalyst overcomes the limitations of low current density observed commonly for MOR because the synergistic triadic sites of the Pt_1_, Pd*_n_*, and (Ni,Co)(OH)_*x*_ improve the intrinsic MOR activity and suppress the deactivating oxidation of the Pt and Pd species up to a relatively high cell voltage (Figure 3f). Under a fixed cell voltage of 1.060 V, the [MOR||HER] flow cell continuously operated for 110 h at large current density (∼250 mA cm^–2^) with little degradation, which further indicates that both the electro-oxidation of Pt (Pd) to PtO_2_ (PdO) and the likely CO-poisoning issues were effectively suppressed, and confirms its cofunctionality for MOR and HER toward practical current densities at a relatively low cell voltage (Figure 3g). Moreover, the TS catalyst demonstrates strong tolerance to a high concentration of Cl^–^ (0.5 M, simulating the concentration in standard seawater), confirming its potential to facilitate hydrogen production via seawater electrolysis without encountering the competing OER and chlorine evolution reaction (ClER), as a result of the low operating polarizing potential (<1.000 V versus RHE in the anodal MOR) (Figure S42).

The methanol-assisted *in situ* extraction of high-purity hydrogen holds great potential for its distributed applications on-site on demand, particularly for high power-rating hydrogen fuel cells with high overall efficiency (>50%) and voltage output (∼1.000 V), compared with the DMFCs’ ∼30% overall efficiency (low voltage output of ∼0.650 V). The graphical representation of reformed methanol fuel cells (RMFCs) is shown in Figure S43. The methanol and water co-electrolyzer ([MOR||HER]) for hydrogen production shows multiple advantages over both methanol–water “co-pyrolysis,” *i.e.*, thermo-reforming (MOR + HER), and direct water electrolysis ([OER||HER]) (Figure 3h). The key aspects of the improvement of [MOR||HER] over methanol–water thermo-reforming for H_2_ are the mild reaction conditions (NPT—normal pressure and temperature versus HPT—high pressure of 1–5 MPa and temperature of 250–300 °C), lower total energy consumption (36.7 *vs* 80.8 kWh kg^–1^ H_2_), and higher energy efficiency (95.9% *vs* 45.0%). Furthermore, the [MOR||HER] flow cell codriven by the TS catalyst also exhibits a significant “total ME-to-H_2_ energy consumption and efficiency” advantage over the water electrolyzer (IrO_2_)OER ||HER (Pt/C) (36.7 *vs* 51.1 kWh kg^–1^ H_2_, 95.9 *vs* 71.2% energy efficiency). Besides, economic cost/profit calculations estimate that in the simplifying calculations, MOR||HER cell codriven by the TS catalyst could provide hydrogen profit at US$ 5.89 kg^–1^ H_2_ over water electrolysis of (IrO_2_)OER||HER(Pt/C) flow cell (US$ 2.07 kg^–1^ H_2_) and methanol thermal reforming (US$ 1.23 kg^–1^ H_2_) for per kg hydrogen (details are provided in Materials and Methods Section and Table S7 in the Supporting Information).

### 2.5 DFT Simulations for the MOR on (Ni,Co)(OH)_*x*_

**Figure 4:**
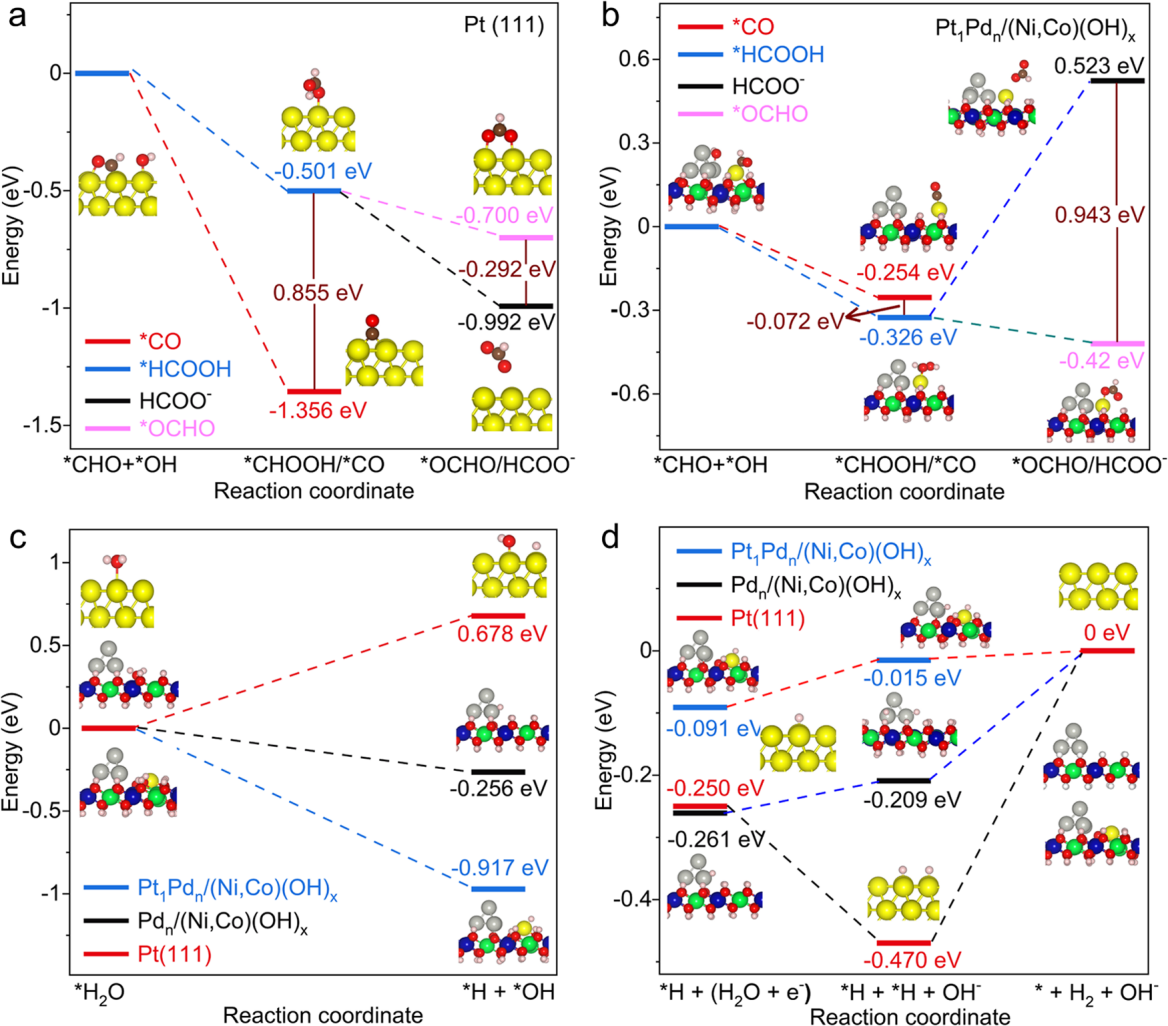
DFT calculation and reaction mechanism of MOR and HER on Pt_1_Pd*_n_*/(Ni,Co)(OH)_*x*_. Free energy diagrams for the formation of *CO and *CHOOH on (a) Pt(111) and (b) Pt_1_Pd*_n_*/(Ni,Co)(OH)_*x*_ for MOR in 1.0 M KOH + 1.0 M CH_3_OH. Free energy diagrams for (c) water dissociation and (d) H_2_ formation on Pt(111), Pd*_n_*/(Ni,Co)(OH)_*x*_, and Pt_1_Pd*_n_*/(Ni,Co)(OH)_*x*_.

To probe the insight into the reaction process, DFT simulations were carried out for possible MOR reaction pathways, as shown in Figure S44. It is well known that CO poisoning is the main drawback for Pt catalysts. Therefore, we investigated the selectivity between two different pathways: *CHO + *OH →: (a) *CO + H_2_O and (b) *CHOOH, for both Pt(111) and the present catalyst (* = possible active site). The energy difference between the two pathways, Δ*G*_1_ = ΔG_*CHOOH_ – ΔG_*CO_, is a fitting descriptor for selectivity (Figure 4a,b). A more negative Δ*G*_1_ suggests a better selectivity toward *CHOOH and vice versa. Pt(111) is more likely to produce *CO rather than *CHOOH because Δ*G*_1_ is as large as 0.86 eV (Figure 4a, and the geometrical structures are shown in Figure S45). Δ*G*_1_ is reduced to 0.348 eV on Pd*_n_*/(Ni,Co)(OH)_*x*_ (Figure S46), which means that the selectivity toward *CHOOH is enhanced, and the corresponding geometrical structures are shown in Figure S47a. Δ*G*_1_ is further decreased to a negative value of −0.072 eV on Pt_1_Pd*_n_*/(Ni,Co)(OH)_*x*_, implying its strong preference for the *CHOOH pathway (Figure 4b, geometrical structures are shown in Figure S47b). Thus, unlike pure Pt, the TS catalyst can effectively suppress the production of *CO and avoid the propensity of CO poisoning to sustain the catalyst activity and enhance the MOR current density. In addition, relatively strong hydrogen bonding is noted between the reaction intermediates and the surface H atoms of (Ni,Co)(OH)_*x*_, which stabilizes the adsorption of the intermediates on the Pd clusters and Pt single atoms (*e.g.*, *CHO, as shown in Figure S47); this is beneficial to multielectron-transfer reactions to further promote the low-voltage MOR current density. Here, OH* is preferably adsorbed on the Pd atomic clusters and then reacts with the intermediates (such as *CHO or *CHOOH) on the Pt single atoms (Figures 4b and S47). Thus, the strong selectivity of *CHOOH, rather than *CO, greatly enhances the MOR catalytic activity (7.796 A mg_PtPd_^–1^) and durability (93.1% initial rate after 4000 s chronoamperometry).

Besides the competition between the formation of *HCOOH and *CO, the selectivity between HCOO^–^ and CO_2_ (*i.e.*, the pathway toward *OCHO) is also investigated. The difference in the free energy change between these two different pathways, Δ*G*_2_ = Δ*G*_HCOO^–^_ – ΔG_*OCHO_, is a good descriptor of this selectivity. As indicated by Figures 4a,b and S46, the production content of HCOO^–^ follows the trend: Pt(111) > Pd*_n_*/(Ni,Co)(OH)_*x*_ > Pt_1_Pd*_n_*/(Ni,Co)(OH)_*x*_. The above trends are consistent with our experimental results for the content of HCOO^–^ for each of the catalysts. Therefore, the coexistence of the Pd nanoclusters and Pt single atoms on (Ni,Co)(OH)_*x*_ can effectively suppress the formation of HCOO^–^.

### 2.6 DFT Simulations for HER on (Ni,Co)(OH)_*x*_

In alkaline media, HER proceeds first via H_2_O dissociation (* + H_2_O + e^–^ → *H + OH^–^), followed by H_2_ production via either the Heyrovsky step (*H + H_2_O + e^–^ → * + H_2_ + OH^–^) or Tafel step (2*H → *+ H_2_). The poor performance of the HER catalytic activity of platinum under an alkaline solution is due to the sluggish H_2_O dissociation step. There are proliferative defects when the (Ni,Co)(OH)_*x*_ was synthesized at a relatively low temperature, which is further enhanced by the “bimetallic tuning” (Figure S4).^21−24^ The surface defects, particularly OH vacancies, are extremely active for water adsorption and then dissociation.^21−24^ The OH defects can capture H_2_O more efficiently than Pt, as shown in Figure S48. The copresence of the Pt single atoms and the Pd nanoclusters adjusts the electronic structures around both species. For Pd*_n_*/(Ni,Co)(OH)_*x*_ and Pt_1_Pd*_n_*/(Ni,Co)(OH)_*x*_, the adsorbed H_2_O (*H_2_O) will dissociate into one *OH at the *V*_OH_ and one *H on the nearest Pd or Pt site, respectively (Figure S48a,b). The atomic structures of this process on Pt(111) are shown in Figure S49. The corresponding free energy variation is presented in Figure 4c, which clearly shows that the sluggish step on Pt(111) (0.678 eV uphill) turns into an exothermic process on Pd*_n_*/(Ni,Co)(OH)_*x*_ (−0.256 eV downhill) and Pt_1_Pd*_n_*/(Ni,Co)(OH)_*x*_ (−0.917 eV downhill). Therefore, both Pd*_n_*/(Ni,Co)(OH)_*x*_ and Pt_1_Pd*_n_*/(Ni,Co)(OH)_*x*_ possess outstanding capability for water dissociation, which is consistent with the fact that this step is no longer rate-limiting, unlike that for Pt(111). As for the Tafel step, the ideal catalyst for HER should possess an appropriate ΔG_*H_ approaching 0 eV. Pd*_n_*/(Ni,Co)(OH)_*x*_ shows better HER catalytic activity (−0.209 eV) than Pt(111) (−0.470 eV), and it is further improved on Pt_1_Pd*_n_*/(Ni,Co)(OH)_*x*_ (−0.015 eV). The Bader effective charges of the samples in Figure 4d are shown in Table S8 to reveal the mechanism behind the excellent HER catalytic activity. Due to the covalent property of the Pt–H bond (Figure S50), the more negative the charge the noble metal species possesses, the more electrons it can offer to form a stronger bond with H. Therefore, Pd*_n_*/(Ni,Co)(OH)_*x*_ and Pt_1_Pd*_n_*/(Ni,Co)(OH)_*x*_ show better performance than Pt(111). In short, (Ni,Co)(OH)_*x*_ with OH vacancies and the Pt_1_, Pd*_n_* sites create favorable local “acid–base” sites. Surface OH vacancy is more “positive” (acidic site) to attract OH^–^ from water, and Pt/Pd is more negative (basic sites) to attract H^+^ coordination to promote H_2_O adsorption and dissociation, and the formation of H_2_ via the Tafel step over the Pt_1_ and Pd*_n_* species (*H_Pt_ + *H_Pd_ → H_2_), and the desorption of *OH occurs simultaneously to regenerate the vacancy site on the hydroxide for the next cycle of HER. The synergistic coordination of the transient species over the triadic sites makes the Pt_1_Pd*_n_*/(Ni,Co)(OH)_*x*_ an excellent catalyst for HER in an alkaline solution. A comparative summary of the capabilities for water dissociation further shows that the TS catalyst possesses the most appropriate water dissociation energy among various reported catalysts to date (Figure S51).

## 3 Conclusions

In summary, the introduction of bimetallic hydroxide (Ni,Co)(OH)_*x*_ largely stabilizes the Pt single atoms and Pd nanoclusters and establishes the intrinsic connection between the two different redox reactions (MOR and HER). The lattice-valence mismatch of Ni and Co promotes the generation and enrichment of OH vacancies over (Ni,Co)(OH)_*x*_. Together with Pt_1_ and Pd*_n_*, the synergistic triadic sites create a favorable “acid–base microenvironment” to facilitate cascade multiple intermediate reaction steps and enable selective pathway regulations, particularly the *CHOOH pathway, instead of the CO, for the MOR, and the dual-site Tafel pathway, rather than the single-site Heyrovsky, for the HER. The mass activity of the TS catalyst for MOR reached 7.796 A mg_PtPd_^–1^, 19.34 times over Pt/C and 38.98 times over Pd/C, respectively. Furthermore, the TS catalyst showed an extremely high HER activity, requiring only an ultralow overpotential of 10 mV to achieve 10 mA cm^–2^, with a Tafel slope of merely 19.8 mV dec^–1^. The “methanol and water co-electrolysis” fuel processing system ([MOR||HER]) flow cell at 1.060 V was demonstrated to work stably at a large current density of ∼250 mA cm^–2^ for more than 110 h with little degradation. This work offers an effective strategy for green hydrogen production at an energy-efficient low-cell-voltage “on-site on demand at ambient” with the assistance of a safe and convenient liquid hydrogen carrier, methanol.

## Supporting Information Available

Additional experimental details; materials and methods, including schematic illustration of Pt_1_Pd*_n_*/(Ni,Co)(OH)_*x*_/C; AC-STEM, XRD, EPR, XANES, EXAFS, Fourier-transformed EXAFS data and the best-fit results, Best-fit EXAFS parameters, XPS, HS-LEIS; CO-stripping experiments data; *In situ* FTIR data and spectra, NMR data and spectra; *operando* Raman data and spectra; electrochemical measurements and data; energy consumption and economic calculation; DFT details and calculations; and product analysis (PDF)
MOR Mechanism (MP4)
HER Mechanism (MP4)

## Abbreviations

ME: methanol
Pt_1_: Pt single atoms
Pd_n_: Pd nanoclusters
V_OH_: OH-vacancy
MOR: methanol oxidation reaction
HER: hydrogen evolution
DMFC: direct methanol fuel cell
RMFC: reformed methanol fuel cell
TS catalyst: triadic sites catalyst
EPR: electron paramagnetic resonance
CN: coordination number
MA: mass activity
OER: oxygen evolution reaction
HS-LEIS: high-sensitivity low-energy ion scattering spectra
CR: relative concentrations
NPs: nanoparticles
NMR: nuclear magnetic resonance
ClER: chlorine evolution reaction
NPT: normal pressure and temperature
HPT: high pressure and temperature
RHE: reversible hydrogen electrode
DFT: density functional theory
AC-STEM: aberration-corrected scanning transmission electron microscope
XRD: X-ray diffraction
EXAFS: Extended X-ray absorption fine structure
XPS: X-ray photoelectron spectroscopy
XANES: X-ray absorption near-edge structure
ECSA: electrochemical active surface areas
FE: faradic efficiency
OCP: open-circuit potential
