# Supplementary material for: Methanol-Enhanced Low-Cell-Voltage Hydrogen Generation at Industrial-Grade Current Density by Triadic Active Sites of Pt1–Pdn–(Ni,Co)(OH)x
Source: J Am Chem Soc. 2025 Jan 13;147(4):3185–94. doi: 10.1021/jacs.4c12665 (PMC11803621; doi:10.1021/jacs.4c12665)
Supplement: Supplementary file 1 — ja4c12665_si_001.pdf [file ja4c12665_si_001.pdf]

Supporting Information

Methanol enhanced low-cell-voltage hydrogen generation at industrial-grade current density by triadic active sites of Pt_1–Pd_n–(Ni,Co)$(OH)_x$

An Pei[1, #], Ruikuan Xie[3, #], Lihua Zhu[1, 2, #, *], Fengshun Wu[1], Zinan Huang[5], Yongyu Pang[3], Yu-Chung Chang[6], Guoliang Chai[3, *], Chih-Wen Pao[6], Qingsheng Gao[5, *], Congxiao Shang[2], Guang Li[4], Jinyu Ye[4], Huaze Zhu[7], Zhiqing Yang[7], Zhengxiao Guo[2, *]

[1] Jiangxi Province Key Laboratory of Functional Crystalline Materials Chemistry, College of Chemistry and Chemical Engineering, Faculty of Materials Metallurgy and Chemistry, Jiangxi University of Science and Technology, Ganzhou 341000, Jiang Xi, China.

[2] Department of Chemistry, The University of Hong Kong, Hong Kong Island 000000, Hong Kong SAR, China.

[3] State Key Laboratory of Structural Chemistry, Fujian Institute of Research on the Structure of Matter, Chinese Academy of Sciences (CAS), Fuzhou 350002, Fujian, China.

[4] State Key Laboratory for Physical Chemistry of Solid Surfaces, College of Chemistry and Chemical Engineering, Xiamen University, Xiamen 361005, Fujian, China.

[5] College of Chemistry and Materials Science, and Guangdong Provincial Key Laboratory of Functional Supramolecular Coordination Materials and Applications, Jinan University, Guangzhou 510632, China.

[6] National Synchrotron Radiation Research Center, Hsinchu 300, Taiwan.

[7] Ji Hua Laboratory, Foshan 528200, China.

[#] These authors contributed equally to this work.
* Corresponding authors.
Email: zhulihua@jxust.edu.cn (Lihua Zhu); g.chai@fjirsm.ac.cn (Guoliang Chai); tqsgao@jnu.edu.cn (Qingsheng Gao); zxguo@hku.hk (Zhengxiao Guo)

Materials and Methods

Materials

The chemicals used in the experiments were of analytical grade. Chloroplatinic acid hexahydrate ($H_2PtCl_6 \cdot 6H_2O$, 37.5 wt% Pt), ammonium tetrachloropalladate ($(NH_4)_2PdCl_4 \cdot H_2O$, 36.5 wt% Pd), nickel (II) chloride hexahydrate ($NiCl_2 \cdot 6H_2O$, >98%), cobalt (II) chloride hexahydrate ($CoCl_2 \cdot 6H_2O$, >98%), potassium hydroxide (KOH, 99.9%), and methanol (CH_3OH, >99.9%) were purchased from Aladdin. Hydrazine hydrate (85 wt%) was obtained from Sinopharm Chemical Reagent Co., Ltd. (Shanghai, China). Nafion (5 wt%) was obtained from Shanghai Huiying Biotechnology Co., Ltd. The reagents were not purified prior to the experiments. Ultrapure water was prepared using Millipore A10 Milli-Q systems. High-purity nitrogen gas was procured from Ganzhou Jianli Gas Co., Ltd. The catalyst support carbon black (BLACK PEARLS 2000 LOT-1366221) was purchased from Cabot Corporation.

Preparation of NiCo/(Ni,Co)(OH)$_x$/C, Ni/Ni(OH)$_x$/C, Co/Co(OH)$_x$/C

Firstly, $NiCl_2 \cdot 6H_2O$ (0.5625 g) and $CoCl_2 \cdot 6H_2O$ (0.5625 g) solutions in a conical flask were ultrasonically dissolved in deionized water (82.5 mL) for 10 min. Then, the solution was magnetically stirred in an ice bath (5 °C); after 15 min, anhydrous ethanol (12.5 mL) was injected and magnetically stirred for another 10 min. After that, carbon black (BP-2000, 1.2500 g) was added and ultrasonically dispersed for 20 min, followed by magnetically stirring for 30 min in an ice bath (5 °C). The rapid nucleation of Ni^{2+}/Co^{2+} species adsorbed on BP-2000 was realized by the gradual dropping of 3.6 M NaOH solution (12.5 mL) in an ice bath (5 °C), to form small (Ni,Co)(OH)$_x$ nanoplatelets over the carbon black. After that, hydrazine hydrate (25 mL) was dropwise added into the mixture to induce the *in-situ* partial reduction of Ni^{2+}/Co^{2+} to Ni^0/Co^0 over the surface of (Ni,Co)(OH)$_x$ in an ice bath (5 °C) to obtain

$NiCo/(Ni,Co)(OH)_x/C$. The Ni^{2+}/Co^{2+} reduction process was very slow, and the reaction solution was transferred into the 300 mL autoclave with mechanical stirring in an ice bath (5 ℃) for 18.5 h. $NiCo/(Ni,Co)(OH)_x/C$ was obtained by filtration, washed five times with the mixture of anhydrous ethanol/deionized water (1/1), and dried in a vacuum (60 ℃, 8 h). The $Ni/Ni(OH)_x/C$ ($Co/Co(OH)_x/C$) was synthesized by changing the species of the metal precursor, and then following the other steps described above.

Synthesis of $Pt_aPd_b/(Ni,Co)(OH)_x/C$, $Pt_{a+b}/(Ni,Co)(OH)_x/C$, $Pd_{a+b}/(Ni,Co)(OH)_x/C$

For instance, $Pt_aPd_b/(Ni,Co)(OH)_x/C$ with the atomic ratio of Pt/Pd = a/b = 1:1, $Pt_1Pd_1/(Ni,Co)(OH)_x/C$ also denoted as $Pt_1Pd_n/(Ni,Co)(OH)_x/C$, the $NiCo/(Ni,Co)(OH)_x/C$ (0.1200 g) sample was firstly suspended in deionized water (200 mL), magnetically stirred for 15 min in an ice bath (5 ℃). Then, aqueous $H_2PtCl_6{\cdot}6H_2O$ solution (3.75 mL, 7.7235 mM) was further diluted with deionized water by 10 times to 0.77235 mM, and it was dropwise added with a drip funnel (one drop/5 s) into the solution with vigorous stirring in an ice bath for 2 h (5 ℃). After that, the catalyst was obtained by filtration and redispersed in 200 mL deionized water, aqueous $(NH_4)_2PdCl_4{\cdot}H_2O$ solution (1.50 mL, 33.0810 mM) was dropwise (one drop/2 s) added into the above solution at room temperature (25 ℃). After vigorous stirring for 2 h, the sample was filtrated and washed five times in the mixture of anhydrous ethanol/deionized water (1/1) solution and dried in vacuum (60 ℃, 8 h), signed as $Pt_aPd_b/(Ni,Co)(OH)_x/C$ (a: b was the atomic ratio). $Pt_1/(Ni,Co)(OH)_x/C$ (or $Pd_n/(Ni,Co)(OH)_x$) was synthesized by replacing the noble metal precursor with $H_2PtCl_6{\cdot}6H_2O$ (or $(NH_4)_2PdCl_4{\cdot}H_2O$). $Pt_1Pd_n/Ni(OH)_x/C$ ($Pt_1Pd_n/Co(OH)_x/C$) samples were synthesized by replacing $NiCo/(Ni,Co)(OH)_x/C$ with $Ni/Ni(OH)_x/C$ ($Co/Co(OH)_x/C$), and the other steps followed the above descriptions as for $Pt_aPd_b/(Ni,Co)(OH)_x/C$.

Preparation of PtPdNiCo/C and PtPd/C

PtPdNiCo/C was prepared after $Pt_1Pd_n/(Ni,Co)(OH)_x/C$ (0.1000 g) being reduced in 90%N_2+10%H_2 at 400 °C for 2 h. PtPd/C was synthesized through the impregnation method, carbon black (0.3000 g) was suspended in 100 mL ultrapure water and then vigorously stirred for 0.5 h. Then, the aqueous $H_2PtCl_6 \cdot 6H_2O$ solution and $(NH_4)_2PdCl_4 \cdot H_2O$ solution were added and stirred for 1 h. After that, the solution was evaporated in a water bath at 80 °C to gain the black solid powder. The solid was reduced in 90%N_2+10%H_2 at 400 °C for 2 h to obtain the PtPd/C catalyst.

Characterization of the samples

The powder X-ray diffraction tests were acquired by employing the Rigaku Ultima IV X-ray diffractometer with monochromatized Cu *Ka* radiation (5°~90°, 40 kV, 30 mA). The X-ray photoelectron spectroscopy spectra were recorded using a PHI Quantum 2000 Scanning ESCA Microprobe instrument with Al K_a radiation working at 1486.6 eV. The light/heavy elements of the catalyst surface was further characterized by high sensitivity-low energy ion scattering (HS-LEIS) spectroscopy (IonTOF Qtac100) equipped with the ion beam (3 k eV He^+, 5 k eV 20 Ne^+) as the sputtering ion source. The measurements for the precise metal contents of the as-synthesized catalysts were performed by the inductively coupled plasma mass spectrometry (ICP-MS) technique.

The morphologies and nanostructures of various catalysts were investigated through the TEM and HRTEM (TECNAI F30, FEI) techniques working at an accelerating voltage of 300 kV. The AC-STEM images and AC-STEM-EDS elemental maps were obtained by using Titan FEI Titan Themis 60-300 aberration-corrected microscope operated at 200 kV.

X-ray absorption spectra (XANES, EXAFS).

The structure characterization of all samples was determined by the XANES and EXAFS in TPS BL44A, National Synchrotron Radiation Research Center (Taiwan). Ni and Co *K*-edge XAS spectra were collected in transmission mode, and Pt L_3-edge and Pd *K*-edge XAS spectra were done in fluorescence mode. Energy calibration was done by using Ni, Co, Pt, and Pd foil as references. Spectra energy resolution could be set at ~0.3 eV, ~0.4 eV, ~0.5 eV, and ~1.2 eV at a photon energy of 7112 eV (Co *K*-edge), 8333 eV (Ni *K*-edge), 11564 eV (Pt L_3-edge), and 24350 eV (Pd *K*-edge), respectively.

Electrochemical *in-situ* FTIR spectra.

Electrochemical *in-situ* Fourier transform infrared (FTIR) reflection spectroscopy tests were conducted through a Nicolet-8700 spectrometer combined with a liquid-nitrogen-cooled MCT-A detector. The measurement of adsorbed species and dissolved species was conducted on a thin layer (< 10 mm) between the working electrode and CaF_2 window for *in-situ* MSFTIR. The working electrodes were prepared as the same method as the MOR measurements and electrochemically activated until stable in N_2-saturated 1.0 M KOH electrolyte. Then, the electrochemical FTIR was measured in 1.0 M KOH + 1.0 M CH_3OH solution. Multi-stepped FTIR spectroscopy (MS-FTIR) was exploited to collect spectra from 0.200 V to 1.200 V versus RHE at 0.1 V intervals. The relative change in reflectivity (ΔR/R) towards spectra was calculated by the following equation:

$$\frac{\Delta R}{R} = \frac{R(E_S) - R(E_R)}{R(E_R)} \quad (1)$$

$R(E_S)$ represented the catalysts' potential E_S collected by single-beam spectra, and E_R is the reference potential.

Electrochemical *Operando* Raman spectra.

The electrochemical *Operando* Raman measurements were performed using a Horiba HR-800 Raman microspectrometer including a typical electrochemical Raman spectroscopy setup combined with a CHI 650 electrochemical workstation. The 1 × 1 cm^2 carbon paper-covered catalysts were adopted as the working electrode, an Hg/HgO was employed as the reference electrode and the counter electrodes were a graphite rod. A proton exchange membrane (Nafion 117, Sigma-Aldrich) was applied for the separator experiments towards a two-compartment electrochemical cell in a 1.0 M KOH solution. Raman spectra at different times or potentials were recorded during the CA tests.

Electrochemical measurements

An electrochemical workstation (CHI, 760E) was used for all electrochemical measurements. The evaluation of the electrocatalytic performance of the as-prepared catalysts was carried out in a three-electrode system. For the MOR, catalysts coatings on glassy carbon electrode were employed as the working electrode, Pt foil-counter electrode, a saturated calomel electrode (SCE)-reference electrode, and the N_2-saturated 1.0 M KOH + 1.0 M methanol served as electrolyte. Curves were obtained by a scan rate of 50 mV s^{-1}. The long-term stability of chronoamperometry for 4000 s was realized by holding the constant potential at -0.300 V versus SCE to measure the change of current. For the CO-stripping measurements, a 20% CO/N_2 gas was flowing in 1.0 M KOH for 15 min, then, the monolayer of CO adsorbed on the samples achieved by holding the electrode potential at -0.960 V versus SCE in 1.0 M KOH for 1 h. Later, the non-adsorbed CO in the 1.0 M KOH solution was removed by bubbling with N_2 for 0.5 h. After that, the CV curves of CO-stripping were recorded by measuring at 0 V-1.250 V versus RHE. The electrochemical active surface areas (ECSAs) were evaluated by the CO electrooxidation peak area integral results.

Before the HER measurement, carbon paper-covered catalysts (1*1 cm^2, 2 mg) were utilized as the

working electrode, a graphite rod-counter electrode, a saturated calomel electrode (SCE)-reference electrode, and the N_2-saturated 1.0 M KOH served as the electrolyte, respectively. The potential was calculated concerning RHE using the equation:

$$E(RHE) = E(SCE) + 0.0591 \times pH + 0.2415. \quad (2)$$

The pH value of the HER electrolyte was recorded as 13.8. The SCE reference electrode was calibrated by applying a Pt foil as the working electrode. The HER polarization curves were gained via a scan rate of 5 mV s^{-1}. Two different methods for long-term stability measurements of the HER were adopted. One method was the CV curves of long-term stability of 20000 circles measured at a scan rate of 50 mV s^{-1}, the other method was the chronopotentiometry at 100 mA cm^{-2} for 200 h. After the open-circuit voltage was determined, the electrochemical impedance spectroscopy (EIS) was conducted in the frequency range of 0.1-100,000 Hz. Besides, the double-layer capacitance (C_{dL}) was estimated by evaluating the CV measurements at different scanning rates from 10 to 50 mV s^{-1}. The cell of MOR coupling HER (MOR || HER) co-catalyzed by $Pt_1Pd_n/(Ni,Co)(OH)_x/C$ was carried out in the H-type cell, the electrolyte was 1.0 M KOH + 1.0 M methanol in the anode, and 1.0 M KOH in the cathode. The CO_2 generated in the anode was further captured by the additional device of 1.0 M $Ca(OH)_2$ solution to obtain the high value-added product ($CaCO_3$). The carbon papers covered with the $Pt_1Pd_n/(Ni,Co)(OH)_x/C$ catalyst (0.5×1 cm^2, 1.0 mg) were utilized as the working electrode. The working electrode was firstly activated for 20 circles in the three-electrode system before the LSV measurements of the two-electrode system MOR||HER cell. For comparison, the device of water splitting (OER||HER) was identical to MOR||HER, but the electrolyte was 1.0 M KOH both in the anode and cathode. The polarization curves were measured from 0 V to 1.700 V at 10 mV s^{-1}. The long-term chronopotentiometry response measurements towards the MOR||HER cell and OER || HER cell were conducted at a constant current density of 10 mA cm^{-2} for 10 h.

Energy consumption and economic cost/profit calculation.

Energy consumption calculation.

To reduce energy costs for hydrogen production, the battery voltage should be reduced to an acceptable value by designing and synthesizing new electrocatalysts with higher activity and selectivity[1-3]. In fact, according to Equation (3), the energy consumed was proportional to the cell voltage:

$$We(\text{in Kwh/N}m^3) = nF/(3600\ V_m \times 10^3)\ \times\ U_{cell}(\text{j}) \qquad (3)$$

For an ideal gas under normal conditions (T = 273.15 K, P = 1 atm = 101.325 kPa) the molar volume is V_m = 22.414 × 10^{-3} m^3 mol^{-1}, U_{cell} (j) is the cell voltage of a certain current density. F = 96485 C per mole of the electron was the Faraday constant and n is the average number of per mole of hydrogen involved in the overall process (n = 2). So that We = 2.39 kWh $(Nm^3)^{-1}$ per Volt since the electrical energy consumed only depended on the cell voltage U_{cell}, U_{cell} should be below 1.000 V to decrease the energy consumed below 2.4 kWh $(Nm^3)^{-1}$. At standard conditions (1 atmosphere, 0 °C), the density of hydrogen is 0.0899 kg/m^3.

Therefore, We = 1kg_{H2}/0.0899 kg/m^3 × 2.3915 kWh $(Nm^3)^{-1}$ × U_{cell} = 26.602 kWh/kg_{H2} × U_{cell}(j)

MOR||HER cell co-driven by $Pt_1Pd_n/(Ni,Co)(OH)_x/C$ for per kg hydrogen:

There being higher and lower heating values of per kg_{H2} and methanol, median heating value (130.9 MJ/kg H_2 = 36.36 kWh/kg H_2, 19.05 MJ/kg methanol = 5.29 kWh/kg methanol) was used in this work. For simplification purposes, the methanol conversion and carbon dioxide selectivity are assumed to be 100%, and the electrolysis was conducted at the corresponding cell voltage for the actual current density achieving and stabilizing at 50 mA cm^{-2}, *and* U_{cell} at 50 mA cm^{-2} was 0.320 V.

Electric power consumption (We) = 26.602 kWh/kg_{H2} × U_{cell} = 26.602 kWh/kg_{H2} ×0.320 V = 8.51 kWh/kg H_2

Total heating value of methanol = 5.33 kg * (5.29 kWh/kg methanol) = 28.20 kWh/kg H_2

Total energy consumption = Electric power consumption (We) + Total heating value of methanol = 36.71 kWh/kg H_2

Total energy efficiency = Theoretical energy consumption / Practical energy consumption = (heating value of /kg H_2 - Total heating value of methanol)/ Electric power consumption = (36.36 kWh/kg H_2 – 28.20 kWh/kg H_2) /8.51 kWh/kg H_2 = 95.9%

Water electrolysis of (IrO_2) OER||HER (Pt/C) cell for per kg hydrogen:

For simplification purposes, electrolysis was conducted at the corresponding cell voltage for the actual current density achieving and stabilizing at 50 mA cm^{-2}.

Due to the heating value of H_2O is zero, thus total power/energy consumption = Electric power consumption (We) = 26.602 kWh/kg_{H2}× U_{cell} = 26.602 kWh/kg_{H2} ×1.920 V (U_{cell} at 50 mA cm^{-2}) = 51.08 kWh/kg H_2

Total energy efficiency = Theoretical energy consumption / Practical energy consumption = (heating value of /kg H_2)/ Electric power consumption = (36.36 kWh/kg H_2) /51.08 kWh/kg H_2 = 71.2%

Methanol thermal reforming for per kg hydrogen:

Conventional methanol steam-reforming for H_2 production (Gray hydrogen) (CH_3OH (g) + H_2O (g) = CO_2 (g) + 3 H_2 (g) ΔH = + 49.4 kJ mol^{-1}) requires high temperature (250-300 °C) and pressure (1-5 MPa), along with the release of CO_2, CO, HCOOH, and CH_3OH (unreacted) steam. The reformer operating at 350 °C with a steam/carbon ratio of 1.8, high CH_3OH (heating value was ~ 2.27 *10^4 kJ kg^{-1}) conversion (> 99%) and CO (0.8%) was confirmed in the dry reformate with a net thermal efficiency of 45%[2,4].

Therefore, ignoring carbon balance loss and hydrogen recovery rate, the energy input per kg gray hydrogen (heating value of hydrogen is 36.36 kWh/kg H_2) is lowest: 36.36 kWh/kg H_2 / 45% = 80.80 kWh/kg H_2.

Thereby, MOR||HER cell co-driven by $Pt_1Pd_n/(Ni,Co)(OH)_x/C$ catalyst exhibits significant "Total Energy Consumption and efficiency" advantages (36.71 kWh/kg H_2, 95.9% energy efficiency) over water electrolysis of (IrO_2) OER||HER (Pt/C) cell (51.08 kWh/kg H_2, 71.2% energy efficiency) and Methanol thermal reforming (80.80 kWh/kg H_2, 45.0% energy efficiency) for per kg hydrogen.

Economic cost/profit estimations.

MOR||HER cell co-driven by $Pt_1Pd_n/(Ni,Co)(OH)_x/C$ for per kg hydrogen:

For the sake of simplifying calculations and highlighting the inherent characteristics of the reaction coupling itself, the costs of device, catalysts and electrolytes are not included in the cost accounting scope.

Economic cost (US$/ kg H_2) = Cost of electric power consumption (8.51 kWh/kg H_2 * US$ 0.086 per kWh = US$ 0.7320) + Cost of Methanol (5.33 kg * US$ 0.205/kg = US$ 1.0927) + Cost of Water (3.0 kg

* US$ 0.59/tonne = US$ 0.0018) + Cost of $Ca(OH)_2$ (12.33 kg * US$ 0.0575/kg = US$ 0.7090) = US$ 2.5355/kg H_2

Main products value (US$/ kg H_2) = value of Green H_2 (1 kg * US$ 6.1434/kg H_2) + value of $CaCO_3$ (16.67 kg * US$ 0.137/kg $CaCO_3$ = US$ 2.28379/kg H_2) = US$ 8.4272/kg H_2

**Economic Profit (US$/kg$H_2$) =** Main products value (US$/kg H_2) - Economic cost (US$/kg H_2) = US$ 5.8917/kg H_2

Water electrolysis of (IrO_2) OER||HER (Pt/C) cell for per kg hydrogen:

For the sake of simplifying calculations and highlighting the inherent characteristics of the reaction coupling itself, the costs of devices, catalysts and electrolytes are not included in the cost accounting scope.

Economic cost (US$/ kg H_2) = Cost of electric power consumption (51.08 kWh/kg H_2 * US$ 0.086 per kWh = US$ 4.3929) + Cost of Water (9.0 kg * US$ 0.59/tonne = US$ 0.0054) = US$ 4.3983/kg H_2

Main products value (US$/ kg H_2) = value of Green H_2 (1 kg * US$ 6.1434/kg H_2) + value of O_2 (16 kg * US$ 0.02/kg O_2 = US$ 0.32/kg H_2) = US$ 6.4634/kg H_2

Economic Profit (US$/kg H_2) = Main products value (US$/kg H_2) - Economic cost (US$/kg H_2) = US$ 2.0651/kg H_2

Methanol thermal reforming for per kg hydrogen:

For the sake of simplifying calculations and highlighting the inherent characteristics of the reaction coupling itself, the costs of catalysts and devices are not included in the cost accounting scope.

Economic cost (US$/kg H_2) = Cost of Thermal power consumption (US$ 1.1755) + Cost of Methanol (5.33 kg * US$ 0.205/kg = US$ 1.0927) + Cost of Water (3.0 kg * US$ 0.59/tonne = US$ 0.0018) = US$ 2.27/kg H_2

Main products value (US$/ kg H_2) = value of Grey H_2-95% purity (1 kg * US$ 3.4954/kg H_2)

Economic Profit (US$/kg H_2) = Main products value (US$/kg H_2) - Economic cost (US$/kg H_2) = US$ 1.2254/kg H_2

Thereby, MOR||HER cell co-driven by $Pt_1Pd_n/(Ni,Co)(OH)_x/C$ catalyst exhibits significant " **Economic Profit**" advantages (US$ 5.8917/kg H_2) over water electrolysis of (IrO_2)OER||HER(Pt/C) cell (US$ 2.0651/kg H_2) and Methanol thermal reforming (US$ 1.2254/kg H_2) for per kg hydrogen.

Product analysis

For MOR, the concentrations of organics in the electrolytes were analyzed by ^{1}H nuclear magnetic resonance (NMR) spectroscopy (ECZ400S). The electrocatalysts (5 mg) were dispersed in a solution containing 0.5 mL isopropyl alcohol, 0.5 mL ultra-pure water, and 30 μL Nafion solution (5%, DuPont) to form an ink solution. Then, 257.5 μL ink solution was deposited dropwise on a hydrophilic carbon paper as the working electrode, and Pt foil and a saturated calomel electrode (SCE) were used as the reference and counter electrodes, respectively. The N_2-saturated 1.0 M KOH + 1.0 M methanol served as an electrolyte. After a certain period of electrolysis, the corresponding electrolyte was used for NMR. The NMR sample was prepared as follows: 0.5 mL electrolyte, 0.15 mL D_2O, and 0.1 mL 0.5 μL mL^{-1} DMSO aqueous solution (used as internal standard) were mixed. The FE of products was calculated as follows:

$$\mathrm{FE} = \frac{nCVF}{\mathrm{Q}} \times 100\% \tag{4}$$

where n was the number of transferred electrons, V was the volume of electrolyte, C was the concentration of the product, F was the Faraday constant, and Q was the accumulated charge.

To investigate the selectivity of the catalyst at very high conversion rates, we tested the complete reaction at 0.8 V. CO_2 generated in the anode was derived and captured through the external device contained with 1.0 M $Ca(OH)_2$ solution to acquire the high value-added product of $CaCO_3$.

DFT calculations

All the density functional theory (DFT) calculations were performed via the Vienna Ab initio Simulation Package (VASP),[5-8] and the projector-augmented plane wave (PAW) pseudopotentials were used for the elements involved[9] The generalized gradient approximation (GGA) of Perdew, Burke, and Ernzerhof (PBE) was used to treat the exchange correlation between electrons[10] The $(Ni,Co)(OH)_x$ slabs adsorbed with Pd cluster and Pt atom calculated in this study are shown in Fig. S47. A vacuum region larger than 15 Å was added along the direction normal to the slab plane to avoid the interaction between periodic supercells. The electron wave function is expanded in plane waves and a cutoff energy of 500 eV was chosen. The Monkhorst-Pack meshes of (3×3×1) were adopted for the Brillouin zone (BZ) of the slabs[11]. The convergence in the energy and force were set to 10^{-4} eV and 0.01 eV/Å, respectively.

The free energies of $H_2O(l)$ and $H_2(g)$ were used as references when calculating the free energies of

reaction intermediates. The adsorption energy for the reaction intermediate is calculated as follows:[12]

$$\Delta G = \Delta E_{Total} + \Delta E_{ZEP} - T\Delta S \quad (5)$$

where ΔE_{Total} is the calculated adsorption total energy by DFT, ΔE_{ZPE} is zero-point energy, ΔS is entropy, and ΔGs is solvation energy[13-15].

The calculated limiting potential for HER can be obtained as follows:

$$U_L = Mini[-\Delta G_i] / ne \quad (6)$$

Where n is the number of electrons transferred for each electrochemical step, and e is the elementary charge. Here, the n is set to 1 for the one-electron transfer step. The meaning of the *r.h.s.* of the above equation is to select the smallest $[-\Delta G_i]$ among the HER elementary steps.

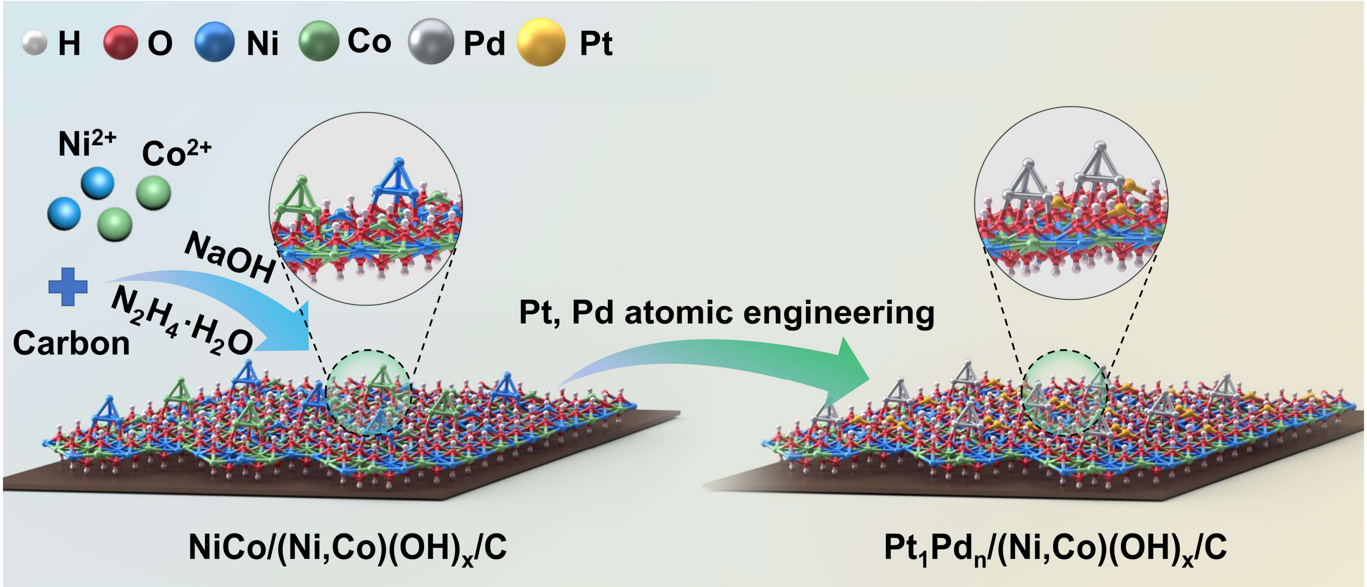

Figure S1. Schematic illustration of the fabrication for the $Pt_1Pd_n/(Ni,Co)(OH)_x/C$. The $NiCo/(Ni,Co)(OH)_x/C$ support was synthesized via a liquid-phase chemical reduction method. The Pt single atoms and Pd clusters were anchored by $(Ni,Co)(OH)_x$ successively by galvanic replacement in an ice-water bath (See Methods).

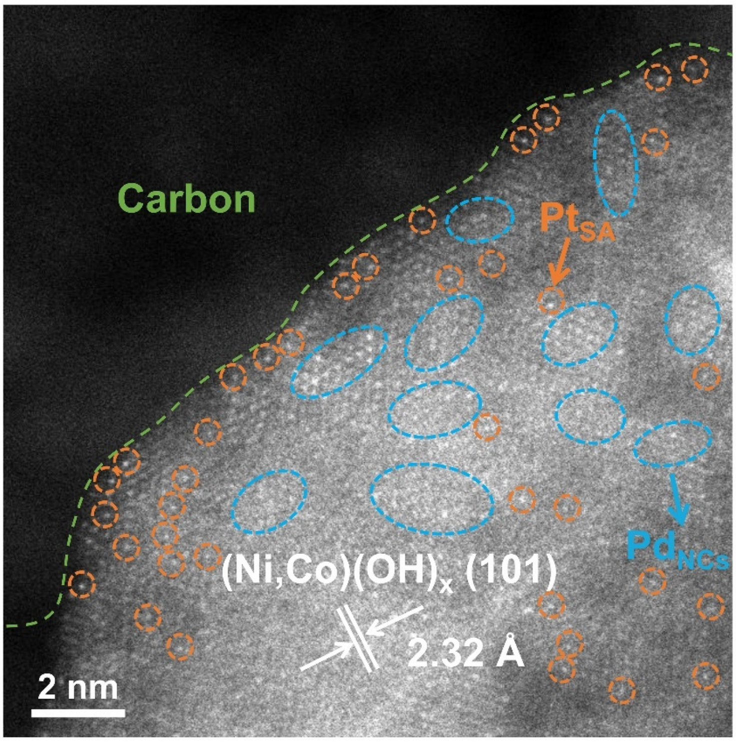

Figure S2. Representative AC-STEM image of Pt_1Pd_n/(Ni,Co)(OH)$_x$/C. The nanostructures of Pt single atoms and Pd nanoclusters on (Ni,Co)(OH)$_x$ nanoparticles, are further supported by carbon black.

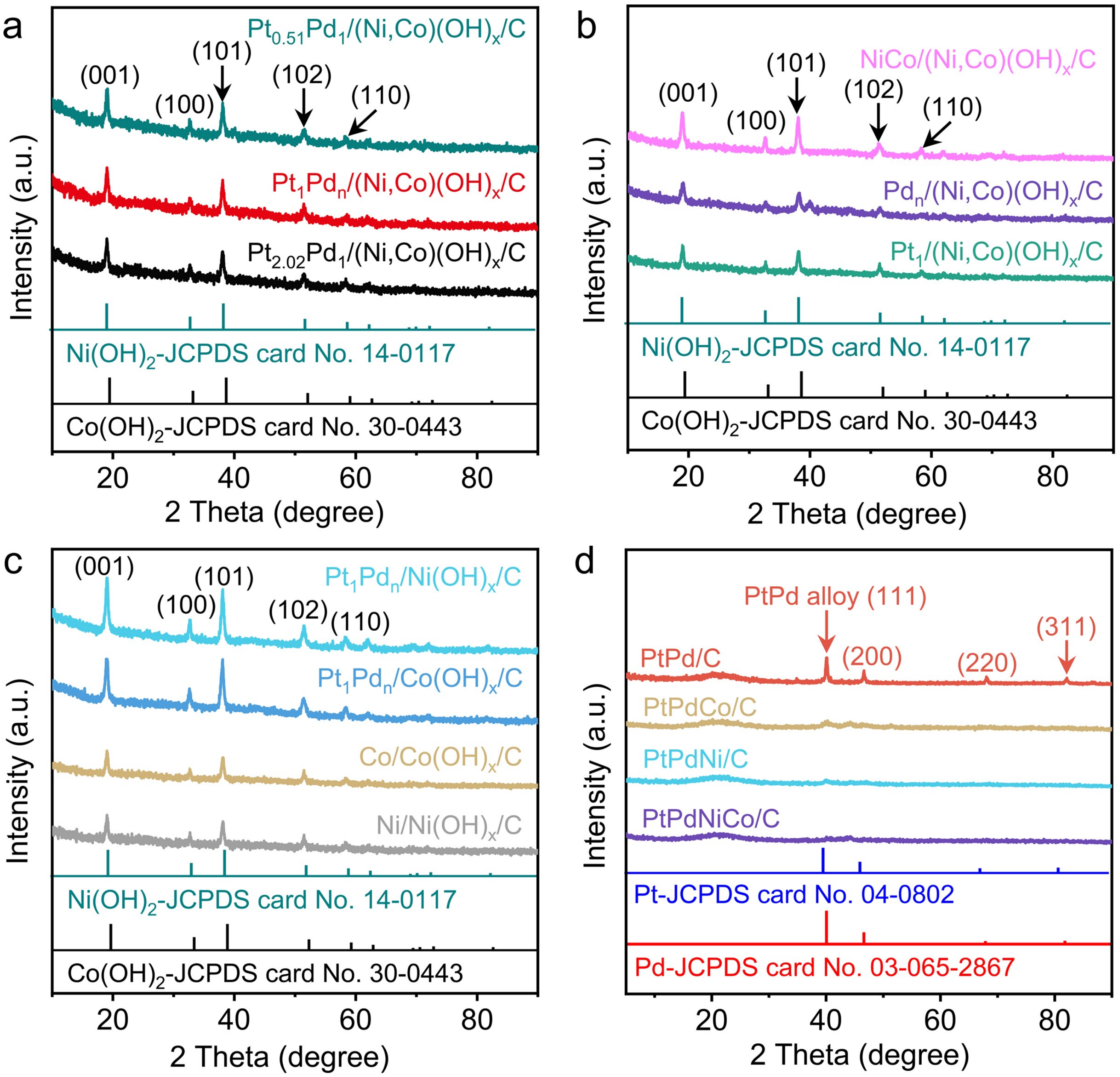

Figure S3. XRD patterns for the as-prepared samples. XRD patterns of **(a)** $Pt_{0.51}Pd_1/(Ni,Co)(OH)_x/C$, $Pt_1Pd_n/(Ni,Co)(OH)_x/C$, $Pt_{2.02}Pd_1/(Ni,Co)(OH)_x/C$. **(b)** $NiCo/(Ni,Co)(OH)_x/C$, $Pd_n/(Ni,Co)(OH)_x/C$, $Pt_1/(Ni,Co)(OH)_x/C$. **(c)** $Pt_1Pd_n/Ni(OH)_x/C$, $Pt_1Pd_n/Co(OH)_x/C$, $Co/Co(OH)_x/C$, $Ni/Ni(OH)_x/C$. **(d)** PtPd/C, PtPdCo/C, PtPdNi/C, PtPdNiCo/C. There are only signals for the hydroxide support, but no peaks from the Pt or Pd species in the XRD patterns of $Pt_1Pd_n/(Ni,Co)(OH)_x/C$, indicating that Pt and Pd are extremely fine and dispersive, consistent with the AC-STEM results.

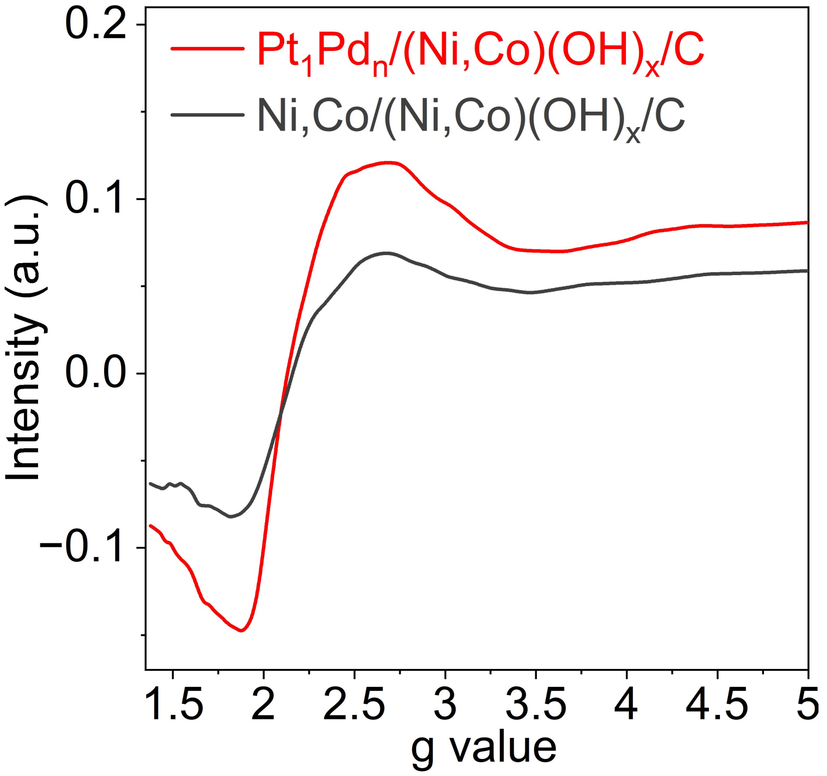

Figure S4. Electron paramagnetic resonance (EPR) spectra for the $Pt_1Pd_n/(Ni,Co)(OH)_x/C$ and $NiCo/(Ni,Co)(OH)_x/C$ samples.

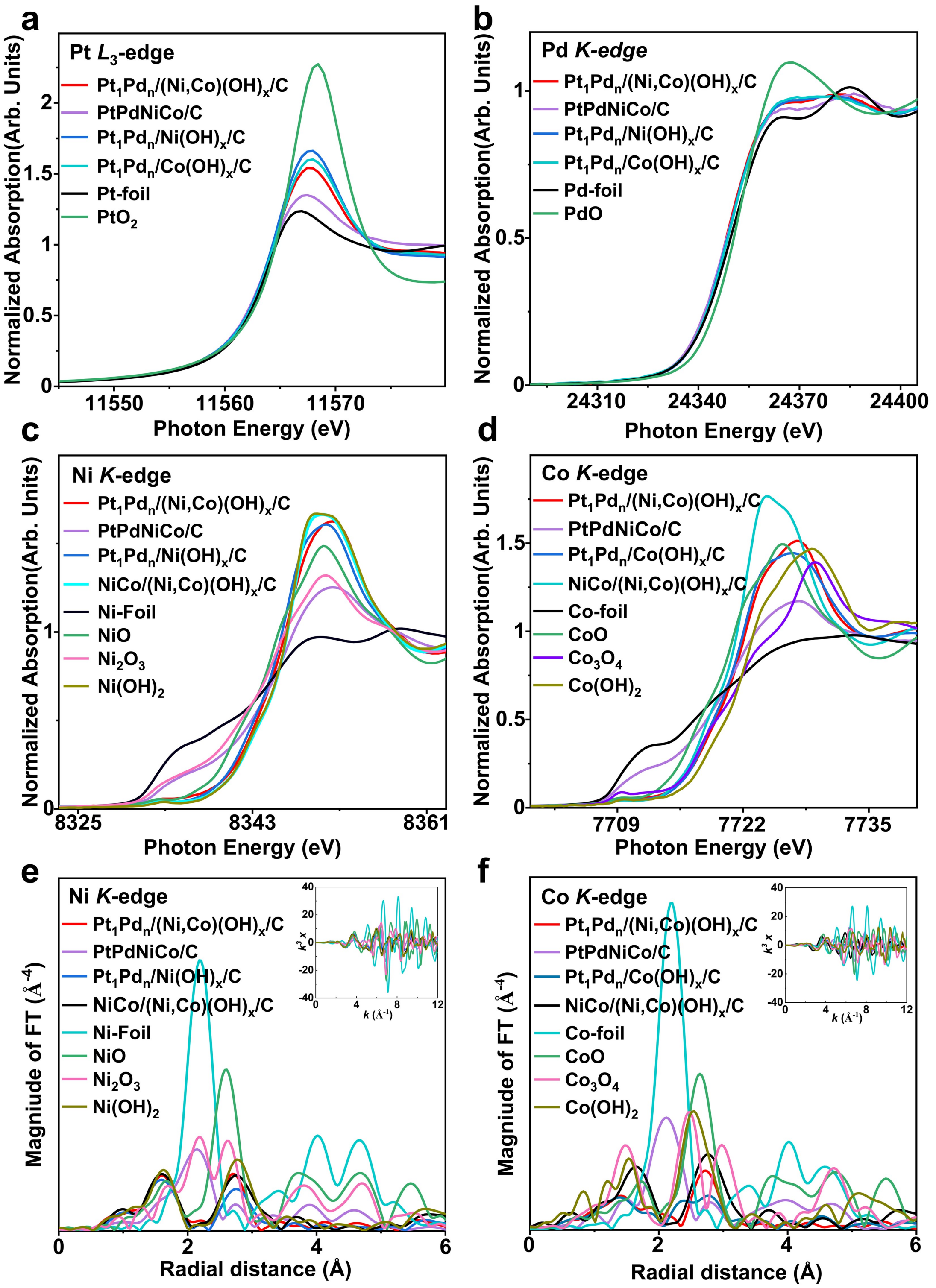

Figure S5. XAS analyses of the as-obtained catalysts. XANES and Fourier transformed EXAFS spectra for the $Pt_1Pd_n/(Ni,Co)(OH)_x/C$, $NiCo/(Ni,Co)(OH)_x/C$, $Pt_1Pd_n/Ni(OH)_x/C$, $Pt_1Pd_n/Co(OH)_x/C$, and PtPdNiCo/C catalysts recorded at **a** Pt L_3-edge, **b** Pd *K*-edge, **c, e** Ni *K*-edge and its k^3-weight EXAFS (inset), **d, f** Co *K*-edge and its k^3-weight EXAFS (inset). The spectra of the reference compound Ni-foil, NiO, Ni_2O_3, $Ni(OH)_2$, Co-foil, CoO, Co_3O_4, $Co(OH)_2$, Pt-foil, PtO_2, Pd-foil, and PdO are also shown for comparison.

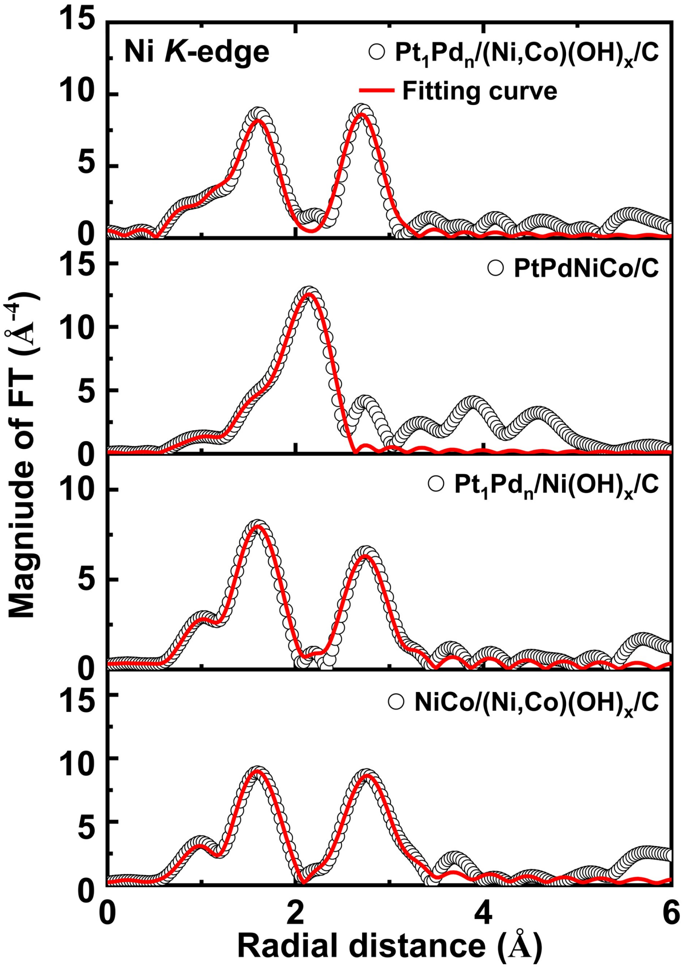

Figure S6. Fourier-transformed EXAFS data (symbol) and the best-fit results (red line) of Ni *K*-edge. Data at Ni *K*-edge for $Pt_1Pd_n/(Ni,Co)(OH)_x/C$, PtPdNiCo/C, $Pt_1Pd_n/Ni(OH)_x/C$ and $NiCo/(Ni,Co)(OH)_x/C$.

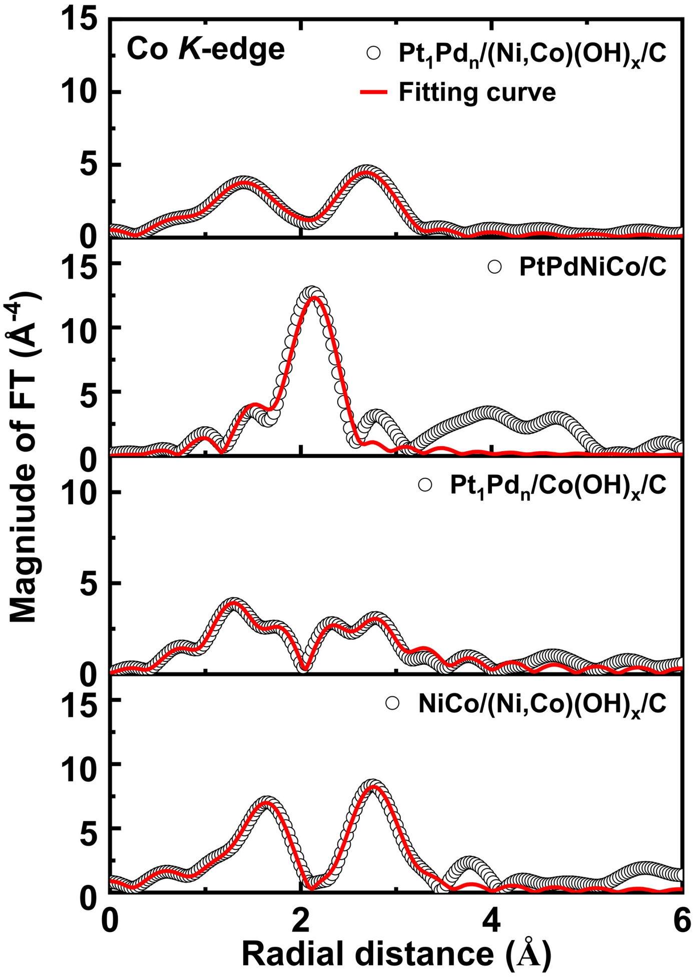

Figure S7. Fourier-transformed EXAFS data (symbol) and the best-fit results (red line) of Co *K*-edge. Data at Co *K*-edge for $Pt_1Pd_n/(Ni,Co)(OH)_x/C$, PtPdNiCo/C, $Pt_1Pd_n/Co(OH)_x/C$ and $NiCo/(Ni,Co)(OH)_x/C$.

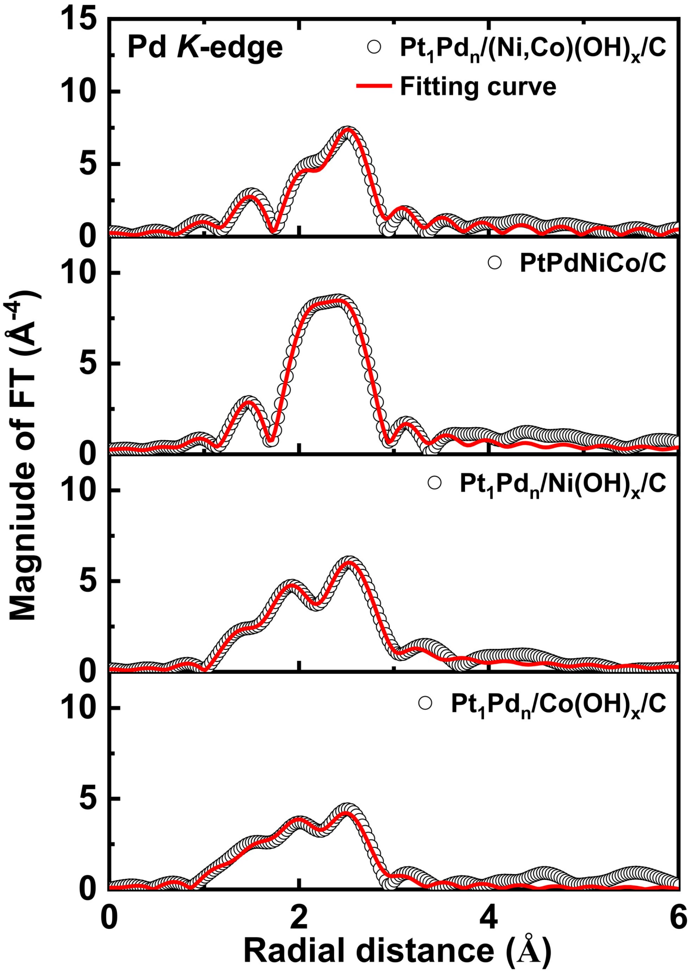

Figure S8. Fourier-transformed EXAFS data (symbol) and the best-fit results (red line) of Pd *K*-edge. Data at Pd *K*-edge for $Pt_1Pd_n/(Ni,Co)(OH)_x/C$, PtPdNiCo/C, $Pt_1Pd_n/Ni(OH)_x/C$ and $Pt_1Pd_n/Co(OH)_x/C$.

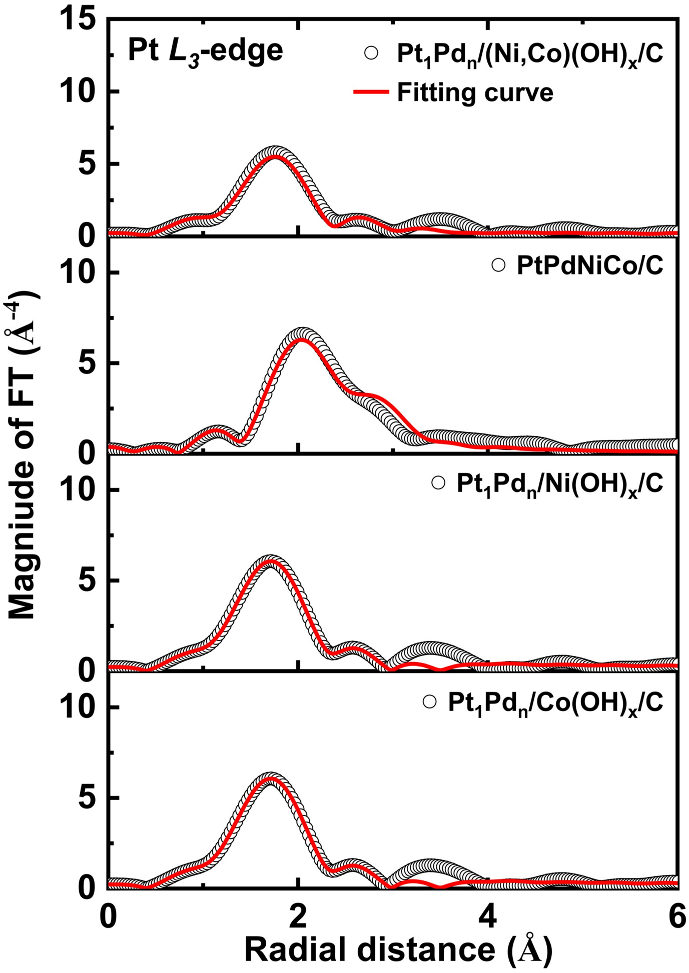

Figure S9. Fourier-transformed EXAFS data (symbol) and the best-fit results (red line) Pt L_3-edge.

Data at Pt L_3-edge for $Pt_1Pd_n/(Ni,Co)(OH)_x/C$, PtPdNiCo/C, $Pt_1Pd_n/Ni(OH)_x/C$ and $Pt_1Pd_n/Co(OH)_x/C$.

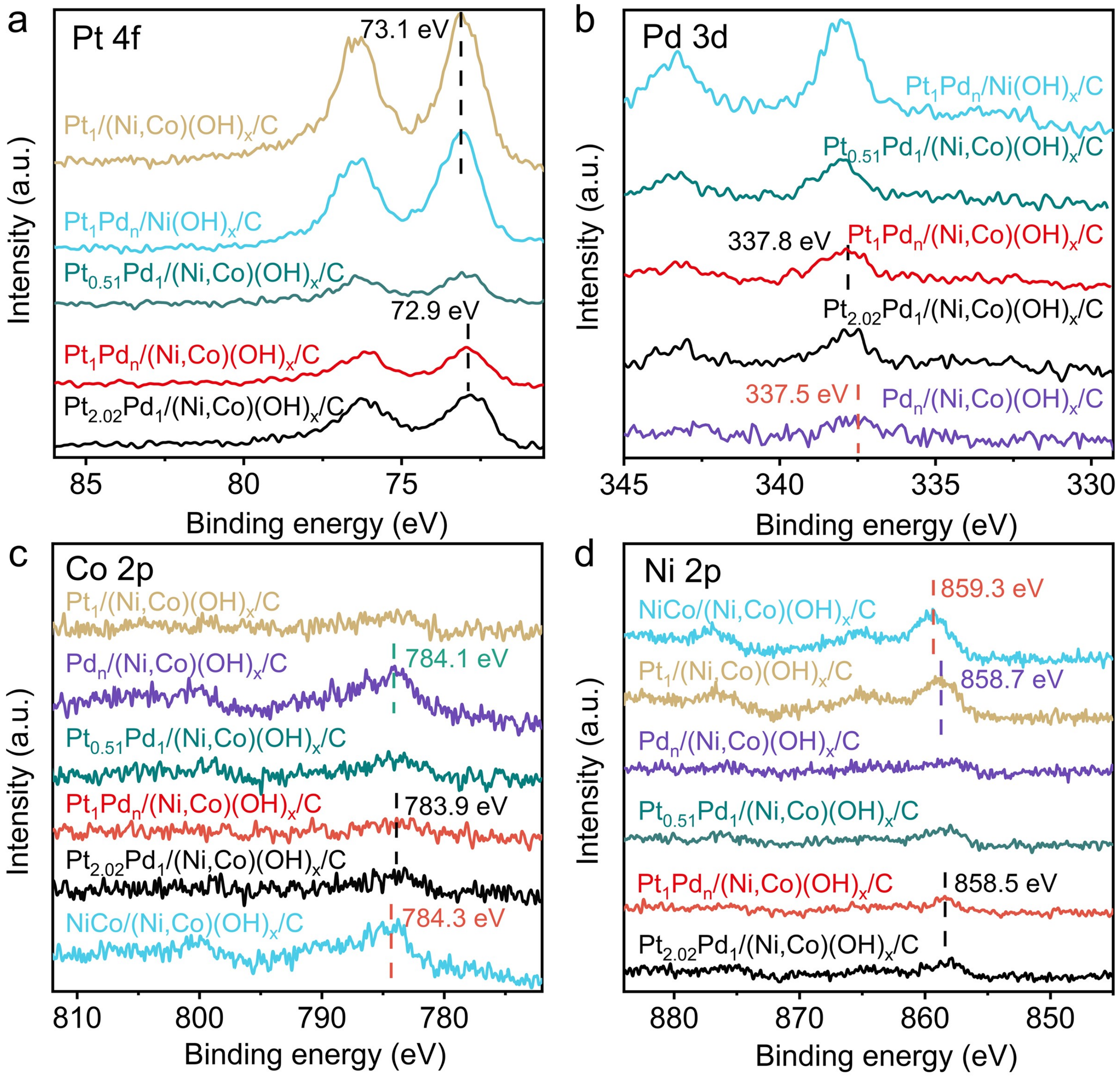

Figure S10. XPS spectra characterizations. (A) Pt 4f, (B) Pd 3d, (C) Co 2p, (D) Ni 2p for the as-prepared catalysts.

366

367

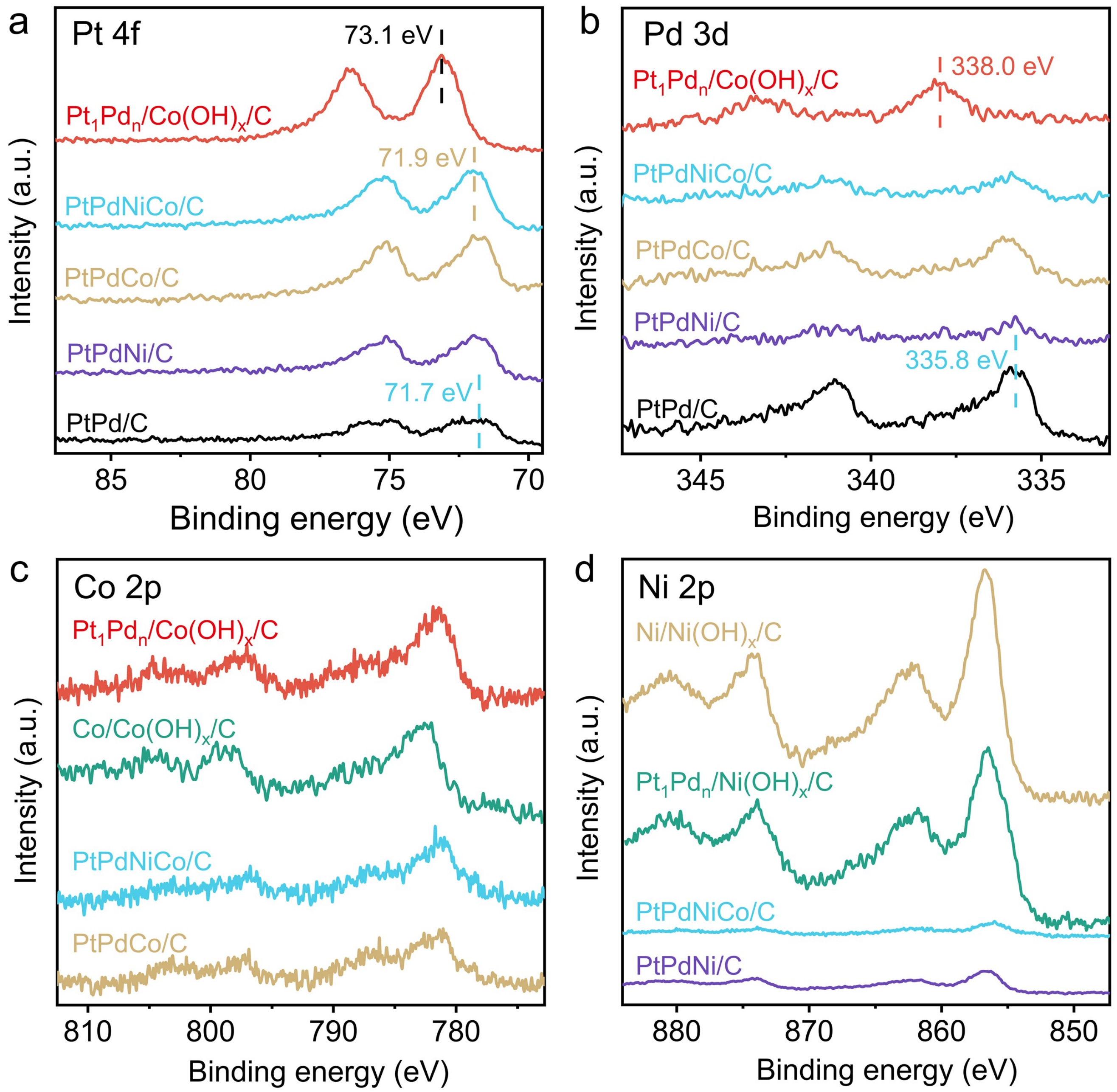

368

369 **Figure S11. XPS spectra for the as-prepared catalysts.** (A) Pt 4f, (B) Pd 3d, (C) Co 2p, (D) Ni 2p.

370

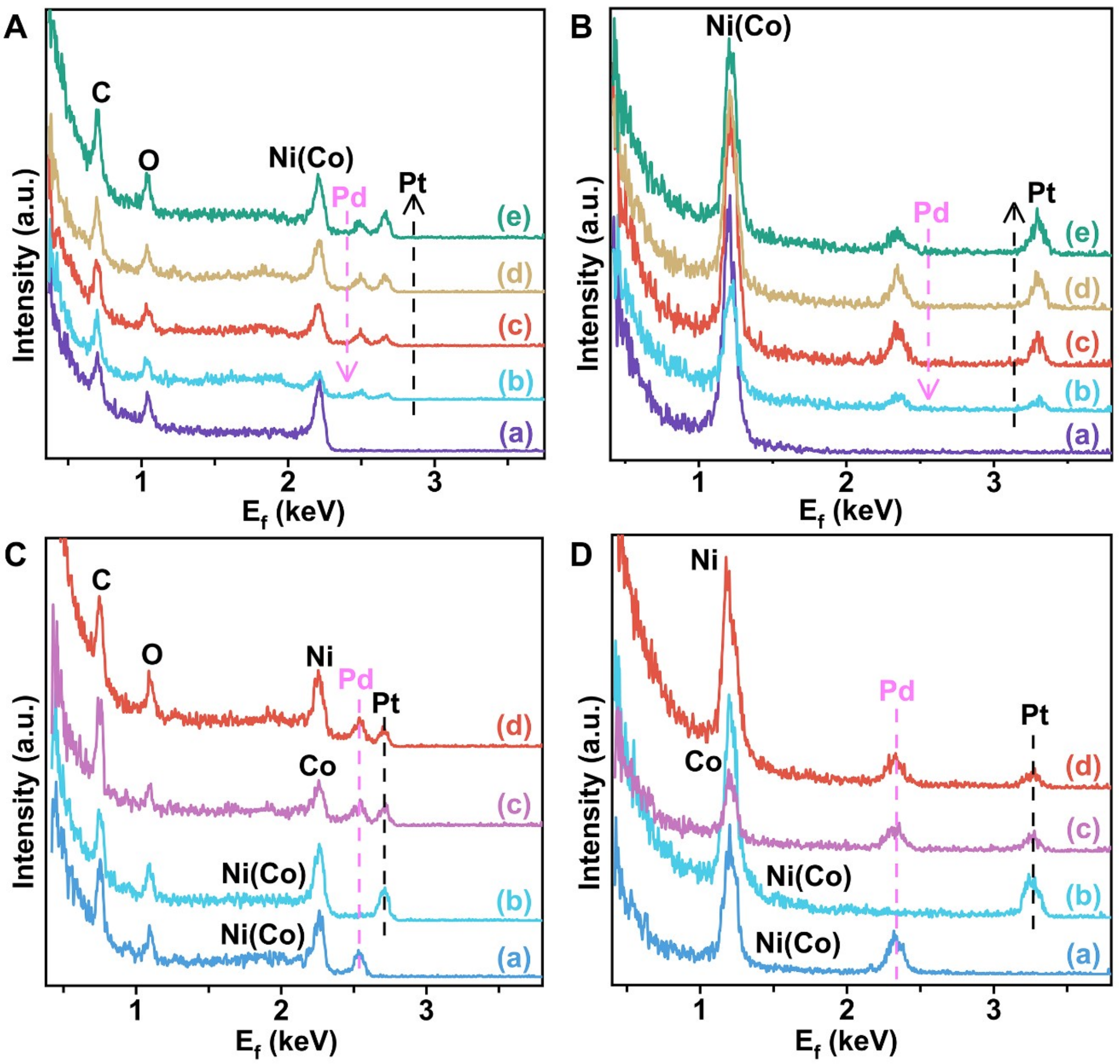

Figure S12. HS-LEIS spectra for the as-prepared catalysts. (A) 3 keV $^{4}He^{+}$ and (B) 5 keV $^{20}Ne^{+}$ HS-LEIS spectra for (a) NiCo/(Ni,Co)$(OH)_x$/C, (b) PtPdNiCo/C, (c) $Pt_{0.51}Pd_1$/(Ni,Co)$(OH)_x$/C, (d) Pt_1Pd_n/(Ni,Co)$(OH)_x$/C, (e) $Pt_{2.02}Pd_1$/(Ni,Co)$(OH)_x$/C, (C) 3 keV $^{4}He^{+}$ and (D) 5 keV $^{20}Ne^{+}$ HS-LEIS spectra for (a) Pd_n/(Ni,Co)$(OH)_x$/C, (b) Pt_1/(Ni,Co)$(OH)_x$/C, (c) Pt_1Pd_n/Co$(OH)_x$/C, (d) Pt_1Pd_n/Ni$(OH)_x$/C.

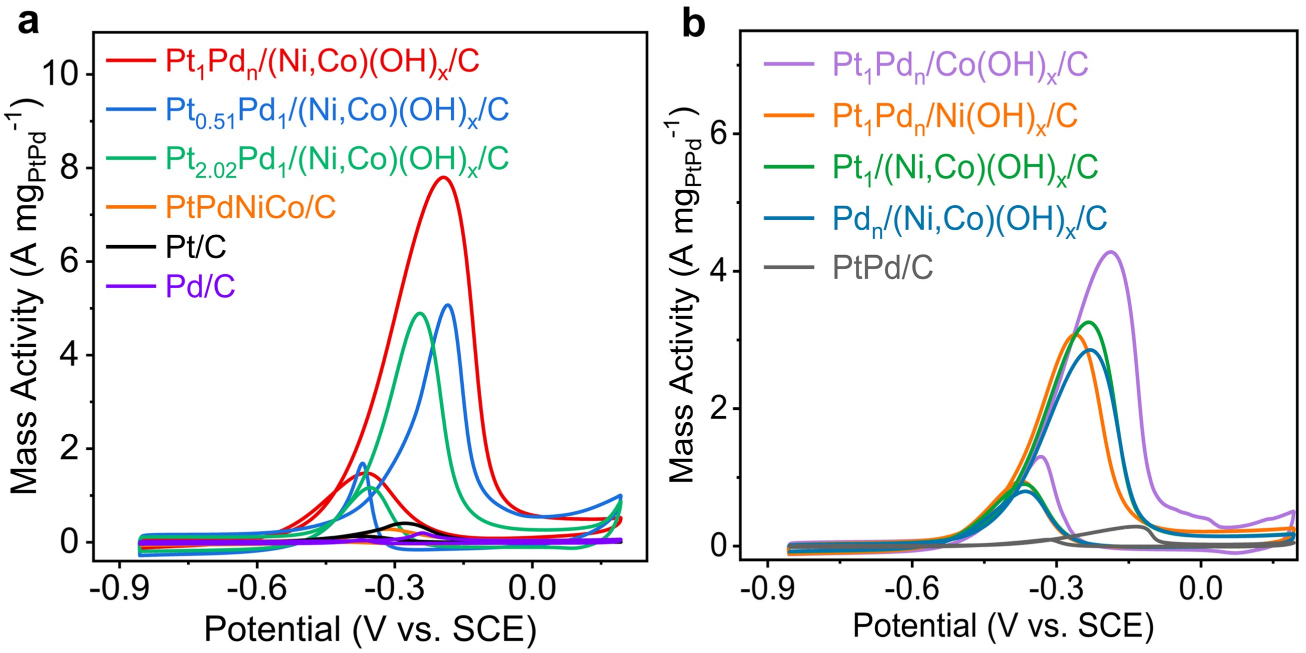

Figure S13. PtPd loading-normalized cyclic voltammograms of the as-prepared catalysts. (a) $Pt_1Pd_n/(Ni,Co)(OH)_x/C$, $Pt_{0.51}Pd_1/(Ni,Co)(OH)_x/C$, $Pt_{2.02}Pd_1/(Ni,Co)(OH)_x/C$, PtPdNiCo/C, Pt/C and Pd/C, (b) $Pt_1Pd_n/Co(OH)_x/C$, $Pt_1Pd_n/Ni(OH)_x/C$, $Pt_1/(Ni,Co)(OH)_x/C$, $Pd_n/Co(OH)_x/C$, and PtPd/C recorded in 1.0 M KOH + 1.0 M CH_3OH solution.

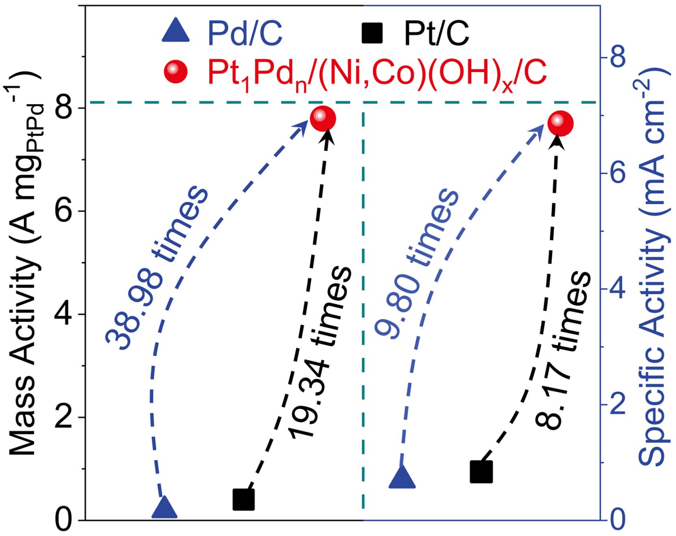

Figure S14. Mass and specific activity of $Pt_1Pd_n/(Ni,Co)(OH)_x/C$, Pt/C, and Pd/C for MOR.

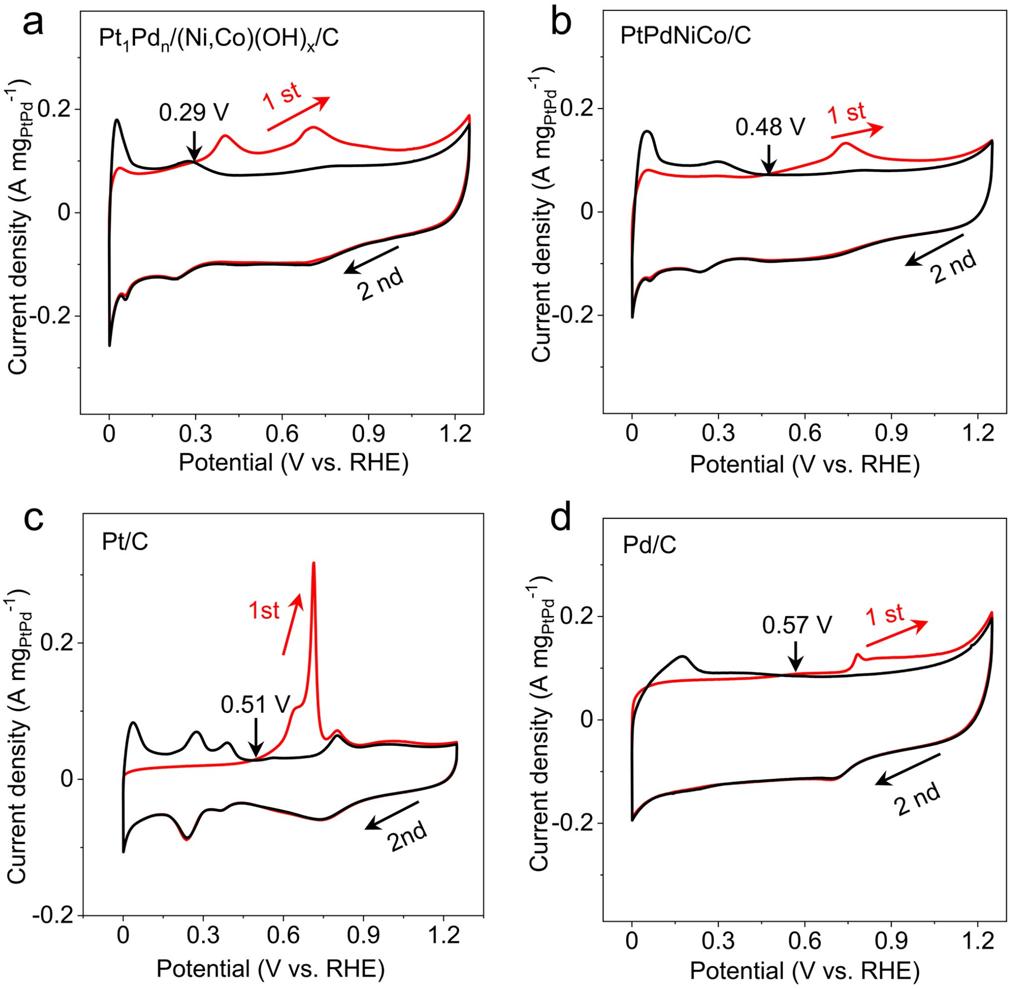

Figure S15. CO-stripping experiments of (a) $Pt_1Pd_n/(Ni,Co)(OH)_x/C$, (b) PtPdNiCo/C, (c) Pt/C and (d) Pd/C.

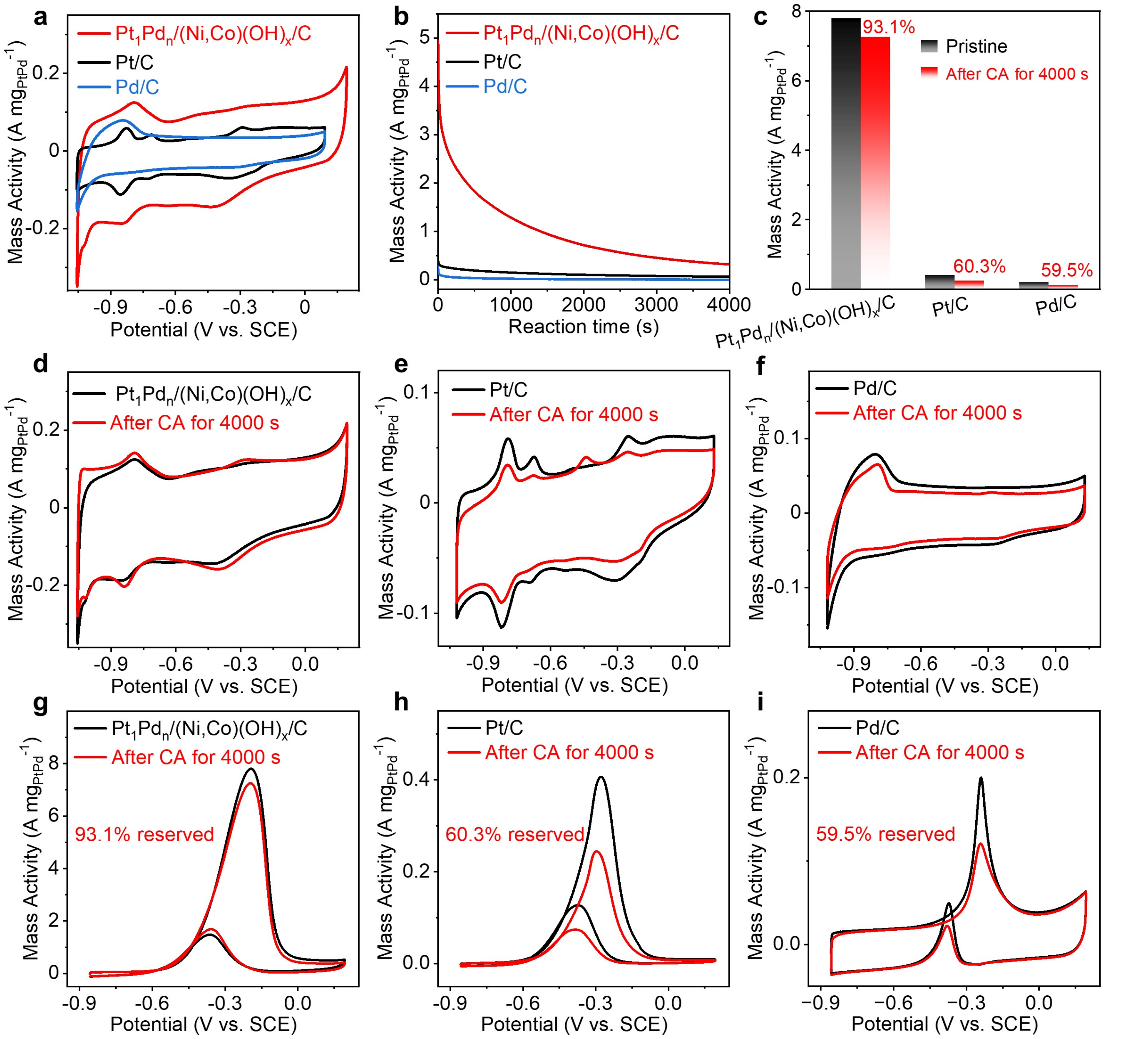

Figure S16. Comparison of MOR stability of different catalysts. (a) The comparison towards the $Pt_1Pd_n/(Ni,Co)(OH)_x/C$, Pt/C, and Pd/C catalysts was measured in 1.0 M KOH solution. (b) The chronoamperometry recorded curves of $Pt_1Pd_n/(Ni,Co)(OH)_x/C$, Pt/C, and Pd/C catalysts. (c) the initial and remained mass activity for the $Pt_1Pd_n/(Ni,Co)(OH)_x/C$, Pt/C, and Pd/C catalysts. The initial CV curves and the CV curves after chronoamperometry for 4000 s of the (d) $Pt_1Pd_n/(Ni,Co)(OH)_x/C$, (e) Pt/C, and (f) Pd/C catalysts recorded in 1.0 M KOH solution, and 1.0 M KOH +1.0 M CH_3OH solution.

Note: As shown in Figure S16, the measurements of PtPd loading-normalized cyclic voltammograms of the initial samples and after chronoamperometry for 4000 s were conducted in 1.0 M KOH with/without 1.0 M CH_3OH solution, respectively. $Pt_1Pd_n/(Ni,Co)(OH)_x/C$ displayed much larger double-layer capacitance than PtPdNiCo/C and Pt/C, suggesting its excellent electrochemical performance. Noteworthy, the retained activity of $Pt_1Pd_n/(Ni,Co)(OH)_x/C$ retained up to 93.1%. It indicates that the Pt single atoms, Pd clusters anchoring on $(Ni,Co)(OH)_x$ nanoparticles are crucially important to the MOR stability in the long-term test. The commercial Pt/C and Pd/C exhibited the worst MOR stability, their mass activity was decreased to 60.3% and 59.5%, respectively after 4000 s probably due to the nanoparticle agglomeration or the Pt and Pd sites being poisoned by CO* intermediate. It declares that it is an effective way to improve MOR stability by introducing transition metal or transition metal hydroxide to cooperate with Pt sites.

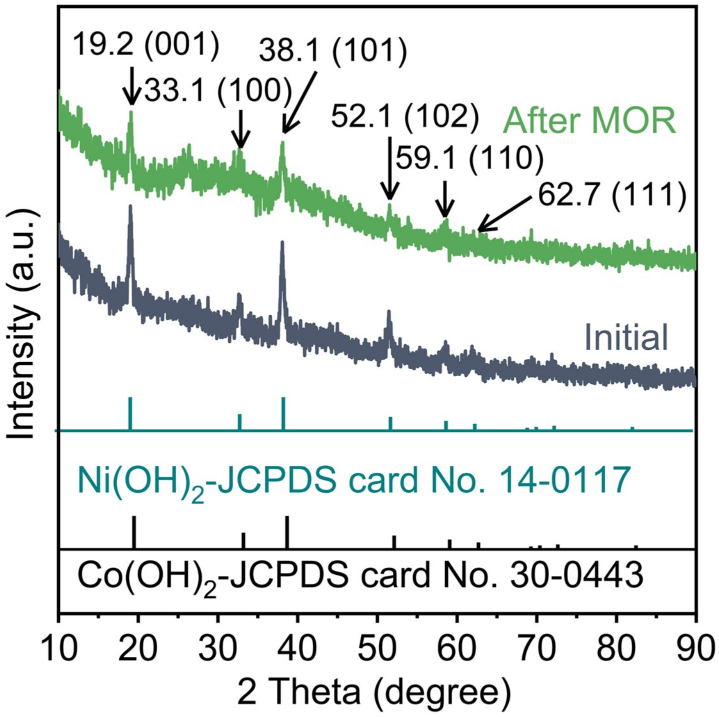

Figure S17. XRD patterns for the initial $Pt_1Pd_n/(Ni,Co)(OH)_x/C$ sample and reactivated after the MOR chronoamperometry for 4000 s.

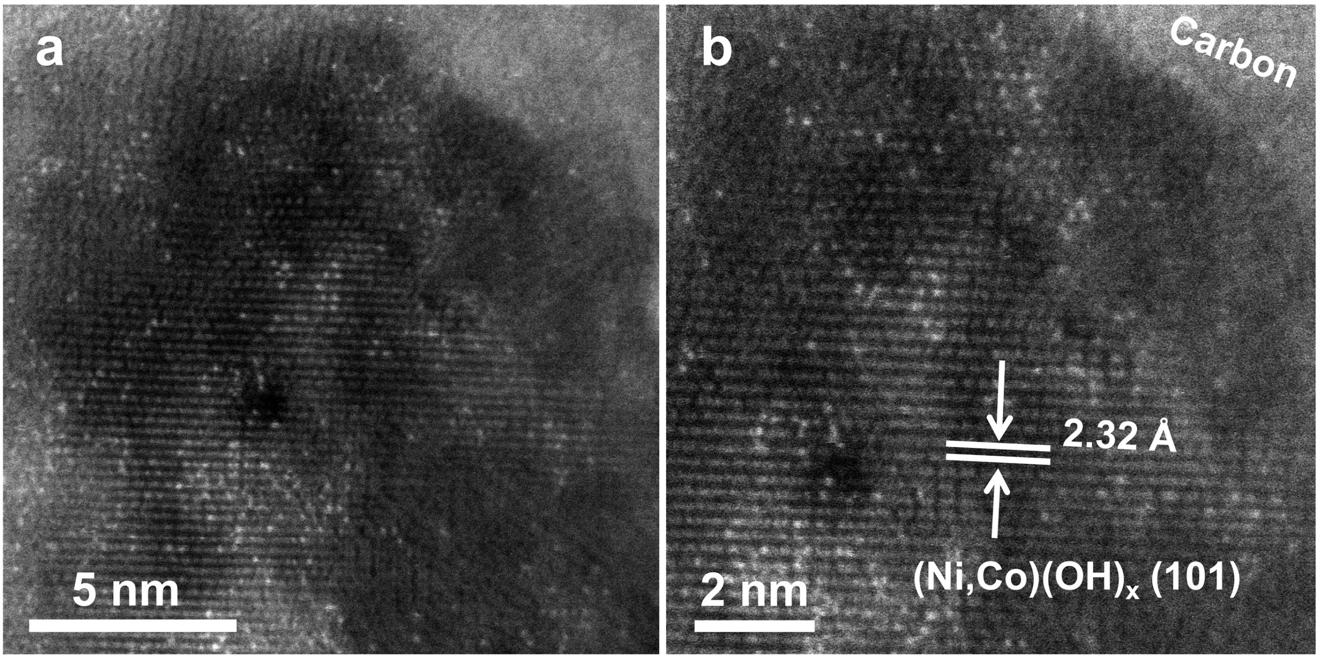

Figure S18. AC-STEM images of the $Pt_1Pd_n/(Ni,Co)(OH)_x/C$ sample after the MOR chronoamperometry for 4000 s, Scale bars: (a) 5 nm. (b) 2 nm.

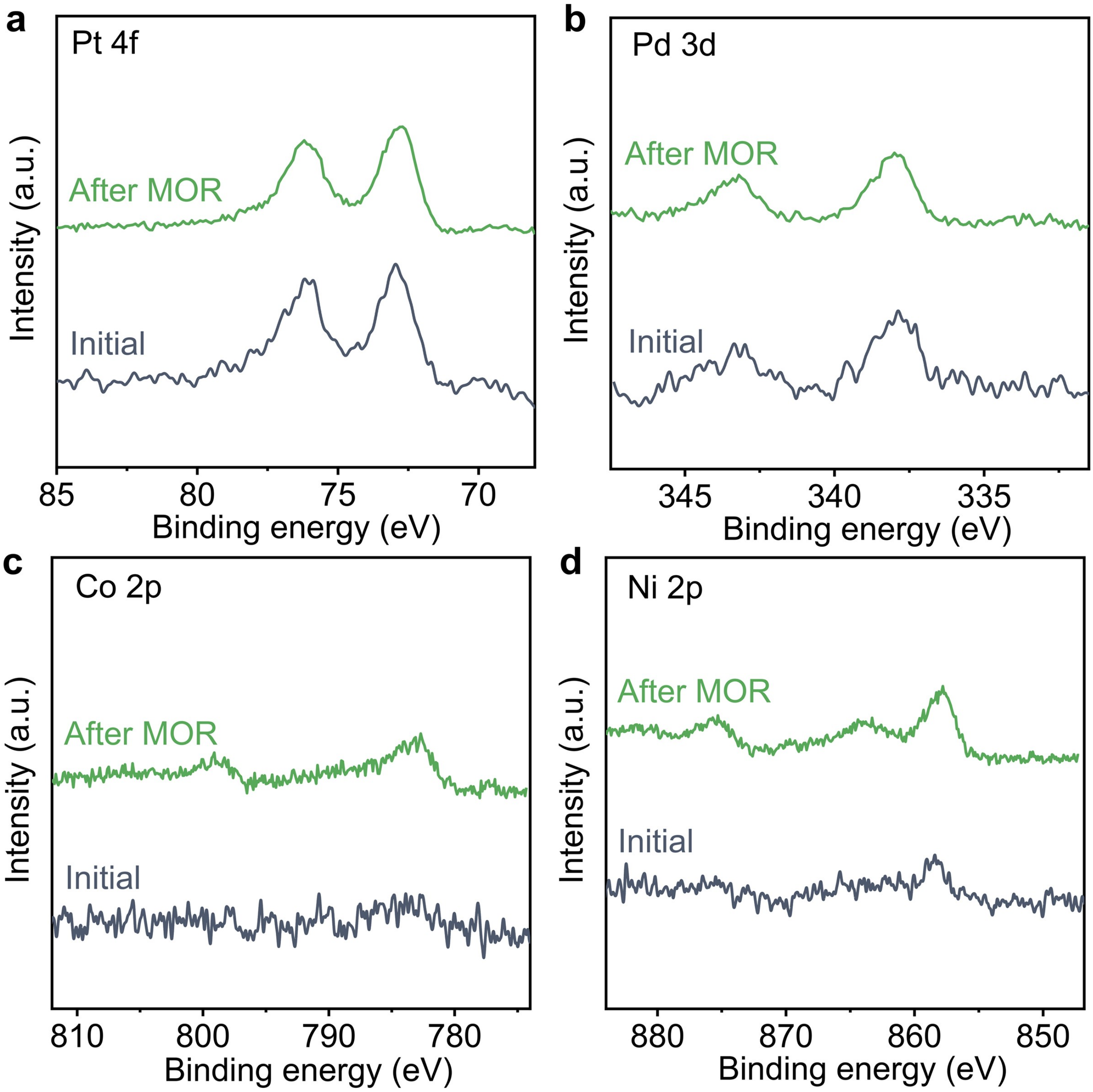

Figure S19. XPS spectra of initial $Pt_1Pd_n/(Ni,Co)(OH)_x/C$ sample and after the MOR chronoamperometry for 4000 s.

Note: The binding energies with Pt 4f and Pd 3d, as noted from the XPS, remain unchanged. Hence, the coordinated and unsaturated Pt and Pd sites can moderately interact with $(Ni,Co)(OH)_x$ to enhance the MOR stability. Gradually dehydrogenation of methanol molecules occurred and the OH^- in the $Pt_1Pd_n/(Ni,Co)(OH)_x/C$ catalyst directly stabilized the reactant intermediates and participated in the

catalyst reconstruction during the MOR cycle. The enhanced signals of Co 2p and Ni 2p in the XPS may be due to the full activation of the surface area of the catalyst after the electrochemical stability test (Figure S19).

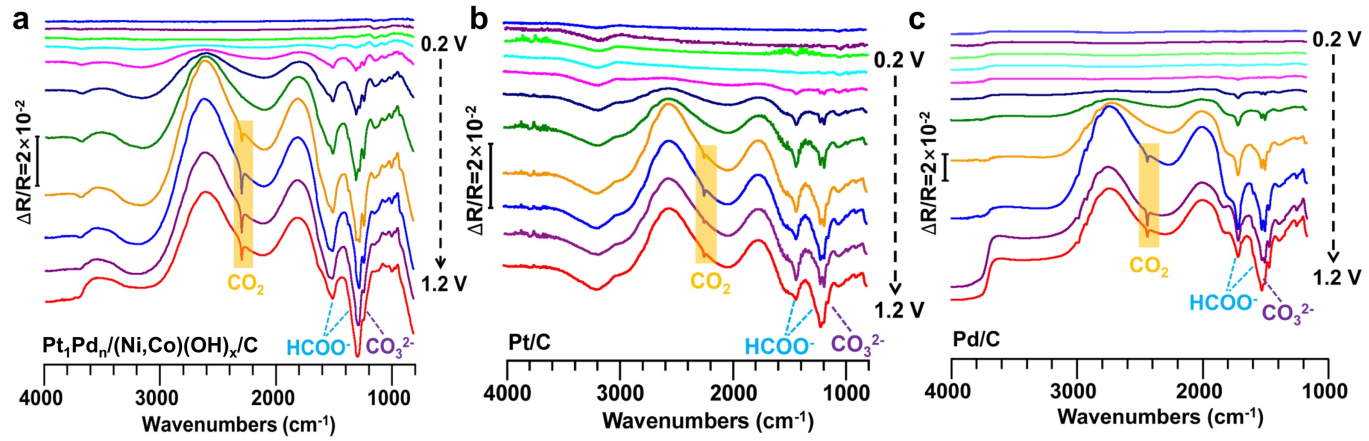

Figure S20. *In-situ* FTIR spectra of $Pt_1Pd_n/(Ni,Co)(OH)_x/C$, Pt/C, and Pd/C (the range of the test was between 0.200 V and 1.200 V versus RHE, and each step was 0.100 V).

Note: In this work, electrochemical *in-situ* infrared spectroscopy was conducted for further study towards the generation and adsorption strength of intermediates (e.g., CO, HCOOH, etc.) during the real-time MOR process in alkaline conditions, as shown in Figure S20. The FTIR spectra of $Pt_1Pd_n/(Ni,Co)(OH)_x/C$ (Figure S20) display four main vibration bands. 2344 cm^{-1} is assigned to carbon dioxide (CO_2) vibration bands, and the wide vibration bands of CO_3^{2-} is 1376 cm^{-1} [39-41]. Moreover, the asymmetric stretching vibration bands of $HCOO^-$ are attributed to 1585 cm^{-1}, while the typical symmetric vibration twin bands of the peaks are at 1381 cm^{-1} and 1348 cm^{-1} [42-44].

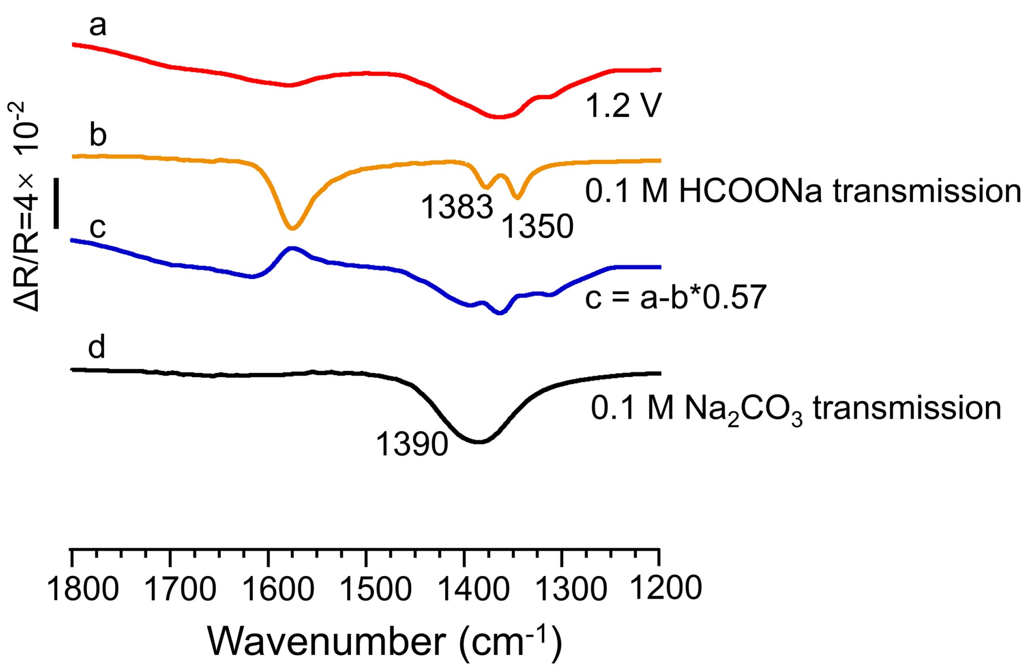

Figure S21. Illustration of the subtracting procedure of IR spectra. (a) the *in-situ* FTIR spectrum of methanol electro-oxidation of $Pt_1Pd_n/(Ni,Co)(OH)_x/C$ at 1.200 V (versus RHE); (b) transmission IR spectrum of 0.1 M HCOONa and as subtrahend; (c) the resulted subtraction spectrum; (d) transmission IR spectrum of 0.1 M Na_2CO_3.

Note: Additionally, the soluble product distributions of methanol oxidation were quantitative analytical for *in-situ* FTIR study[46-48]. The FTIR spectra of the standard solution were collected and subtracted (Figure S21). Noteworthily, the content of $HCOO^-$ towards $Pt_1Pd_n/(Ni,Co)(OH)_x/C$ is significantly declined after 0.900 V (versus RHE) while Pt/C and Pd/C are gradually weakened after 1.000 V (versus RHE). It confirms that the transformation of $HCOO^-$ intermediate to CO_2 on $Pt_1Pd_n/(Ni,Co)(OH)_x/C$ is more effective than on Pt/C and Pd/C. More importantly, the vibration band of CO_3^{2-} towards $Pt_1Pd_n/(Ni,Co)(OH)_x/C$ emerges at a lower potential (0.400 V versus RHE) than Pt/C (0.500 V versus RHE) and Pd/C (0.600 V versus RHE) (Figure S20, S21).

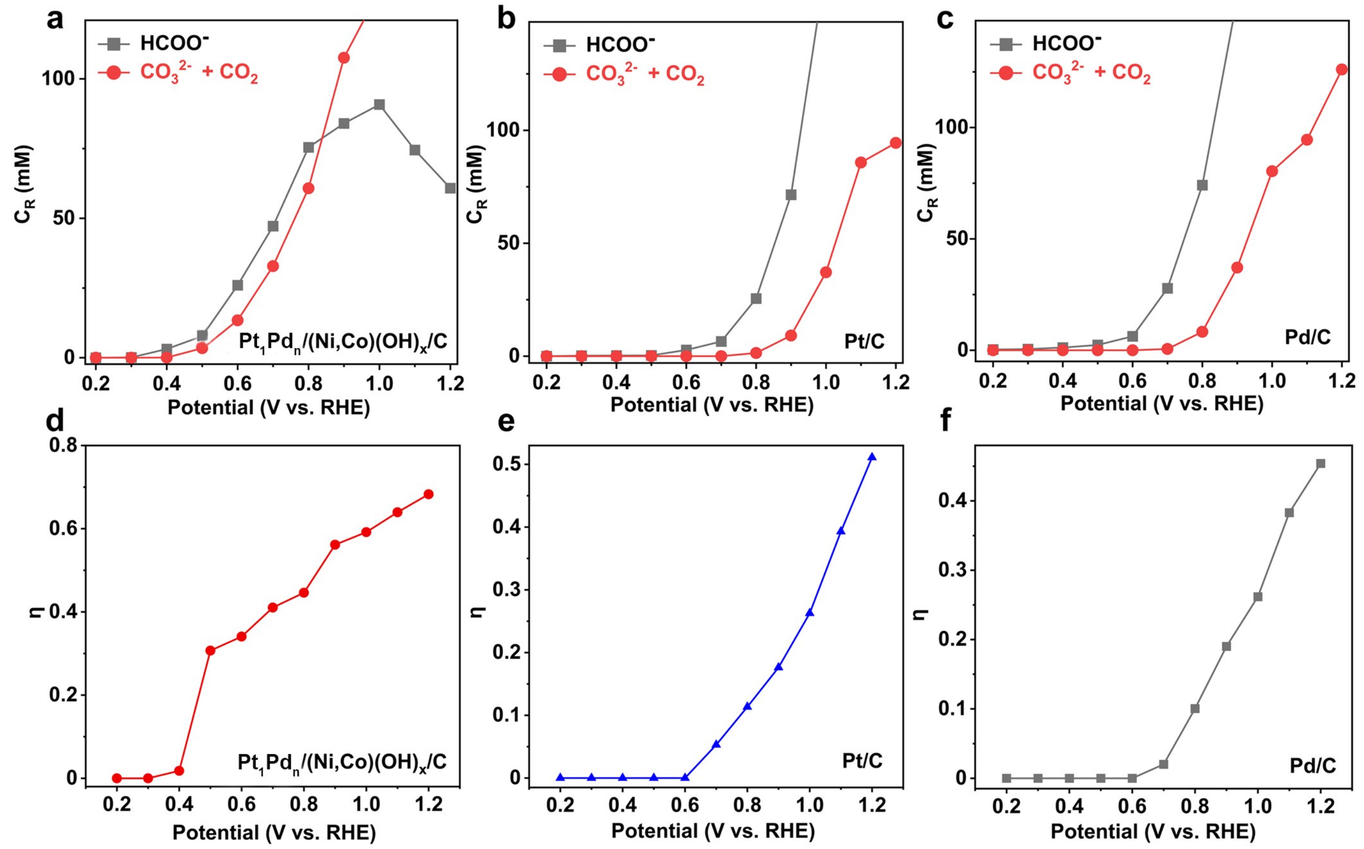

Figure S22. Potential dependence of relative concentration (CR) of $HCOO^-$, CO_3^{2-}, and CO_2 generated from methanol oxidation. (a) $Pt_1Pd_n/(Ni,Co)(OH)_x/C$, (b) Pt/C, and (c) Pd/C. Potential dependence of the selectivity for complete methanol oxidation to CO_2 and CO_3^{2-} (d) $Pt_1Pd_n/(Ni,Co)(OH)_x/C$, (e) Pt/C, and (f) Pd/C electrode.

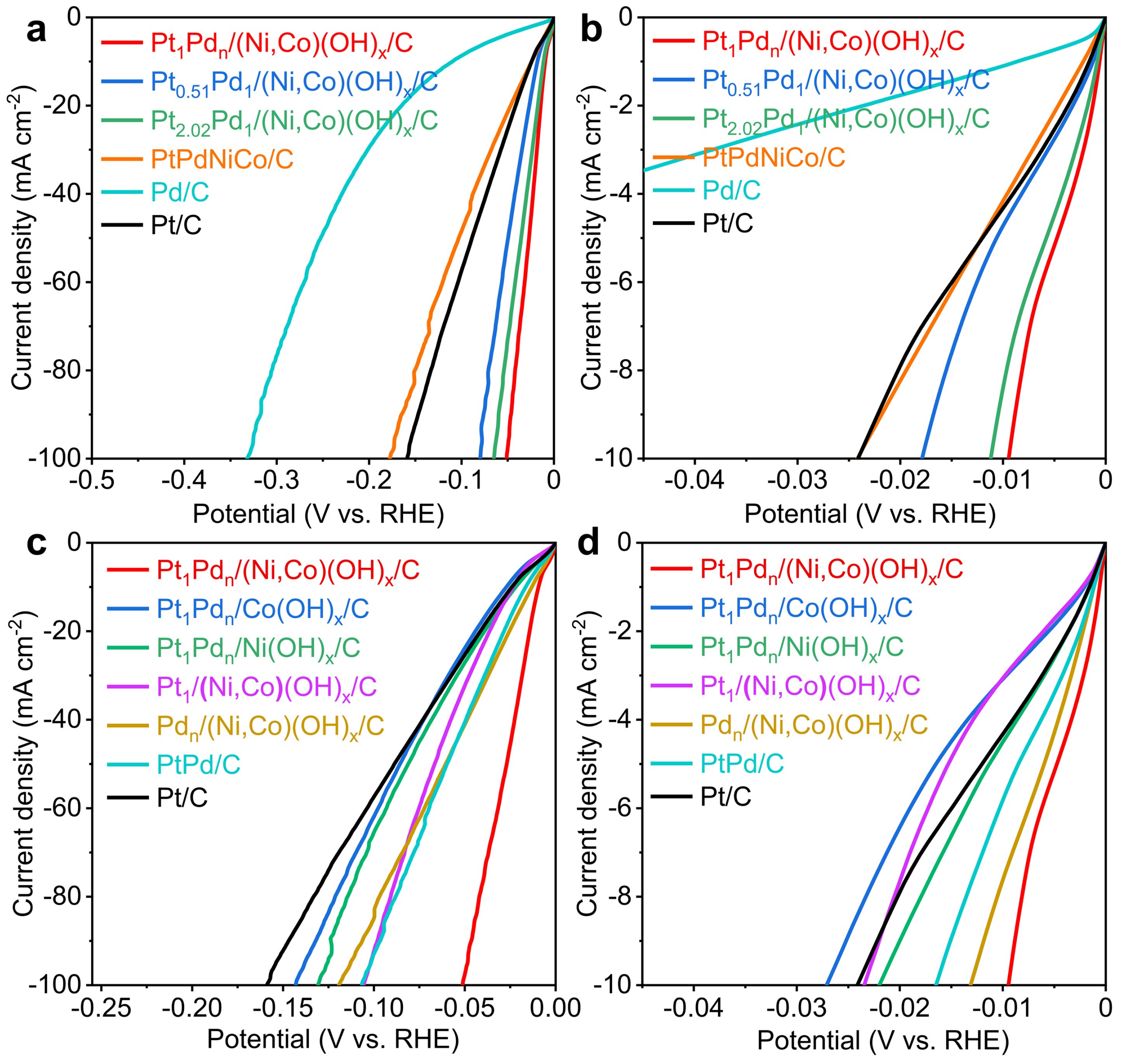

Figure S23. The HER polarization curves of the as-obtained samples in an N_2-saturated 1.0 M KOH solution. Scanning rate, 5 mV s^{-1}.

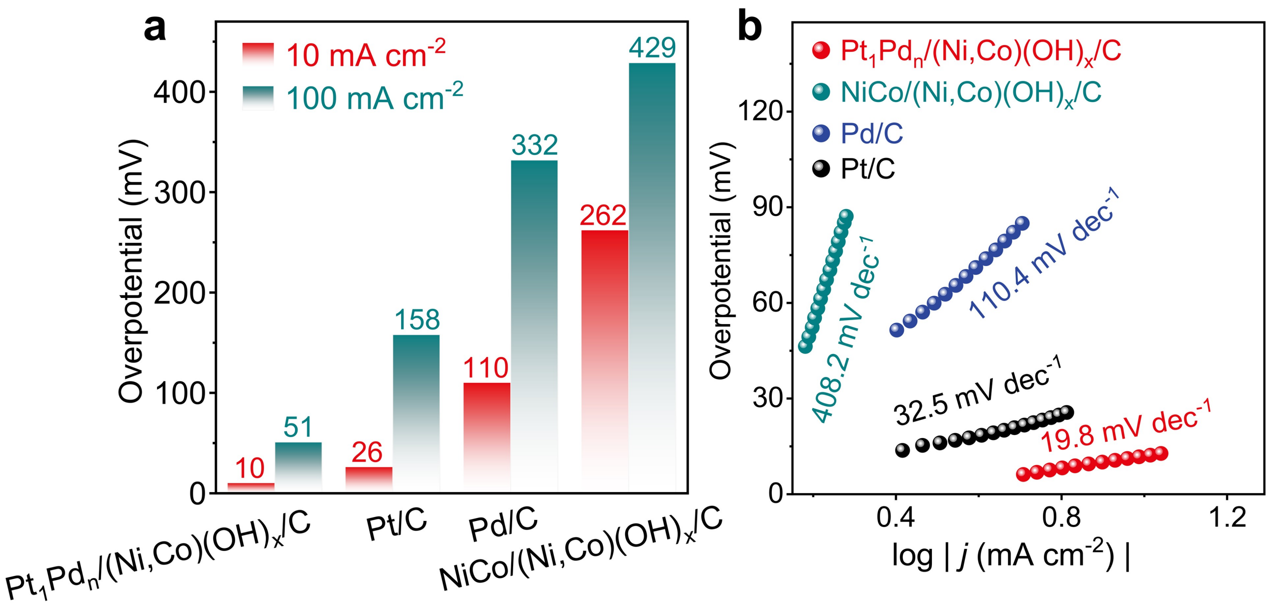

Figure S24. Overpotential and Tafel plots of as-obtained catalysts. (a) Overpotential of as-obtained catalysts at 10 mA cm^{-2} and 100 mA cm^{-2}. (b) Tafel plots of $Pt_1Pd_n/(Ni,Co)(OH)_x/C$, $NiCo/(Ni,Co)(OH)_x/C$, Pd/C and Pt/C.

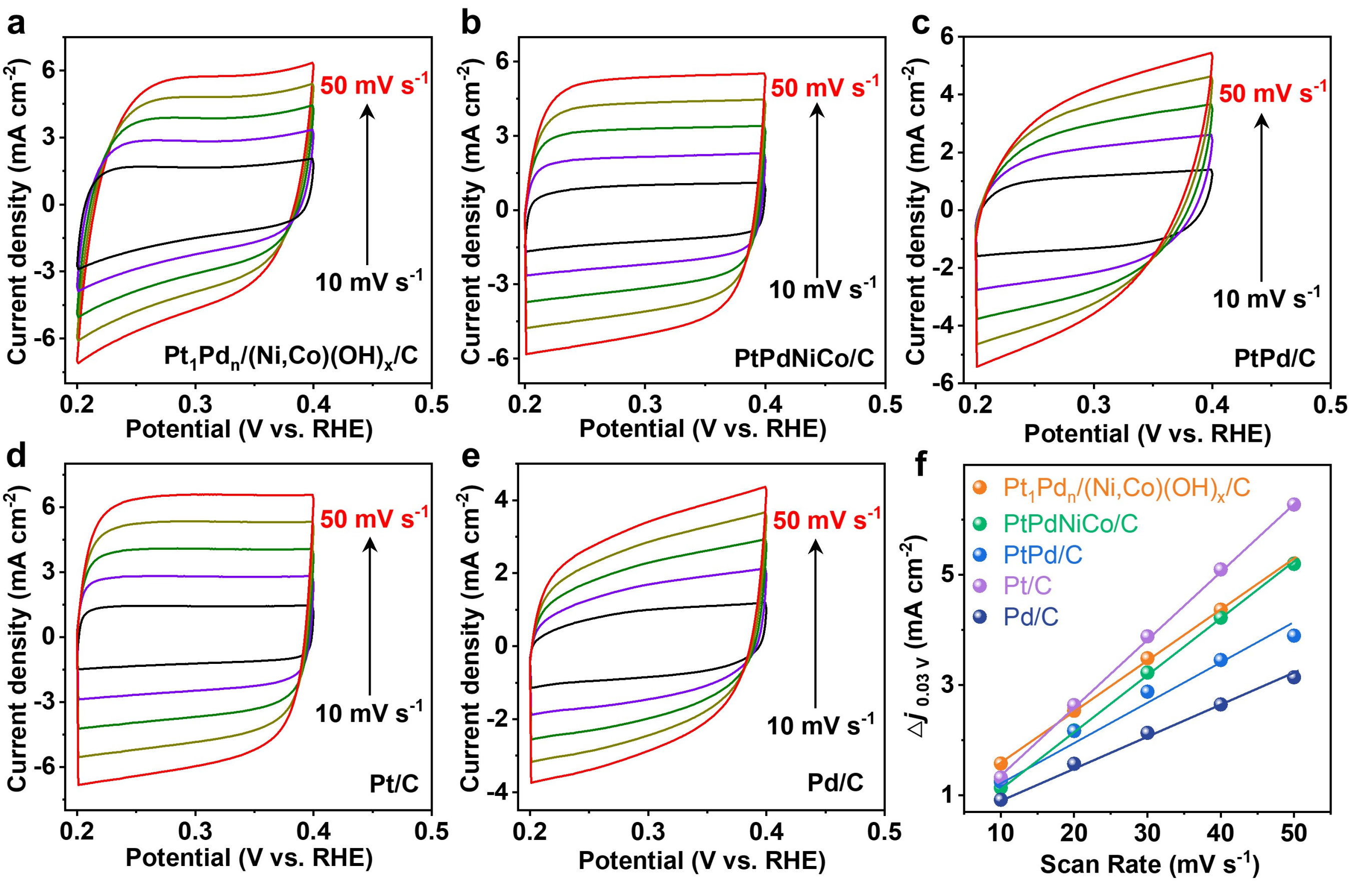

Figure S25. CV curves and linear fitting of the capacitive current versus the CV scanning rate. (a-e) CV curves of the $Pt_1Pd_n/(Ni,Co)(OH)_x/C$, PtPdNiCo/C, PtPd/C, Pt/C, and Pd/C catalysts in the region of 0.200 to 0.400 V (versus RHE) with scanning rate from 10 to 50 mV s^{-1}. (f) Linear fitting of the capacitive current versus the CV scanning rate.

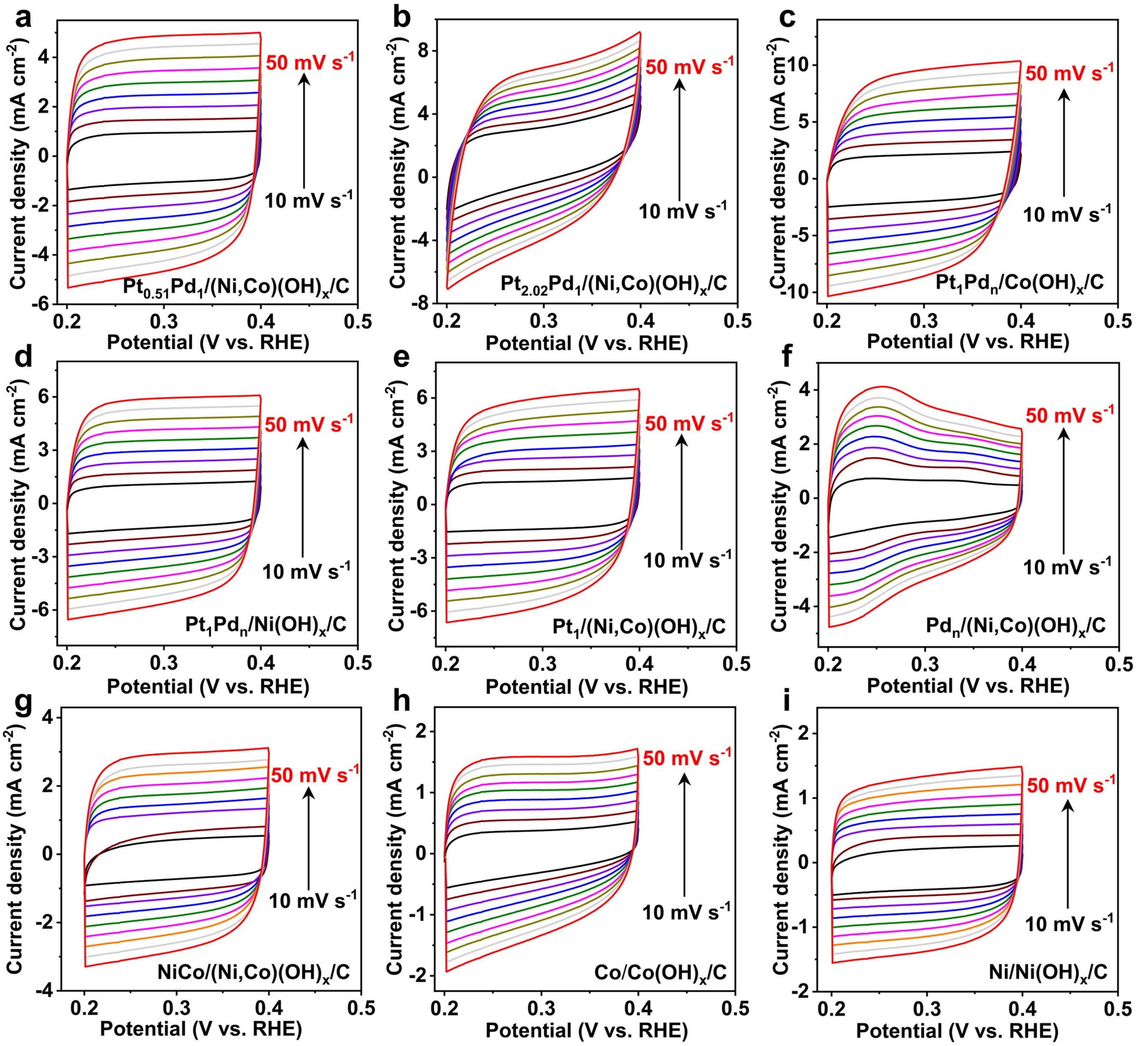

Figure S26. CV curves of the as-obtained catalysts in the region of 0.200 to 0.400 V(versus RHE) with scanning rate from 10 to 50 mV s^{-1} in 1.0 M KOH solution.

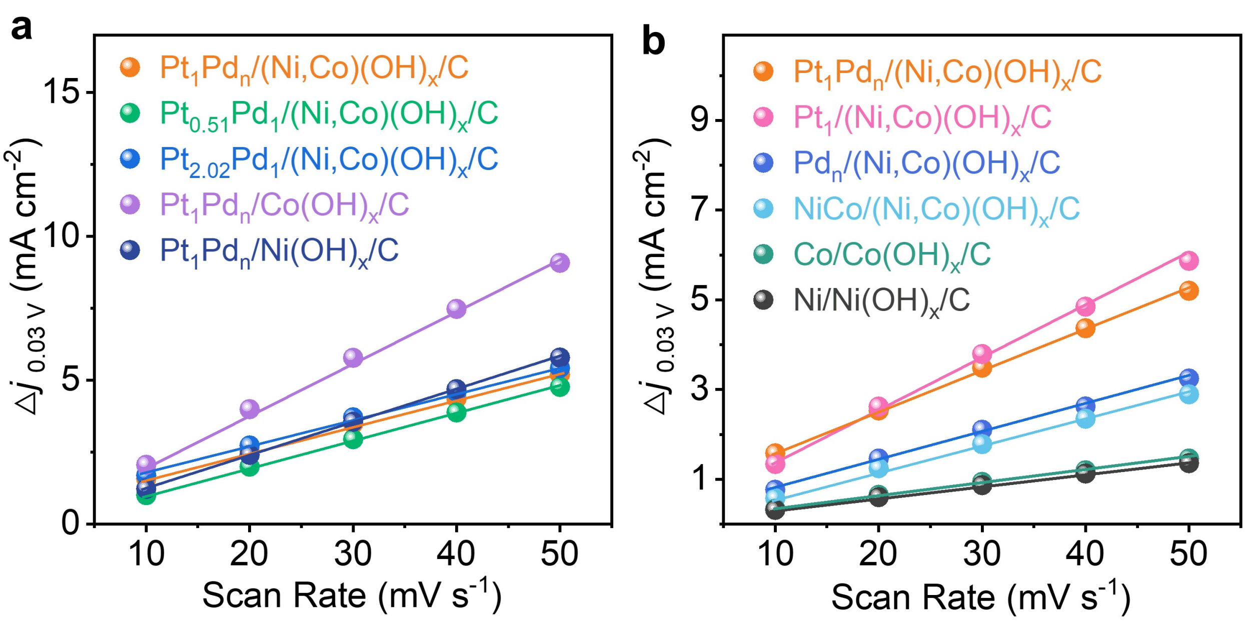

Figure S27. (a, b) Linear fitting of the capacitive current versus the CV scanning rate.

Note: Since the catalyst itself contains carbon black, the contribution of the double-layer capacitance of the carbon black may be relatively large during comparison. Therefore, only a rough comparison is made, intended for reference purposes only.

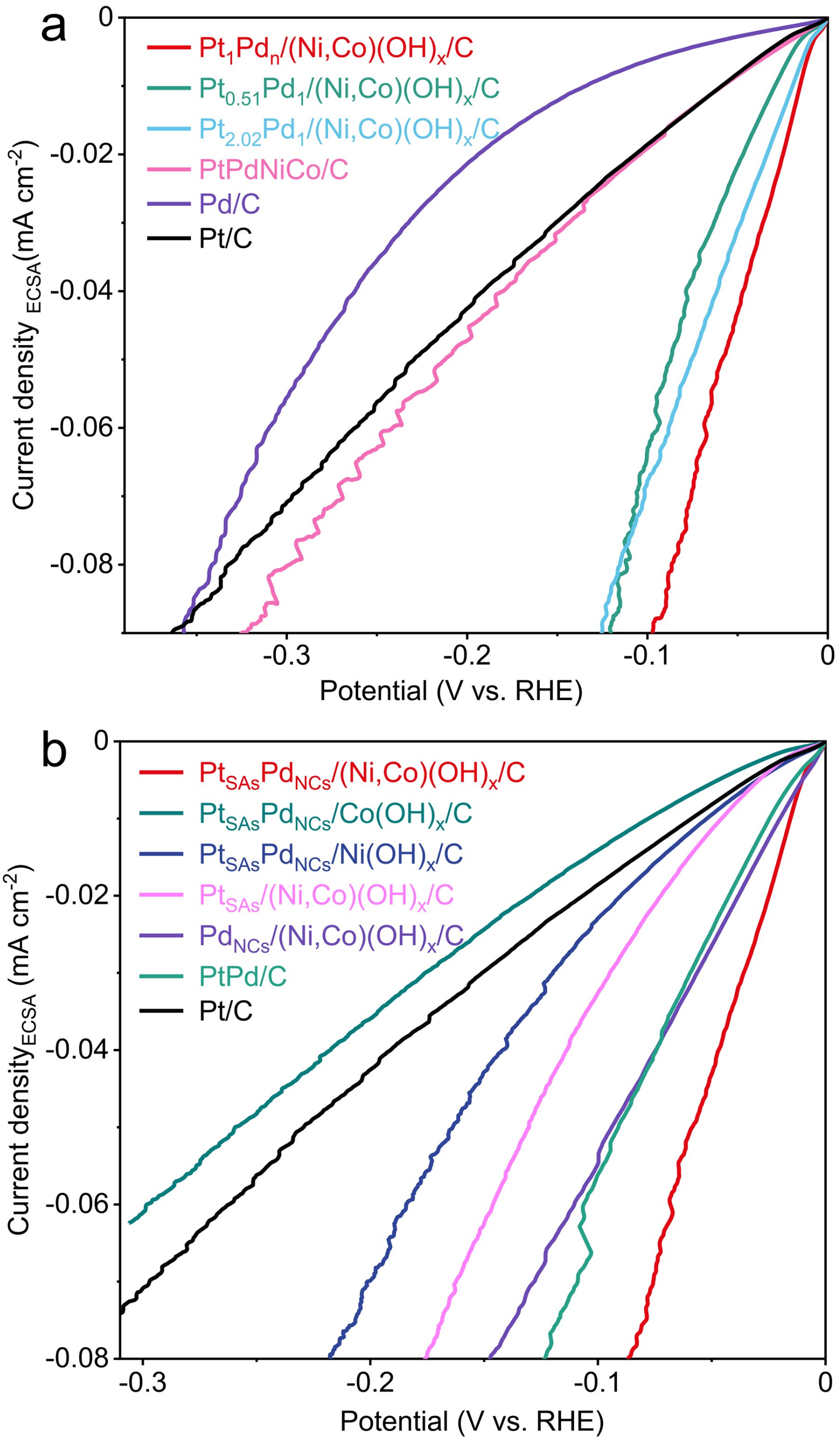

Figure S28. The HER polarization curves normalized by electrochemical active surface areas (ECSAs) of the as-obtained samples in a 1.0 M KOH solution, Scanning rate, 5 mV s^{-1}.

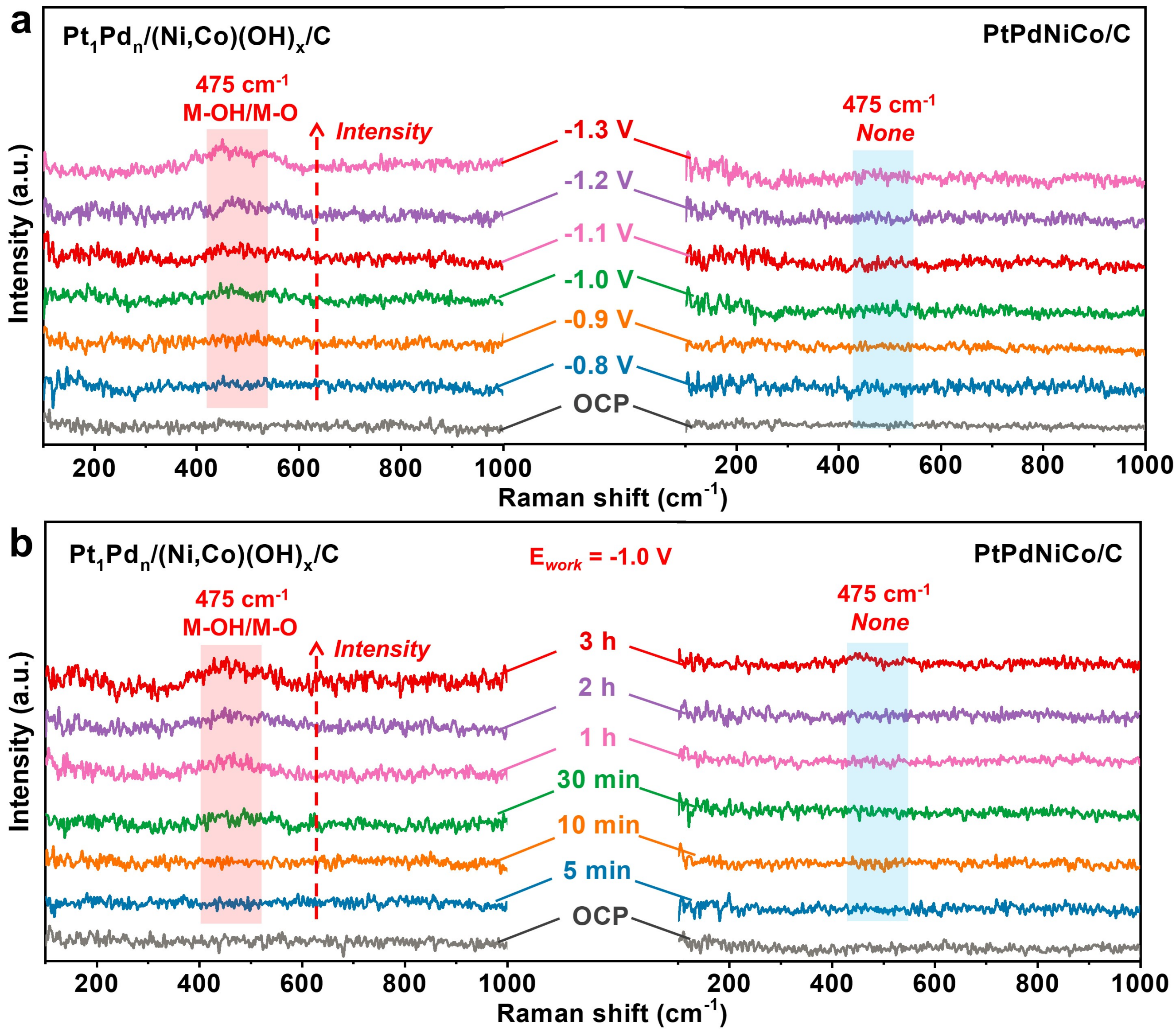

Figure S29. Operando Raman spectra during the chronopotentiometry test at OCP and different applied potential. Each potential was maintained for 1 h towards (a) $Pt_1Pd_n/(Ni,Co)(OH)_x/C$, (c) PtPdNiCo/C. *Operando* Raman spectra during the chronopotentiometry test at -1.000 V (versus Hg/HgO), (b) $Pt_1Pd_n/(Ni,Co)(OH)_x/C$, (d) PtPdNiCo/C in 1.0 M KOH with different time.

Note: Firstly, no obvious peaks appeared except for the signal of carbon substrate when the samples ($Pt_1Pd_n/(Ni,Co)(OH)_x/C$ and PtPdNiCo/C) were soaked in 1.0 M KOH. While the potential is applied to -1.000 V, one broad Raman band in the range of 400 to 600 cm^{-1} is observed for $Pt_1Pd_n/(Ni,Co)(OH)_x/C$, which corresponds to the M-OH and M-O vibration (M = Ni and Co). Besides, this band becomes stronger with the increasing potential, confirming the increment of Ni/Co-OH_{ad} intermediates. A similar Raman band can be hardly detected for PtPdNiCo/C, which is probably associated with the poor

capability for water dissociation. This fact demonstrates that the introduction of Ni(Co) hydroxide triggers the dissociation of water to facilitate the Volmer step of HER. As for the *Operando* Raman during the chronopotentiometry test at -1.000 V (versus Hg/HgO) in 1.0 M KOH towards $Pt_1Pd_n/(Ni,Co)(OH)_x/C$ for 3 h, the peak of M-OH and M-O vibration appeared after 30 min test and further increased with the test time prolonging. However, the above evolution is absent for PtPdNiCo/C, which further verifies the above conclusion.

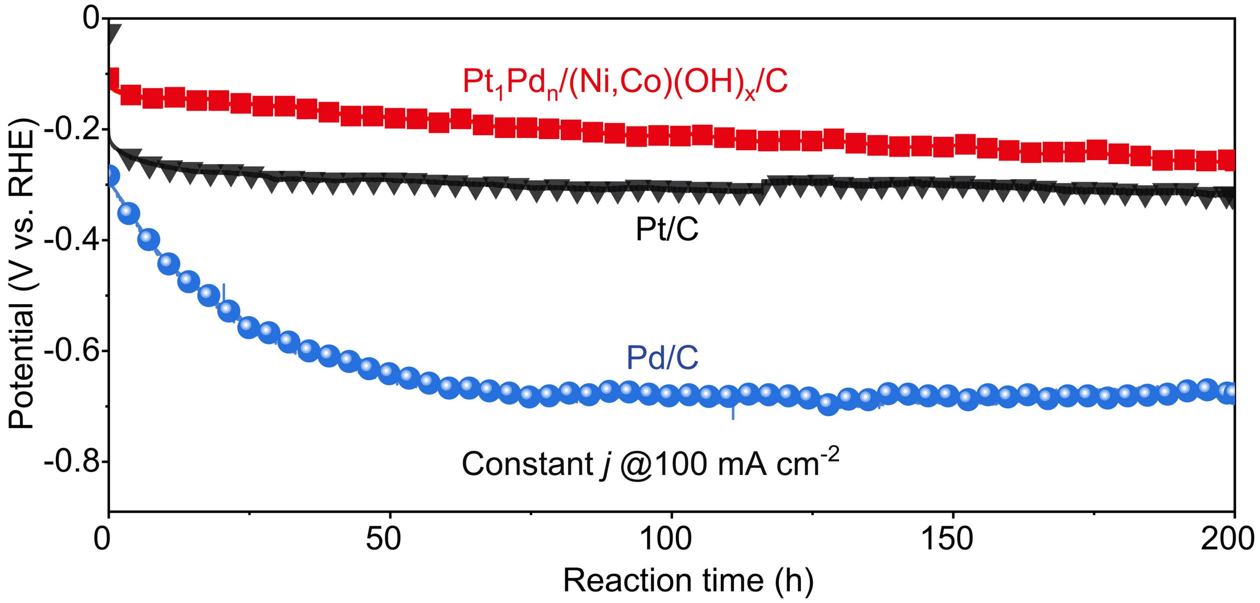

Figure S30. The chronopotentiometry response of the as-obtained catalysts at 100 mA cm^{-2} for 200 h.

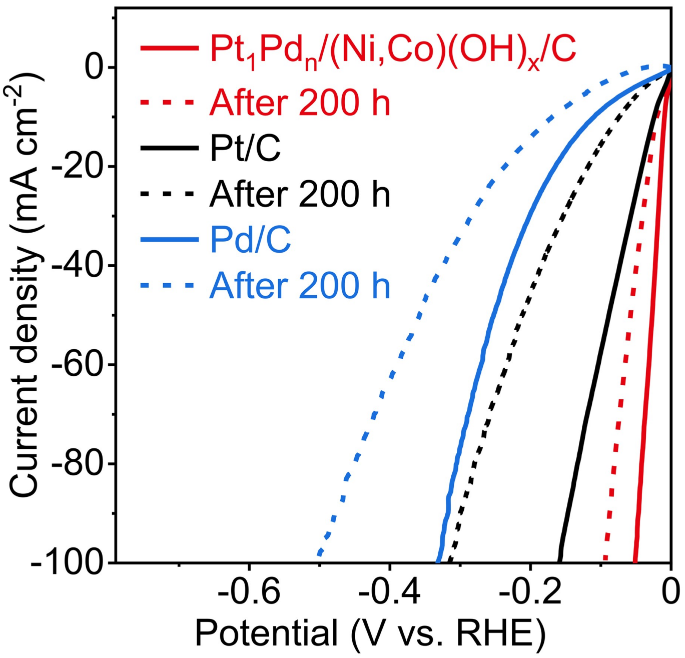

Figure S31. The polarization curves comparison of initial catalysts and after chronopotentiometry response at 100 mA cm^{-2} for 200 h.

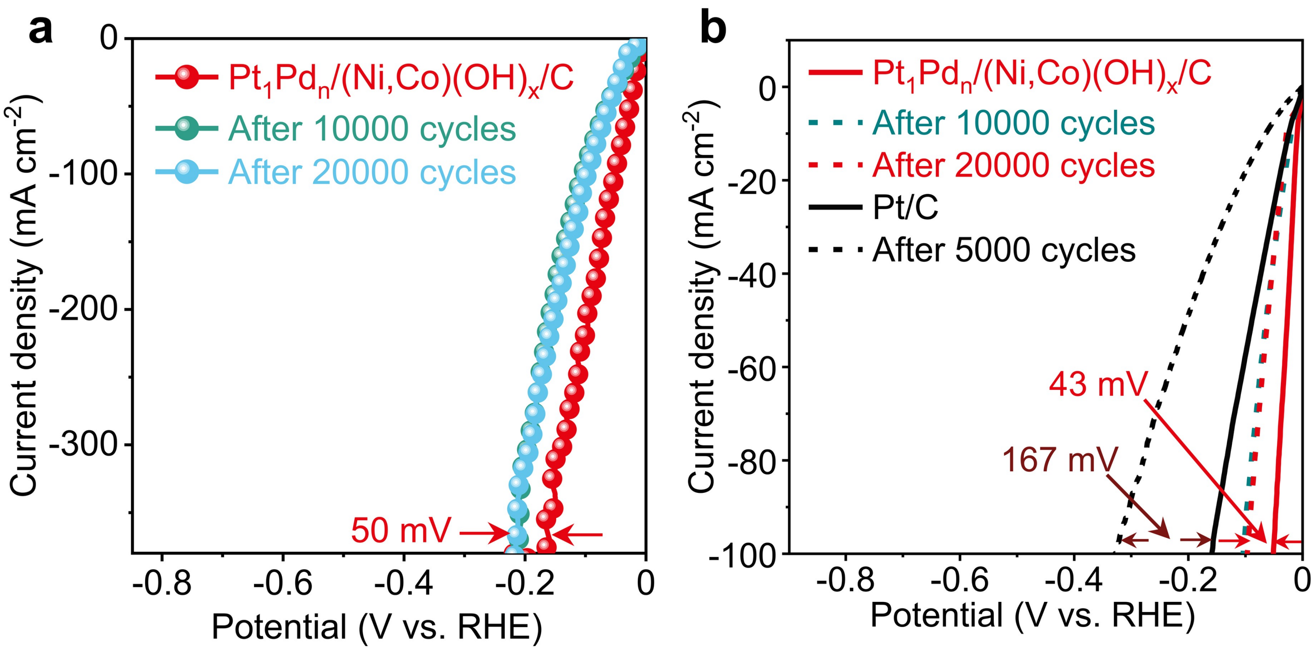

Figure S32. HER long-term cycle durability tests. (a) Polarization curves comparison at the current density of 380 mA cm^{-2} of the initial $Pt_1Pd_n/(Ni,Co)(OH)_x/C$ catalyst and after 10000 and 20000 cycles during the accelerated durability tests. (b) The contrast towards the polarization curves of initial $Pt_1Pd_n/(Ni,Co)(OH)_x/C$, after 10000 and 20000 cycles with the initial Pt/C and after 5000 cycles.

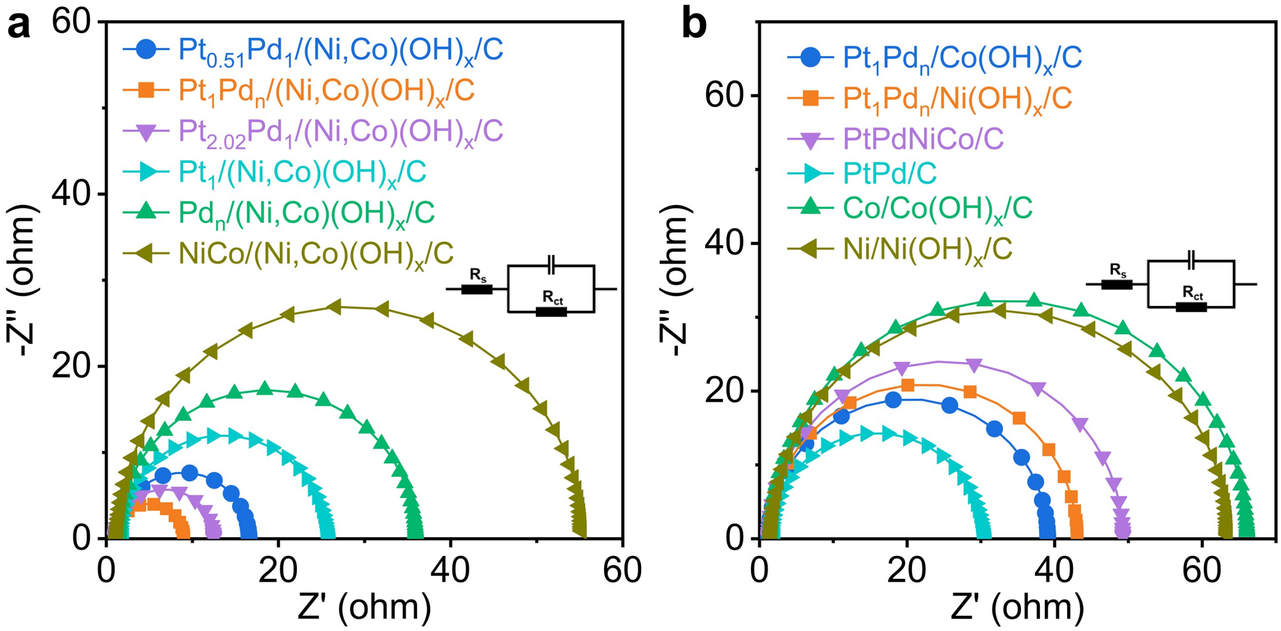

Figure S33. Nyquist plots of the as-obtained samples in 1.0 M KOH solution.

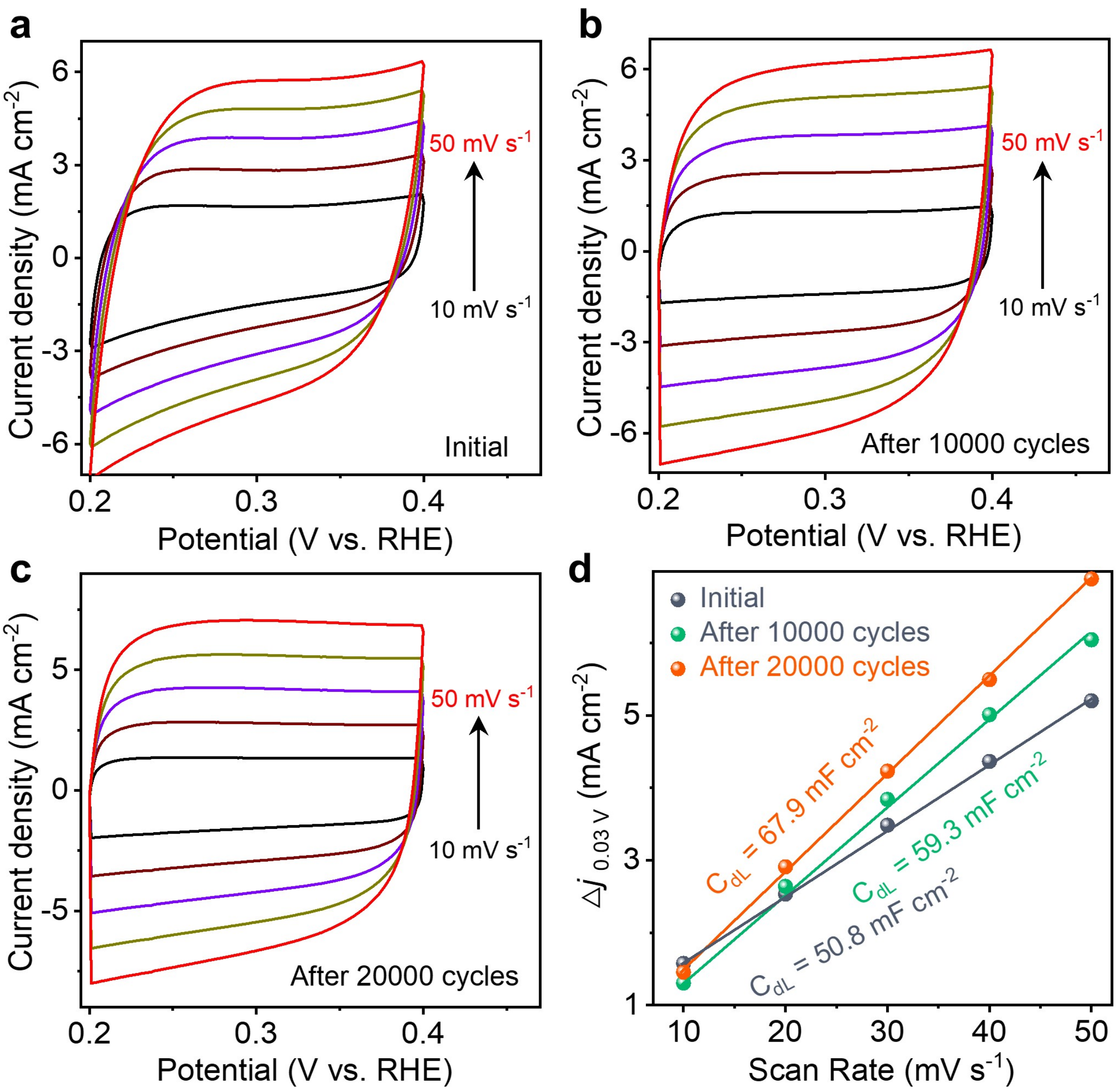

Figure S34. The cyclic voltammetry (CV) measurements and linear fitting of the capacitive currents versus CV scanning rate for the initial $Pt_1Pd_n/(Ni,Co)(OH)_x/C$ and after the HER. The cyclic voltammetry (CV) measurements tested within the range of 0.200 to 0.400 V versus RHE at different scanning rates (0.01-0.05 V s^{-1}) for (a) $Pt_1Pd_n/(Ni,Co)(OH)_x/C$, (b) after 10000 cycles and (c) after 20000 cycles in 1.0 M KOH solution and (d) linear fitting of the capacitive currents versus CV scanning rate for the initial $Pt_1Pd_n/(Ni,Co)(OH)_x/C$ and after the HER for 10000 cycles and 20000 cycles in 1.0 M KOH solution.

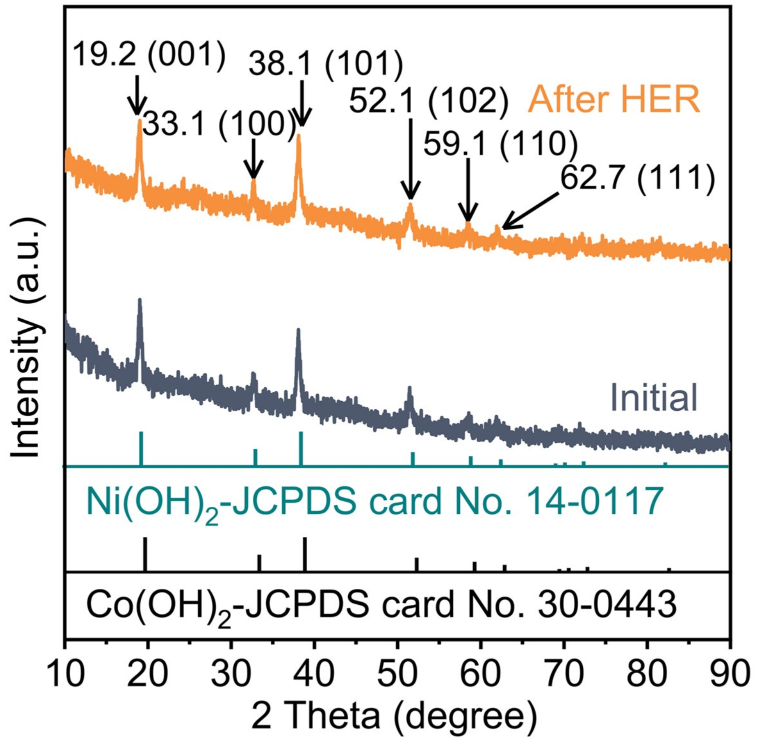

Figure S35. XRD patterns for the initial $Pt_1Pd_n/(Ni,Co)(OH)_x/C$ sample and reactivated after HER for 20000 cycles.

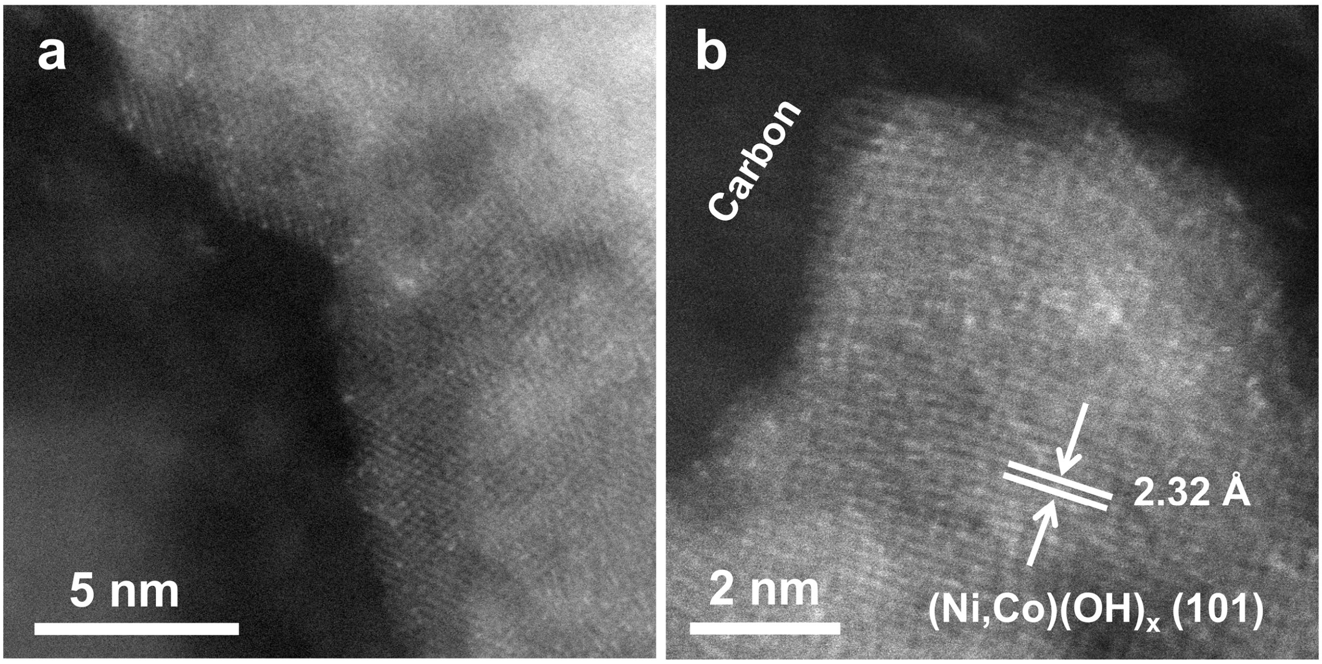

Figure S36. AC-STEM images of the $Pt_1Pd_n/(Ni,Co)(OH)_x/C$ sample after the HER for 20000 cycles. Scale bars: (a) 5 nm. (b) 2 nm.

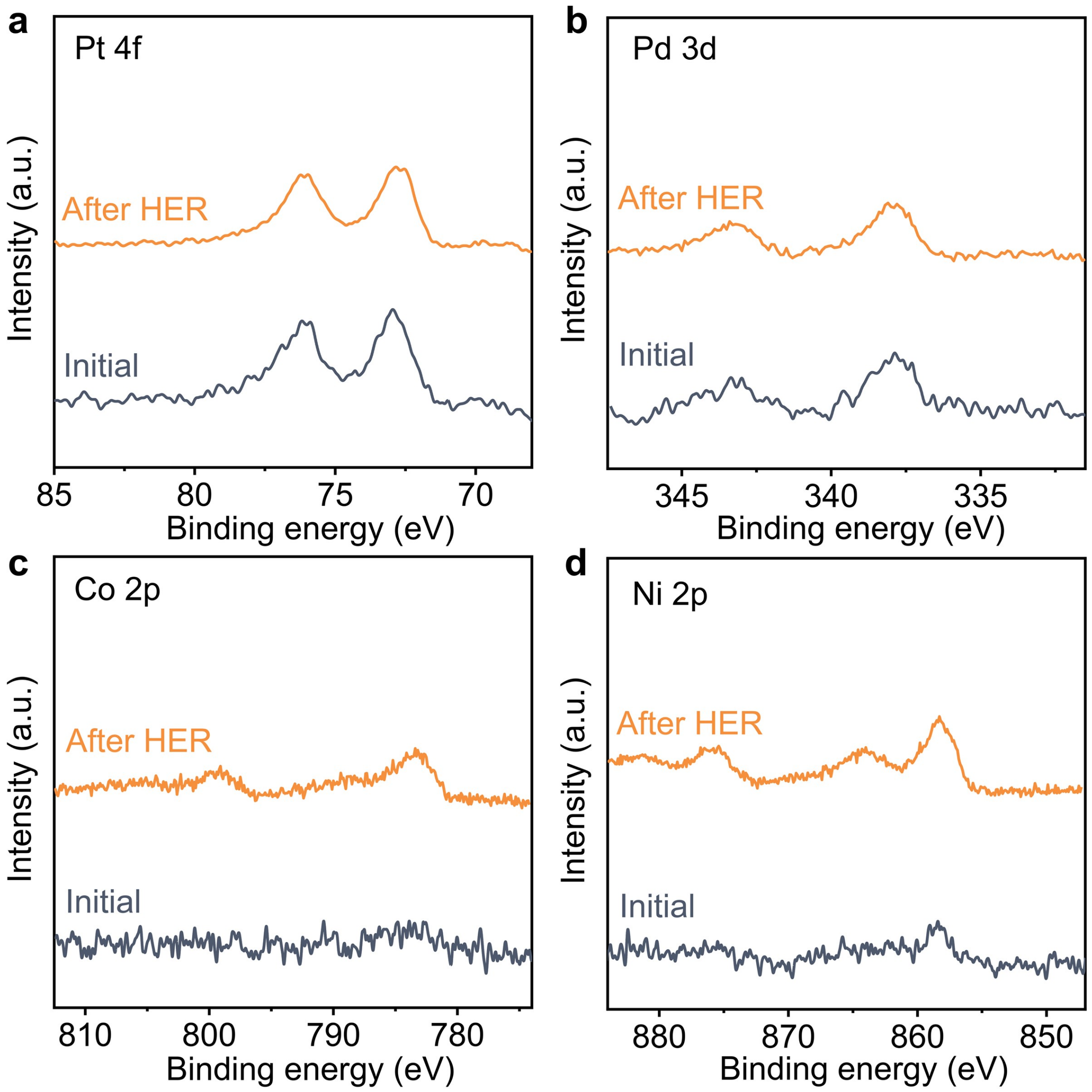

Figure S37. XPS spectra of initial $Pt_1Pd_n/(Ni,Co)(OH)_x/C$ sample and reactivated after HER for 20000 cycles.

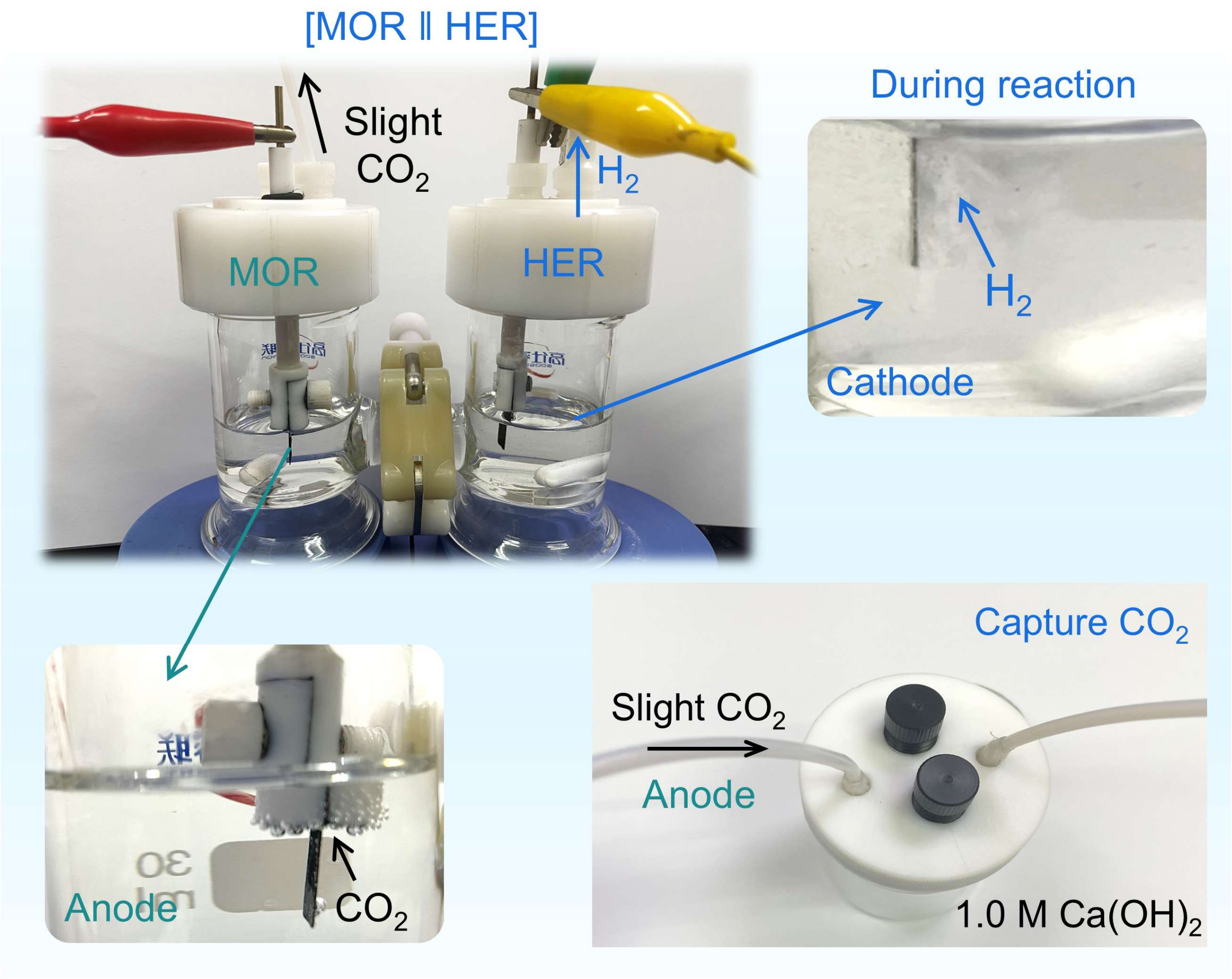

Figure S38. $Pt_1Pd_n/(Ni,Co)(OH)_x/C$ for MOR coupling HER. The electrolyte in the anode was 1.0 M KOH + 1.0 M methanol, 1.0 M KOH in the cathode and the device for capturing CO_2 in the anode with 1.0 M $Ca(OH)_2$ solution to produce high-value-added $CaCO_3$.

Note: Despite our determination via NMR spectroscopy revealing the catalyst's high selectivity towards formate at different voltage of 0.600 V to 1.550 V (versus RHE) (when test for 10000 s), $Pt_1Pd_n/(Ni,Co)(OH)_x/C$ confirmed its high formate selectivity (98.03%) and formate Faradaic efficiency (91.54%) at 0.900 V for MOR (Figure 3d). With methanol conversion approaching 100% at 0.800 V versus RHE, the intermediate of formate could be further electrooxidized to CO_2, $Pt_1Pd_n/(Ni,Co)(OH)_x/C$ confirmed its higher CO_2 selectivity (51.3%) and Faradaic efficiency (61.2%) than Pt/C (21.7%, 29.1%, respectively) (Figure 3e, Figure S38). Bubbles of CO_2 and H_2 were readily observed at the anode and the cathode chamber, respectively, and slight CO_2 was further captured by the additional conversion chamber with 1.0 M $Ca(OH)_2$ solution to obtain high value-added product (super purity $CaCO_3$) via the anode side (Figure S38). To mitigate this CO_2 emission, we devised a straightforward apparatus. This device enables

the capture of CO_2 at the anode using a 1.0 M $Ca(OH)_2$ solution, yielding high-value-added $CaCO_3$. $Ca(OH)_2 + CO_2 = CaCO_3 (s) + H_2O$ $\Delta H = - 114.4$ kJ mol^{-1}.

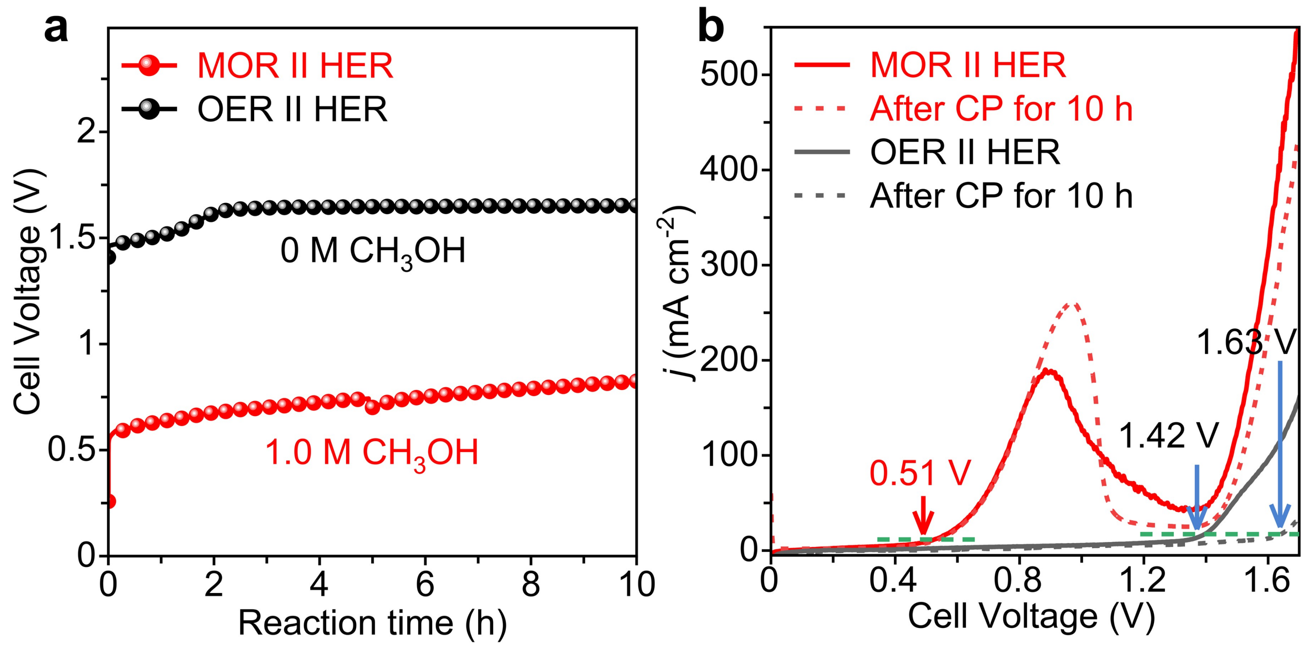

Figure S39. Chronopotentiometry responses of the [MOR||HER] cell and water splitting cell. (a) Chronopotentiometry responses at a constant current density of 10 mA cm^{-2} towards the [MOR||HER] cell and water splitting cell measurement for 10 h, 10 mA cm^{-2}. (b) The comparison of initial and after the chronopotentiometry of (a) the cell of MOR coupling HER and water splitting [OER||HER] electrolytic cell co-catalyzed by $Pt_1Pd_n/(Ni,Co)(OH)_x/C$ in 1.0 M KOH with/without 1.0 M methanol, 10 mV s^{-1}.

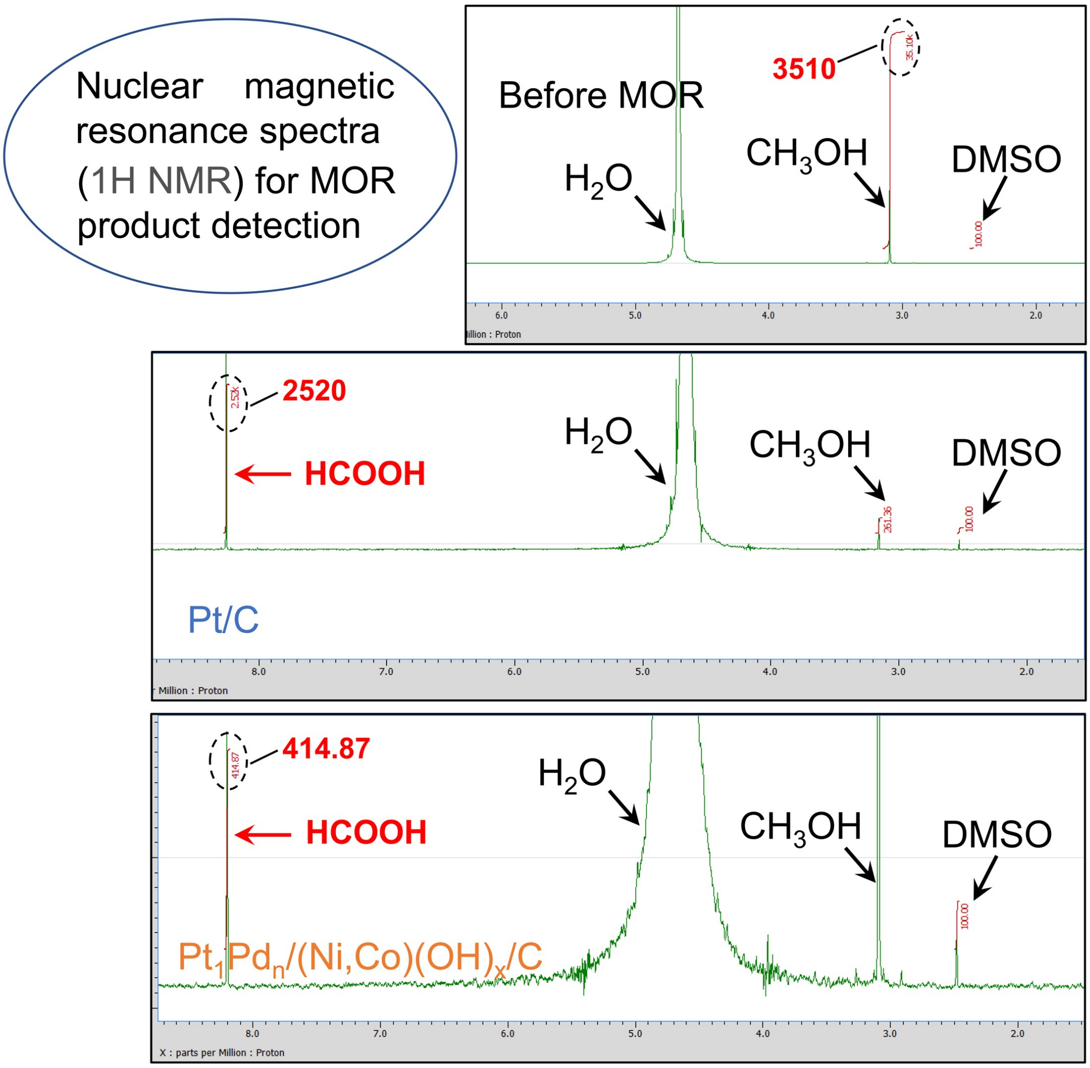

Figure S40. Representative nuclear magnetic resonance (NMR) spectra of the liquid products. NMR spectrum of the reaction products obtained upon MOR on $Pt_1Pd_n/(Ni,Co)(OH)_x/C$ and Pt/C catalyst at 0.800 V (versus RHE) using 1.0 M KOH + 1.0 M CH_3OH electrolyte to react until the current is almost zero. The largest singlet at 2.62 ppm corresponds to the reference DMSO signal, while the noisy part around 4.70 ppm is due to the suppressed water signal. The rest of the signal peaks are at 3.09 ppm, and 8.27 ppm corresponding to methanol, and formate, respectively.

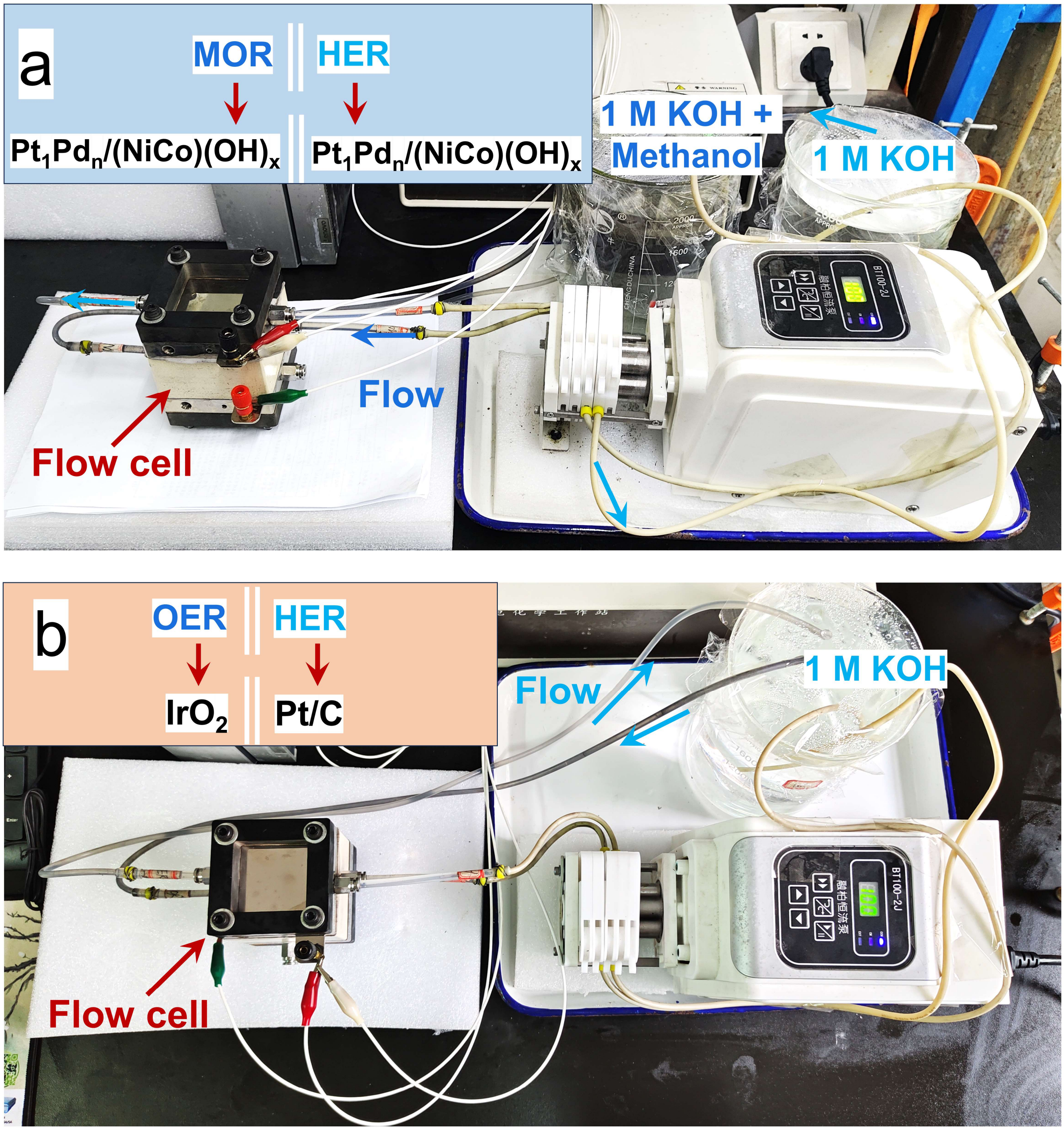

Figure S41. The flow cell performance under actual working conditions of $Pt_1Pd_n/(Ni,Co)(OH)_x/C$ for MOR coupling HER. (a) The electrolyte in the anode was 1.0 M KOH + 1.0 M methanol, 1.0 M KOH in the cathode. (b) The flow cell device of water electrolysis by IrO_2 for OER and Pt/C for HER.

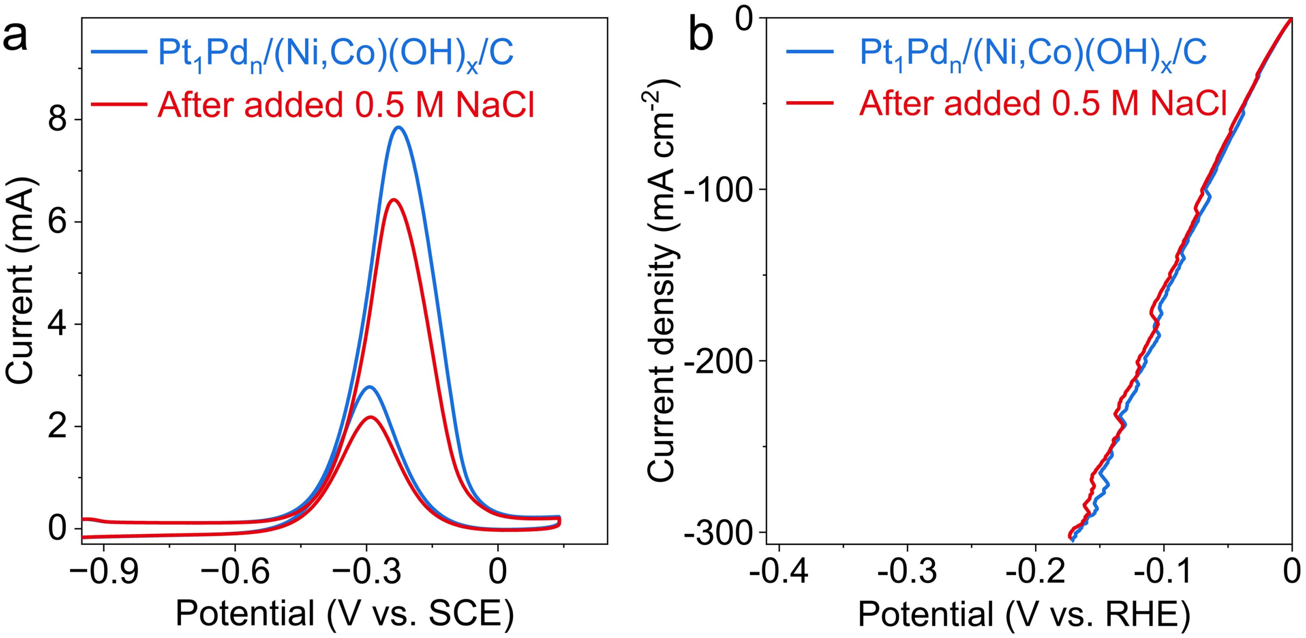

Figure S42. MOR and HER performance test at the simulated seawater environment. (a) The CV curves of the $Pt_1Pd_n/(Ni,Co)(OH)_x/C$ catalyst in 1.0 M KOH + 1.0 M methanol with/without 0.5 M NaCl. (b) the polarization curve of the $Pt_1Pd_n/(Ni,Co)(OH)_x/C$ for HER in 1.0 M KOH with/without 0.5 M NaCl.

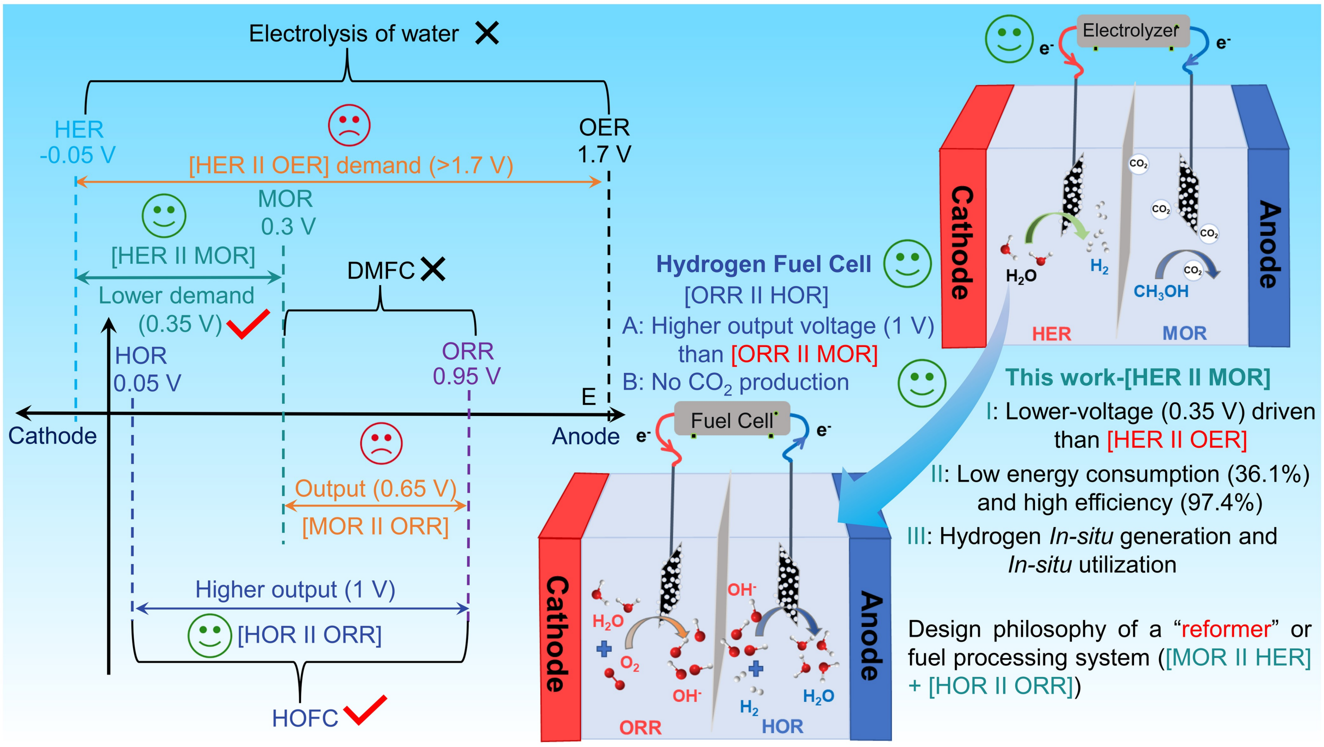

Figure S43. The comparison of different coupling devices. The transformation from DMFC to hydrogen fuel cells by *in-situ* extraction and utilization of hydrogen from methanol is promising.

Note: As a liquid at ambient conditions, methanol has the highest hydrogen content among alcohols or biomass of the same mass as a convenient hydrogen carrier: it is much easier and safer to store, deliver, and distribute, also for a much longer time, simply using existing infrastructure at little extra cost. Moreover, methanol can be generated by various low-carbon approaches, e.g., from biomass and waste, to reduce its "well-to-wheel" emissions (up to 98%, to 2 g CO_2/km, versus the conventional syngas reforming with 83 g CO_2/km). It is thus very desirable to develop an electrochemical process of "methanol and water co-electrolysis" to produce hydrogen.

Figure S43 shows the advantages of the rational coupling of MOR and HER. The potential of anodic alcohol oxidation and cathodic oxygen reduction are relatively close, and the traditional direct methanol fuel cells (DMFCs) often show a lower power output (voltage = 0.650 V) than hydrogen fuel cells (1.000 V). The transformation from DMFCs to hydrogen fuel cells by *in-situ* extraction and utilization of hydrogen from methanol is promising. The cost-effective [HER||MOR] was used to replace the traditional water decomposition for efficient H_2 generation to power-assisted DMFCs-[ORR||MOR] transform to HOFCs-[ORR||HOR]. The *in-situ* extraction and utilization of hydrogen from methanol are promising to convert the traditional DMFC with low voltage output (0.650 V) to hydrogen fuel cells (1.000 V). We

report a design philosophy of a "reformer" or fuel processing system ([MOR||HER] + [HOR||ORR]) by the synergistic ensemble catalyst of Pt single atoms and Pd nanoclusters anchored on $(Ni,Co)(OH)_x$ then supported on carbon black, $Pt_1Pd_n/(Ni,Co)(OH)_x/C$. The energy consumption of a [MOR||HER] cell with the catalyst is only 36.1% that of an [OER||HER] cell for water-splitting hydrogen evolution and its voltage efficiency is up to 97.4% by the synergistic effect of Pt single atoms, Pd nanoclusters and $(Ni,Co)(OH)_x$.

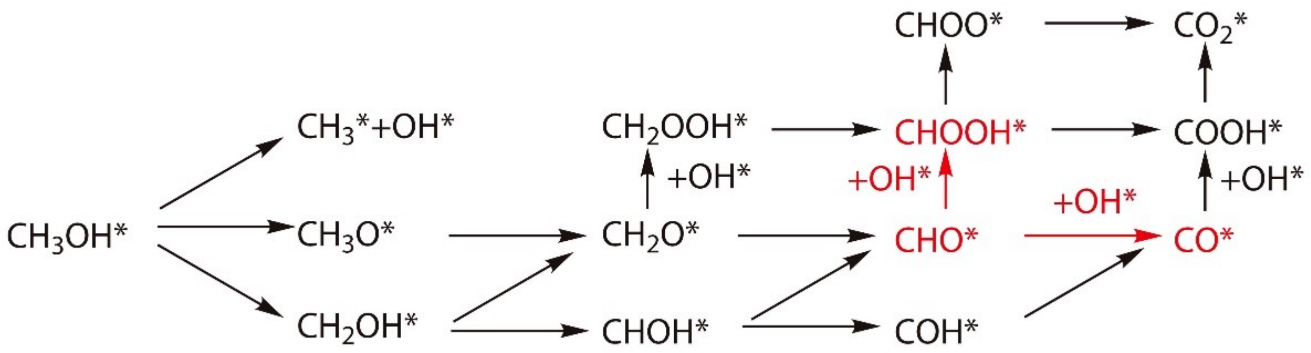

Figure S44. Possible reaction pathways of MOR.

Note: The possible reaction pathways of the MOR [*J. Phys. Chem. C* **119**, 20389-20400 (2015).] are schematically shown here in Figure S44. Calculating the whole pathways for all catalysts involved in this study would be very computationally resource-consuming. One of the main advantages of our new catalyst in this study is the high selectivity towards CO_2 during the MOR which can avoid CO-poisoning effectively. Therefore, only the part highlighted in red color was calculated. The pathway of *CHO + *OH → *CHOOH would lead to the production of CO_2, while the pathway of *CHO + *OH → *CO + H_2O would lead to CO-poisoning.

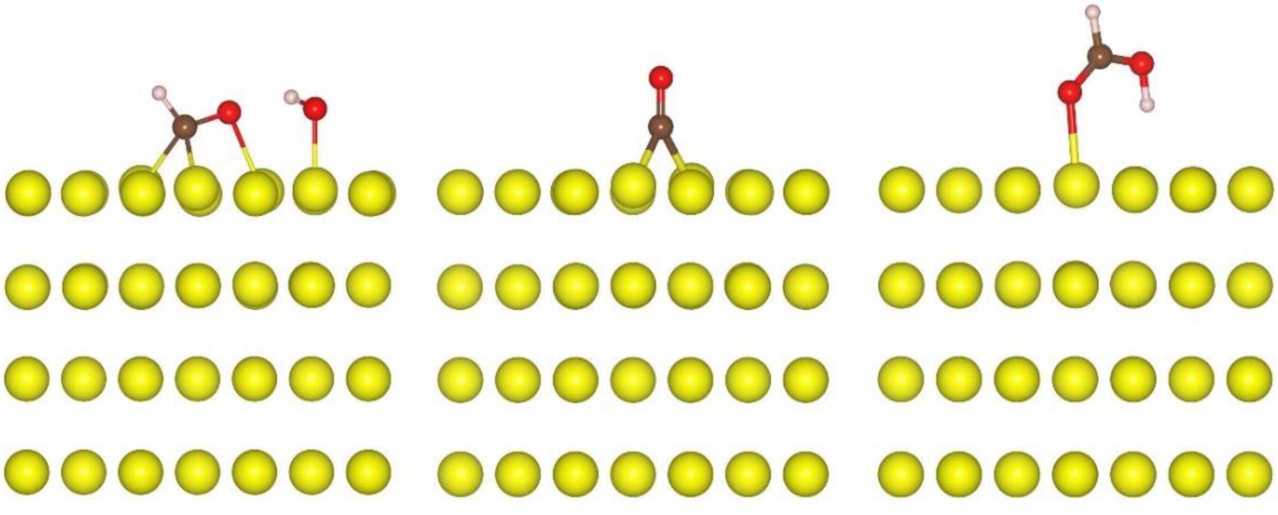

Figure S45. Atomic structures of involved intermediates during the formation of *CHOOH and *CO on Pt(111). The red, brown, grey, yellow, and white balls are for O, C, Pt and H atoms, respectively.

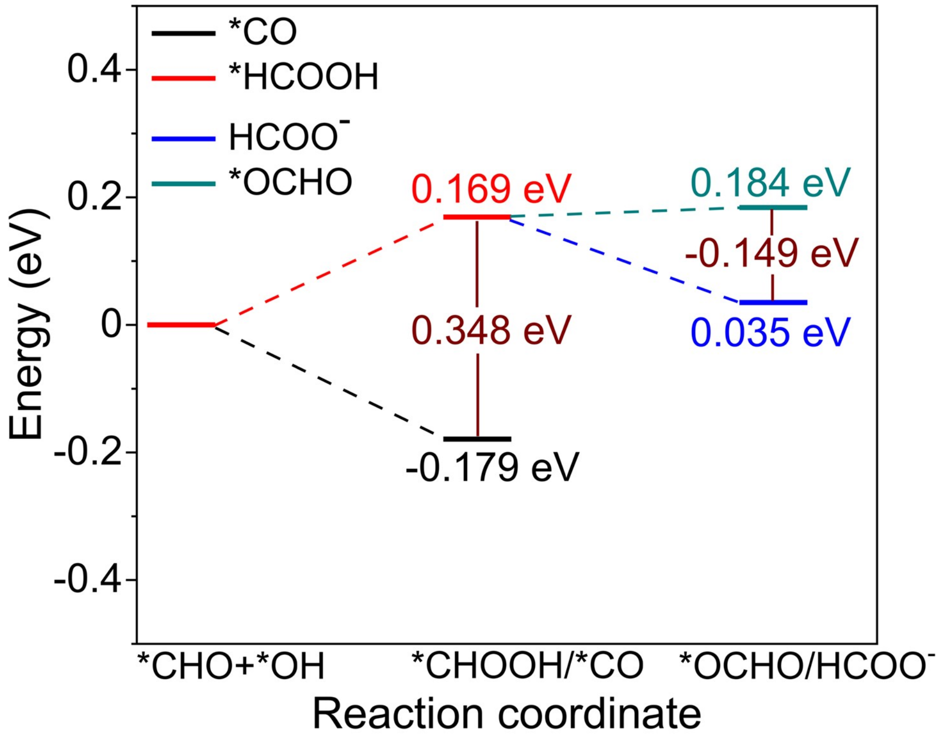

Figure S46. Free energy diagrams for the formation of *CO, *CHOOH, $HCOO^-$, and *OCHO on $Pd_n/(Ni,Co)(OH)_x$.

Note: As presented in the main text $\Delta G_1 = \Delta G*_{CHOOH} - \Delta G*_{CO}$ is a good descriptor of the selectivity. A less positive ΔG usually indicates a better selectivity towards *CHOOH. As shown in Fig. 4a, b, ΔG for Pt(111) and $Pt_1Pd_n/(Ni,Co)(OH)_x$ are 0.860 and -0.076 eV, respectively. Here, ΔG for $Pd_n/(Ni,Co)(OH)_x$ is 0.350 eV. Therefore, the performance order of the catalysts is Pt(111) < $Pd_n/(Ni,Co)(OH)_x$ < $Pt_1Pd_n/(Ni,Co)(OH)_x$ considering the selectivity towards CO_2. While $\Delta G_2 = \Delta G_{HCOO^-} - \Delta G_{*OCHO}$ is a good descriptor of this selectivity. A more positive ΔG_2 usually suggests a better selectivity towards $HCOO^-$. Being consistent with the value of ΔG_2, the production content of $HCOO^-$ follows the following trend: Pt(111) > $Pd_n/(Ni,Co)(OH)_x$ > $Pt_1Pd_n/(Ni,Co)(OH)_x$.

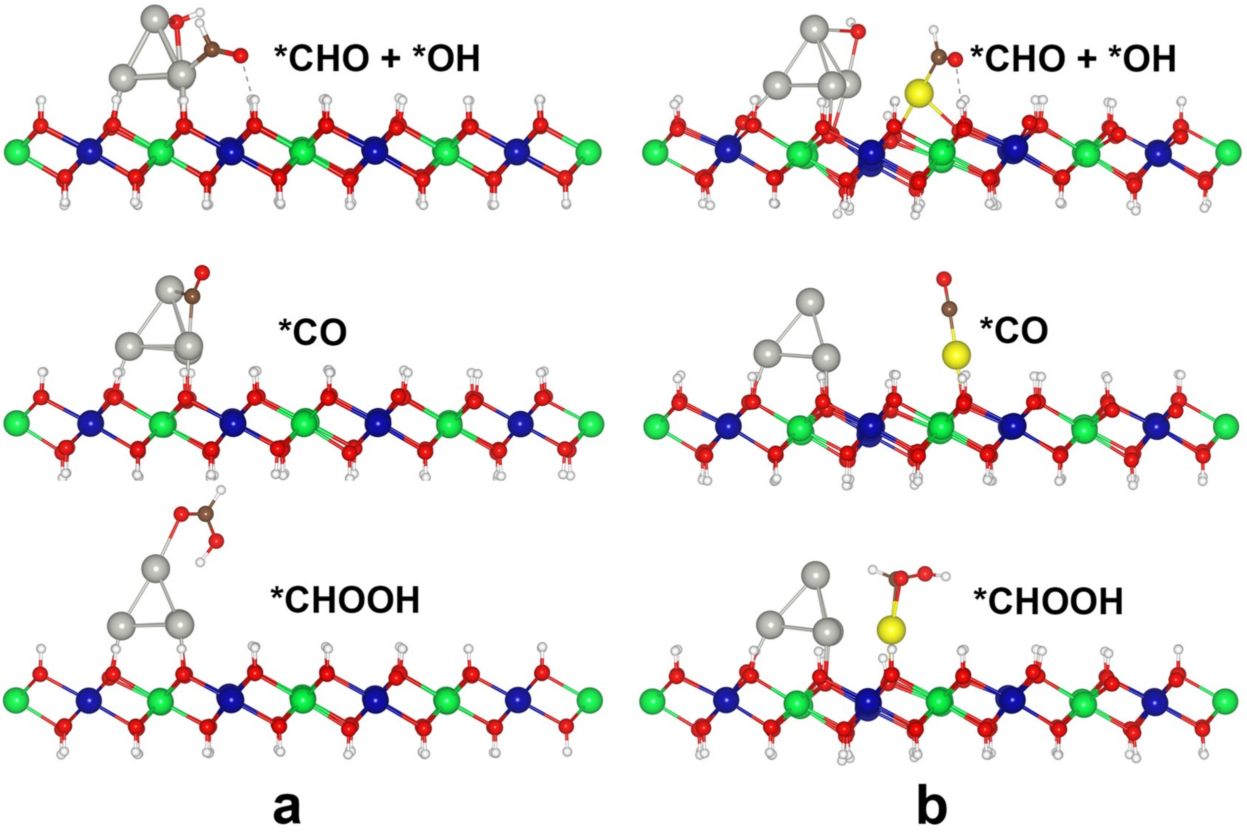

Figure S47. Atomic structures of involved intermediates during the formation of *CHOOH and *CO. (a) Pd_n/(Ni,Co)$(OH)_x$ and (b) Pt_1Pd_n/(Ni,Co)$(OH)_x$.

Note: The model of the same process for noble metal on (Ni,Co)$(OH)_x$ is shown in **Figure S47**. Hydrogen bonds between intermediates and (Ni,Co)$(OH)_x$ are presented by dash lines. The red, brown, green, blue, grey, yellow and white balls are for O, C, Co, Ni, Pt and H atoms, respectively.

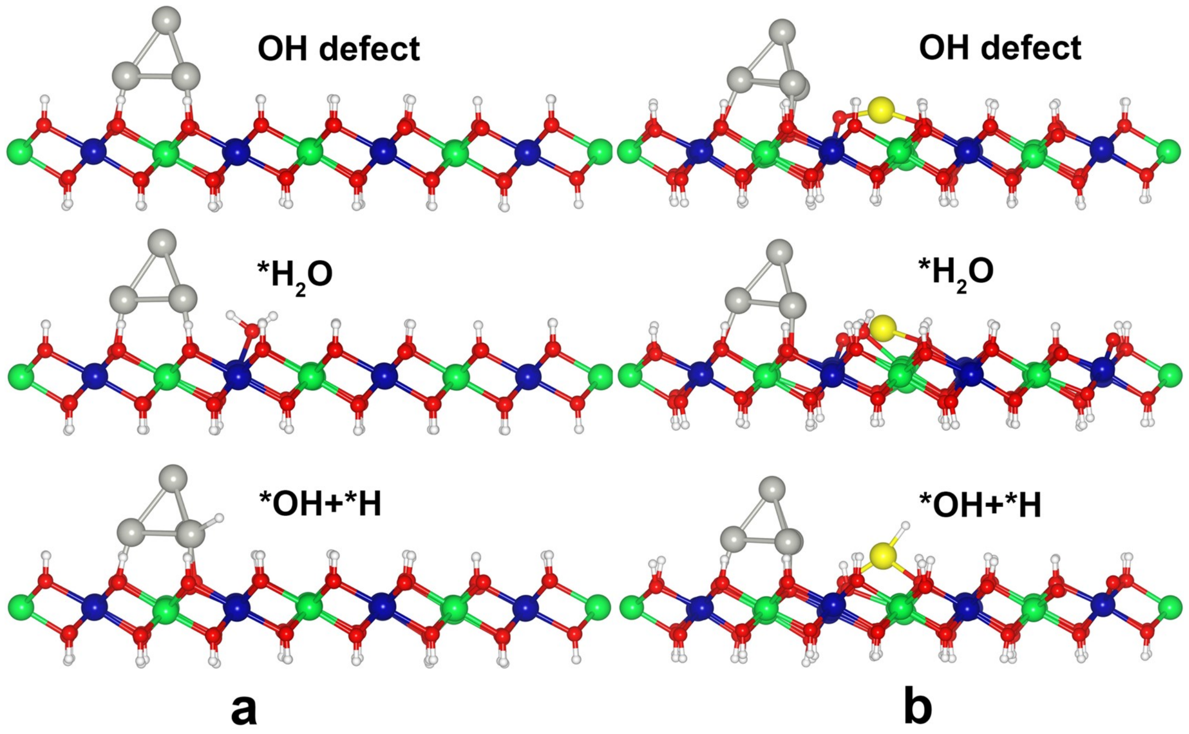

Figure S48. Atomic structures of water adsorption and dissociation on (a) Pd_n/(Ni,Co)$(OH)_x$ and (b) Pt_1Pd_n/(Ni,Co)$(OH)_x$. The red, green, blue, yellow, grey and white balls are for O, Co, Ni, Pt, Pd and H atoms, respectively.

Note: To illustrate the advantage of the water-splitting process on Pd_n/(Ni,Co)$(OH)_x$ and Pt_1Pd_n/(Ni,Co)$(OH)_x$, the relaxed atomic structures are shown below. The water molecular would like to be captured by OH defect on (Ni,Co)$(OH)_x$ and then split into *OH on OH defect and *H on the nearest noble atom. This process is more energetically favorable than on pure Pt(111).

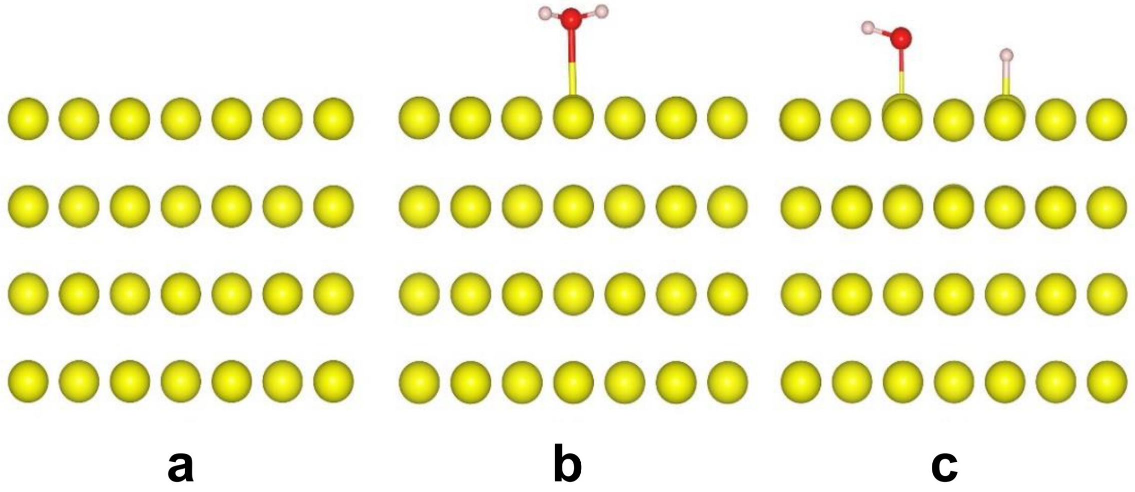

Figure S49. Atomic structures of water adsorption and dissociation on Pt(111). The model of the same process for noble metal on (Ni,Co)$(OH)_x$ is shown in **Figure S44**. The red, yellow and white balls are for O, Pt and H atoms, respectively.

Note: To illustrate the water-splitting process on pure Pt(111), the relaxed atomic structures are shown below. Compared with the same process for noble metal on (Ni,Co)$(OH)_x$, this process for Pt(111) would be more sluggish.

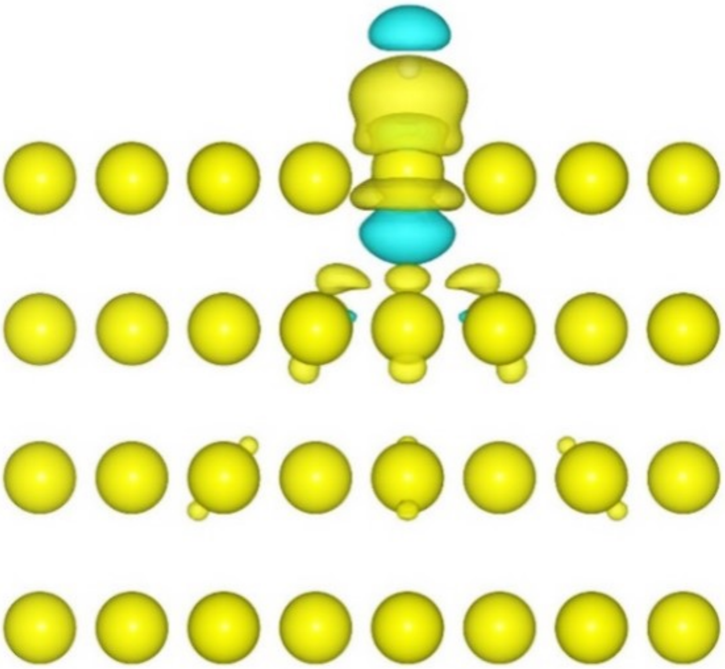

Figure S50. Charge density difference for the adsorption of one H on Pt(111). The yellow and white balls are for Pt and H atoms, respectively.

Note: The charge density difference of one H adsorbed on Pt(111) vividly shows that electrons accumulate between Pt and H and deplete on these two atoms. Therefore, the bond between Pt and H is covalent. The more local negative charges noble metal has, the more electrons it can offer to form a bond with H and a stronger covalent bond should be expected. Bader charge analysis would help to figure out the adsorption strength of H on different catalysts.

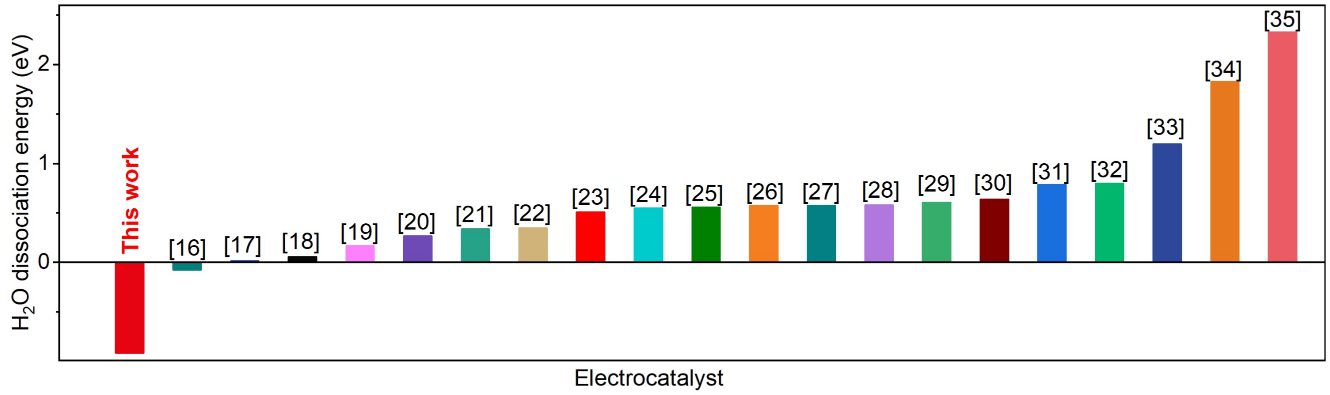

Figure S51. Comparison of this work ($Pt_1Pd_n/(Ni,Co)(OH)_x/C$, -0.917 eV) and summary of water dissociation ability and energy of different reported catalysts. Including the $Pt_1/(Co,Ni)(OH)_2$ (-0.074 eV)[16], $Co(OH)_2$ (0.02 eV)[17], Pt_1-C_2 (0.06 eV)[18], Ru-doped $SrTiO_3$ (0.17)[19], Co_3S_4 PNS_{vac} (0.27 eV)[20], O, Cu-CoPO nanowire (0.34 eV)[21], RhO_2 (0.35 eV)[22], α-MoC_{1-x} (0.51 eV)[23], Ru-N_4 (0.550 eV)[24], Mo exposed NiMoP (0.56 eV)[25], pyridinic-N-MoP (0.58 eV)[26], NiO/Pt (0.58 eV)[27], Cr-Co_4N (0.582 eV)[28], Mn-hcp Ni (0.61 eV)[29], MoS_2/LDH (0.64 eV)[30], 1T-MoS_2/SWNT (0.79 eV)[31], $CoNiS_2$ (0.80 eV)[32], GN_2@RuMo (1.2 eV)[33], $MoS_2/Ni(OH)_2$ (1.83 eV) [34], Ni_3N (2.33 eV)[35].

Note: We made a summary and comparison of the capabilities for water dissociation between $Pt_1Pd_n/(Ni,Co)(OH)_x/C$ and various other reported catalysts, clearly showing that our $Pt_1Pd_n/(Ni,Co)(OH)_x/C$ possessed the appropriate water dissociation energy.

Table S1. Best-fit EXAFS parameters of $Pt_1Pd_n/(Ni,Co)(OH)_x/C$, PtPdNiCo/C, $Pt_1Pd_n/Ni(OH)_x/C$, $Pt_1Pd_n/Co(OH)_x/C$, $NiCo/(Ni,Co)(OH)_x/C$, Ni foil, $Ni(OH)_2$, Co foil, $Co(OH)_2$, Pt foil, Pd foil.[a]

Sample	Scattering path	CN	σ^2 (Å^2)	E_F	R (Å)	Δk	ΔR	R_f
Co foil	Co-Co	8.9	0.006	7.7	2.5	3.1-14.3	1.0-3.0	0.003
$Co(OH)_2$	Co-O	3.2	0.004	-27.6	1.8	3.0-13.5	1.0-3.0	0.05
	Co-Co	3.9			3.1			
Ni foil	Ni-Ni	9.6	0.006	6.5	2.5	3.1-14.5	1.0-3.0	0.001
$Ni(OH)_2$	Ni-O	6.3	0.007	-3	2.1	3.0-16.7	1.0-3.0	0.008
	Ni-Ni	6.5			3.1			
Pd foil	Pd-Pd	7.14	0.004	-2.6	2.7	3.6-11.2	1.0-3.0	0.005
Pt foil	Pt-Pt	10	0.005	7.8	2.8	2.4-14.7	1.0-3.0	0.002
			Co *K*-edge					
$Pt_1Pd_n/(Ni,Co)(OH)_x/C$	Co-O	5.42	0.016	1.53	2.1	2.9-11.3	1-3.15	0.02
	Co-Co	10.71			3.1			
	Co-Pd	1.78			2.7			
PtPdNiCo/C	Co-O	2.9	0.01	-29.6	2.2	2.7-11.6	1.2-3	0.03
	Co-Co	4.1			2.4			
	Co-Pd	6.5			2.6			
$Pt_1Pd_n/Co(OH)_x/C$	Co-O	3.62	0.010	-19.14	1.9	2.9-11	1.0-3.0	0.05
	Co-Co	3.39			3.1			
	Co-Pd	2.24			2.6			
$NiCo/(Ni,Co)(OH)_x/C$	Co-O	5.65	0.008	-1.10	2.1	2.8-10.8	1.0-3.0	0.005
			Ni *K*-edge					
$Pt_1Pd_n/(Ni,Co)(OH)_x/C$	Ni-O	6.198	0.008	-2.880	2.1	2.86-12.5	1.0-3.0	0.02
	Ni-Ni	5.346			3.1			
PtPdNiCo/C	Ni-O	2.707	0.009	-5.613	2.1	2.75-12.6	1.3-2.5	0.001
	Ni-Ni	5.196			2.5			
$Pt_1Pd_n/Ni(OH)_x/C$	Ni-O	6.63	0.009	-1.509	2.1	2.9-11	1.0-3.0	0.02
	Ni-Ni	4.716			3.1			

NiCo/(Ni,Co)(OH)$_x$/C	Ni-O	6.006	0.007	-3.261	2.1	2.8-10.8	1.0-3.0	0.01
	Ni-Ni	5.31			3.1			
				Pd *K*-edge				
Pt$_1$Pd$_n$/(Ni,Co)(OH)$_x$/C	Pd-Pd	4.28	0.01	-0.51	2.8	3.6-11.2	1.7-3	0.02
	Pd-Co(Ni)	0.98			2.7			
PtPdNiCo/C	Pd-Pd	4.76	0.01	-0.57	2.7	3.6-11.2	1.15-3	0.03
	Pd-Co(Ni)	2.37			2.6			
Pt$_1$Pd$_n$/Ni(OH)$_x$/C	Pd-Pd	10.3	0.01	-5.98	2.7	2.9-11	1.0-3.0	0.02
	Pd-Ni	4.83			2.6			
Pt$_1$Pd$_n$/Co(OH)$_x$/C	Pd-Pd	9.07	0.02	-3.18	2.7	3.5-9.8	1.0-3.0	0.05
	Pd-Co	4.34			2.6			
				Pt *L$_3$*-edge				
Pt$_1$Pd$_n$/(Ni,Co)(OH)$_x$/C	Pt-O	2.7	0.003	18.13	2.09	2.5-8.0	1.0-3.0	0.01
PtPdNiCo/C	Pt-O	1.51	0.007	3.73	2.0	2.6-9.2	1.2-3.2	0.015
	Pt-Pt	8.04		3.73	2.7			
Pt$_1$Pd$_n$/Ni(OH)$_x$/C	Pt-O	4.1	0.007	16.7	2.08	2.5-8.0	1.0-3.0	0.004
Pt$_1$Pd$_n$/Co(OH)$_x$/C	Pt-O	3.2	0.004	17.03	2.08	2.5-8.0	1.0-3.0	0.0006

[a] CN, coordination number; R, the distance between absorber and backscatter atoms; σ^2, Debye-Waller factor value; Δk, data range for fitting in k-space; ΔR, data range for fitting in R-space; R_f, R-factor characterizing the goodness of fit. Error bounds (accuracies) characterizing the structural parameters obtained by EXAFS data analysis are estimated to be as follows: CN, ±20%; R, ±1% and σ^2, ±20%.

Table S2. Mass activity and the corresponding potential of as-obtained catalysts, Pt/C and Pd/C for the methanol oxidation reaction.

Catalysts	E_f (V)	I_f (A mg_{PtPd}^{-1})	E_b (V)	I_b (A mg_{PtPd}^{-1})
$Pt_1Pd_n/(Ni,Co)(OH)_x/C$	-0.191	7.796	-0.366	1.478
$Pt_{0.51}Pd_1/(Ni,Co)(OH)_x/C$	-0.185	5.070	-0.37	1.689
$Pt_{2.02}Pd_1/(Ni,Co)(OH)_x/C$	-0.245	4.894	-0.353	1.167
$Pt_1Pd_n/Co(OH)_x/C$	-0.188	4.280	-0.372	0.941
$Pt_1Pd_n/Ni(OH)_x/C$	-0.261	3.078	-0.333	1.302
$Pt_1/(Ni,Co)(OH)_x/C$	-0.234	3.257	-0.366	0.902
$Pd_n/(Ni,Co)(OH)_x/C$	-0.231	2.852	-0.364	0.797
$NiCo/(Ni,Co)(OH)_x/C$	-	-	-	-
$Co/Co(OH)_x/C$	-	-	-	-
$Ni/Ni(OH)_x/C$	-	-	-	-
PtPdNiCo/C	-0.316	0.281	-0.413	0.004
PtPd/C	-0.227	1.801	-0.379	0.430
Pd/C	-0.239	0.200	-0.372	0.049
Pt/C	-0.269	0.403	-0.368	0.127

E_f: Forward sweep peak potential (V versus SCE); E_b: Backward sweep peak potential (V versus SCE), I_f: Forward sweep peak current normalized by the loading of Pt and Pd (A mg_{PtPd}^{-1}); I_b: Backward sweep peak current normalized by the loading of Pt and Pd (A mg_{PtPd}^{-1}). "-": The material showed no reactive activity.

772 **Table S3. Comparison of the MOR activity between $Pt_1Pd_n/(Ni,Co)(OH)_x/C$ and reported noble metal electrocatalysts. NA: not available; *:**
773 **extracted from paper's data.**

Electrocatalysts	Electrolyte	MA(mA mg_{PtPd}^{-1})	Durability	References
$Pt_1Pd_n/(Ni,Co)(OH)_x/C$	1.0 M KOH+1.0 M CH_3OH	7796	93.1% activity retention after 4000 s	This work
$Pt/Ni(OH)_2/RGO$	1.0 M KOH+1.0 M CH_3OH	1236	90% activity retention after 3600 s	*Nat. Commun.*, 2015, 6, 10035
Pt_1/RuO_2	1.0 M KOH+1.0 M CH_3OH	6766	95.5 % activity retention after 36000 s	*Nat. Commun.*, 2021, 12, 5235.
SANi-Pt NWs	1.0 M KOH+1.0 M CH_3OH	7930	~75%* activity retention after 3600 s	*Nat. Catal.*, 2019, 2, 495
PtZn/MWNT	1.0 M KOH+0.5 M CH_3OH	~550	~65%* activity retention after 900 s	*J. Am. Chem. Soc.*, 2017, 139, 4762-4768.
PtCu NFs	0.5 M KOH + 1.0 M CH_3OH	2260	NA	*Adv. Mater.*, 2016, 28, 8712-8717.
$Pd-CeO_2/NMCS$	1.0 M KOH+1.0 M CH_3OH	1500	77.8% activity retention after 3600 s	*ACS Catal.*, 2019, 9, 6362.
Pt-Co NFs	1.0 M KOH+1.0 M CH_3OH	5600	30.6% activity retention after 1800 s	*Nano Lett.*, 2020, 20, 1974-1979.
Pt_2Bi nanochains	1.0 M KOH+1.0 M CH_3OH	4611	33.0% activity retention after 10000 s	*Nano Lett.*, 2020, 20, 7751-7759.
$Pt_{56}Cu_{28}Ni_{16}$	1.0 M KOH+1.0 M CH_3OH	7000±500	53% activity retention after 3600 s	*Nano Lett.*, 2019, 19, 5431-5436.

Table S4. Comparison of the HER activity between $Pt_1Pd_n/(Ni,Co)(OH)_x/C$ and other reported state-of-the-art electrocatalysts.

Electrocatalysts	Electrolyte	η_{10} (mV)	η_{50} (mV)	Tafel slope (mV dec^{-1})	References
$Pt_1Pd_n/(Ni,Co)(OH)_x/C$	1.0 M KOH	10	26.9	19.8	This work
Pt_{SA}/OLC	0.5 M H_2SO_4	38	-	36	*Nat. Energy*, 2019, 4, 512.
Au-Ru NWs	1.0 M KOH	50	-	30.8	*Nat. Chem.*, 2018, 10, 456-461.
$Mo_2TiC_2T_x$-Pt_{SA}	0.5 M H_2SO_4	30	-	30	*Nat. Catal.*, 2018, 1, 985
Ru-1.0	1.0 M KOH	13	-	25	*Nat. Commun.*, 2022, 13, 3958.
Ru-Mo_2C@CNT	1.0 M KOH	15	-	26	*Nat. Commun.*, 2021, 12, 4018.
$Pt_1/(Co,Ni)(OH)_2/C$	1.0 M KOH	24	65	28.7	*Energy & Environ. Sci.*, 2023, 16, 1035.
Pt_{SA}-Mn_3O_4	1.0 M KOH	24	-	54	*Energy Environ. Sci.*, 2022, 15, 4592-4600.
Ru@MWCNT	1.0 M KOH	17	-	27	*Nat. Commun.*, 2020, 11, 1278.
Pt/np-$Co_{0.85}Se$	1.0 M KOH	58	-	39	*Nat. Commun.*, 2019, 10, 1-9.
Pt-1/$MoSe_2$	1.0 M KOH	29	-	34	*Nat. Commun.*, 2021, 12, 3021.
Ni_5P_4-Ru/CC	1.0 M KOH	54	-	52	*Adv. Mater.*, 2020, 32, 1906972.
Pt_{SA}-NiO/Ni	1.0 M KOH	26	-	27	*Nat. Commun.*, 2021, 12, 3783.
Sr_2RuO_4	1.0 M KOH	61	-	51	*Nat. Commun.*, 2019, 10, 1.
Pt_{SA}/S-C	0.5 M H_2SO_4	53	-	46.9	*Nat. Commun.*, 2019, 10, 4977
Pt_{SA}-$Co(OH)_2$@Ag NWs	1.0 M KOH	29	-	35.7	*Energy Environ. Sci.*, 2020, 13, 3082-3092.
Ru@CN-0.16	1.0 M KOH	284	-	27.8	*Energy Environ. Sci.*, 2018, 11, 800.
Ru_2P/WO_3@NPC	1.0 M KOH	15	-	18	*Angew. Chem. Int. Ed.*, 2021, 60, 4110-4116.

Table S5. Comparison of MOR electro-oxidation between $Pt_1Pd_n/(Ni,Co)(OH)_x/C$ and reported state-of-the-art non-noble metal electrocatalysts.

Electrocatalysts	Electrolyte	Potential (V vs. RHE)	Current density ($mA\ cm^{-2}$)	Scan rate ($mV\ s^{-1}$)	References
$Pt_1Pd_n/(Ni,Co)(OH)_x/C$	1.0 M KOH + 1.0 M CH_3OH	0.75	190	10	This work
(110)-faceted Ni NPs	1.0 M KOH + 1.0 M CH_3OH	1.55	59.4	50	[36]
Branched Ni_3C/C	1.0 M KOH + 1.0 M CH_3OH	1.64	126.7	50	[37]
Ni-P NWs/rGO	1.0 M NaOH + 0.5 M CH_3OH	1.55	16.4	10	[38]
Ni_2Co_2	1.0 M NaOH + 0.5 M CH_3OH	1.53	38.9	50	[39]
NiCo/N-TiO_2@NaOH	1.0 M KOH + 1.0 M CH_3OH	1.50	73.8	50	[40]
$Ni_{0.75}Fe_{0.25}Se_2$	1.0 M KOH + 1.0 M CH_3OH	1.50	53.5	50	[41]
CTGU-15	0.1 M KOH + 1.0 M CH_3OH	1.52	29.8	50	[42]
$NiCuGO_{20}$	1.0 M KOH + 2.0 M CH_3OH	1.60	27.0	50	[43]
Ni-Fe_2O_3/O-CNT	1.0 M KOH + 1.0 M CH_3OH	1.50	35	50	[44]
NiP_x-R	1.0 M KOH + 0.5 M CH_3OH	1.40	400	5	[45]
NiCo/NiO-CoO	0.5 M NaOH + 0.5 M CH_3OH	1.50	75.6	50	[46]
CuO-C/$NiCo_2O_4$	1.0 M KOH + 1.0 M CH_3OH	1.60	170	50	[47]
NiO nanosheets	1.0 M KOH + 0.5 M CH_3OH	1.54	85.3	50	[48]
Co@NC-Ni_3C/G-350	1.0 M KOH + 0.5 M CH_3OH	1.47	44.8	50	[49]
3D Ni networks	1.0 M NaOH + 1.0 M CH_3OH	1.49	60.1	50	[50]

Table S6. Comparison of the noble metal-based bifunctional catalysts for methanol-assisted hydrogen evolution reaction performance.

Bifunctional catalysts	Electrolyte	Cell voltage at 10 mA cm^{-2} (V)	Current density achieved	Ref.
$Pt_1Pd_n/(Ni,Co)(OH)_x/C$	1.0 M KOH + 1.0 M CH_3OH	0.25-0.35 [a]	0.62 V @100 mA cm^{-2}<br>1.27 V@700 mA cm^{-2}	This work
NiIr-MOF/NF	1.0 M KOH + 4.0 M CH_3OH	1.39	1.53 V @100 mA cm^{-2}	[51]
$Pt-Ni_3S_2$	1.0 M KOH + 1.0 M CH_3OH	-	1.71 V @100 mA cm^{-2}	[52]
FeRu-MOF/NF	1.0 M KOH + 4.0 M CH_3OH	1.40	1.55 V @100 mA cm^{-2}	[53]
Pt-NP/NiO-NS	1.0 M KOH + 1.0 M CH_3OH	1.39	1.43 V @20 mA cm^{-2}	[54]
$Co-Rh_2$	1.0 M KOH + 1.0 M CH_3OH	1.55	2.15 V @50 mA cm^{-2}	[55]
PtPd/rGO-2	1.0 M KOH + 1.0 M CH_3OH	0.83	1.72 V @50 mA cm^{-2}	[56]
Pd@RhPd NDs	1.0 M KOH + 0.5 M CH_3OH	0.813	1.05 V @30 mA cm^{-2}	[57]
$PtNi-Se_v$	1.0 M KOH + 0.5 M CH_3OH	0.64	0.72 V @20 mA cm^{-2}	[58]
PdIn bimetallene	1.0 M KOH + 1.0 M CH_3OH	0.73	1.26 V @100 mA cm^{-2}	[59]
Pt-CoTe/NC-800	0.5 M H_2SO_4 + 1.0 M CH_3OH	0.68	0.80 V @50mA cm^{-2}	[60]
V_p-Ni_2P-Pt/CC	1.0 M KOH + 2.0 M CH_3OH	0.72	1.10 V @50mA cm^{-2}	[61]

[a] In the flow cell, due to the presence of carbon black in our catalyst itself, the contribution of the double-layer capacitance may be relatively significant at lower currents. Therefore, a rough estimate of the approximate range is provided, intended for reference purposes only.

Table S7. Comparison of energy consumption and economic cost/profit calculation to produce per kg H_2.

Indexes / Classification	Overall energy consumption (kWh/kg H_2)	Total energy efficiency	Raw materials costs (US$/ kg H_2)	Main products value (US$/ kg H_2)	Profit (US$/kg$H_2$)	Reaction condition
MOR‖HER co-driven by $Pt_1Pd_n/(Ni,Co)(OH)_x/C$	36.7	95.9%	0.7320 (Electric power costs) + 1.0927 (Methanol) + 0.0018 (Water) + 0.7090($Ca(OH)_2$) = 2.5355	6.1434 (Green H_2) + 2.2838 ($CaCO_3$) = 8.4272	5.8917	Normal pressure and temperature
(IrO_2) OER‖HER (Pt/C)	51.1	71.2%	4.3929 (Electric power costs) + US$0.0054(Water) = 4.3983	6.1434 (Green H_2) + 0.32 (O_2) = 6.4634	2.0651	Normal pressure and temperature
Methanol thermal reforming (Rh/Cu/Zr-based Catalysts)	80.8	45.0%	1.1755 (Thermal power costs) + 1.0927 (Methanol) + 0.0018 (Water) = 2.27	3.4954 (Gery H_2 -95% purity)	1.2254	High temperature (250-300 ℃)/Pressure (1-5 MPa)

Note: For simplification purposes, electrolysis was conducted at the corresponding cell voltage for the actual current density achieving and stabilizing at 50 mA cm^{-2}. For the sake of simplifying calculations and highlighting the inherent characteristics of the reaction coupling itself, the costs of devices, catalysts and electrolytes are not included in the cost accounting scope, and the methanol conversion and carbon dioxide selectivity are assumed to be 100%. Thereby, MOR‖HER cell co-driven by $Pt_1Pd_n/(Ni,Co)(OH)_x/C$ catalyst exhibits significant "Total Energy Consumption and efficiency" advantages (36.7 kWh/kg H_2, 95.9% energy efficiency) over water electrolysis of (IrO_2) OER ‖ HER (Pt/C) cell (51.1 kWh/kg H_2, 71.2% energy efficiency) and Methanol thermal reforming (80.8 kWh/kg H_2, 45.0% energy efficiency) for per kg hydrogen.

Table S8. Local Bader charge of the *H adsorption site for each catalyst.

	Pt(111)	$Pd_n/(Ni,Co)(OH)_x$	$Pt_1Pd_n/(Ni,Co)(OH)_x$
Bader charge	-0.076|e|	0.166|e|	0.202|e|

Note: Bader charge analysis is performed to understand the adsorption strength of H for different catalysts. As presented above, the more local negative charge a noble metal atom has, the stronger bond it will form with the H atom. The results shown in Supplementary Table 8 are consistent with the *H adsorption energy in Fig. 4d.

References

[1] Glenk, G.; Reichelstein, S. Economics of converting renewable power to hydrogen. *Nat. Energy.* **2019**, 4, 216-222.

[2] Nikolaidis, P.; Poullikkas, A. A comparative overview of hydrogen production processes. *Renew. Sustain. Energy Rev.* **2017**, 67, 597-611.

[3] Wang, T.; Tao, L.; Zhu, X.; Chen, C.; Chen, W.; Du, S.; Zhou, Y.; Zhou, B.; Wang, D.; Xie, C.; Long, P.; Li, W.; Wang, Y.; Chen, R.; Zou, Y.; Fu, X.; Li, Y.; Duan, X.; Wang, S. Combined anodic and cathodic hydrogen production from aldehyde oxidation and hydrogen evolution reaction. *Nat. Catal.* **2022**, 5, 66-73.

[4] Perčić, M.; Vladimir, N.; Jovanović, I. & Koričan, M. Application of fuel cells with zero-carbon fuels in short-sea shipping. *Appl. Energy*. **2022**, 309, 118463.

[5] Hohenberg, P.; Kohn, W. Inhomogeneous Electron Gas. *Phys. Rev. B*. **1964**, 136, B864-B871.

[6] Kresse, G.; Furthmüller, J. Efficient iterative schemes for ab initio total-energy calculations using a plane-wave basis set. *Phys. Rev. B*. **1996**, 54, 11169-11186.

[7] Kresse, G.; Furthmüller, J. Efficiency of ab-initio total energy calculations for metals and semiconductors using a plane-wave basis set. *Comp. Mater. Sci.* **1996**, 6, 15-50.

[8] Kresse, G.; Hafner, J. Ab initio molecular-dynamics simulation of the liquid-metal--amorphous-semiconductor transition in germanium. *Phys. Rev. B*. **1994**, 49, 14251-14269.

[9] Blöchl, P.E. Projector augmented-wave method. *Phys. Rev. B*. **1994**, 50, 17953-17979.

[10] Perdew, J.P.; Burke, K.; Ernzerhof, M. Generalized gradient approximation made simple. *Phys. Rev. Lett.* **1996**, 77, 3865-3868.

[11] Monkhorst, H.J.; Pack, J.D. Special points for Brillouin-zone integrations. *Phys. Rev. B*. **1976**, 16, 5188.

[12] Nørskov. J. K.; Rossmeisl. J, A. LogadottirL. LindqvistJ. R. KitchinT. BligaardH. Jónsson Origin of the overpotential for oxygen reduction at a fuel-cell cathode. *J. Phys. Chem. B*. **2004**, 108, 17886-17892.

[13] Chai, G.; Hou, Z.; Shu, D.; Ikeda, T.; Terakura, K. Active sites and mechanisms for oxygen reduction reaction on nitrogen-doped carbon alloy catalysts: stone-wales defect and curvature effect. *J. Am. Chem. Soc.* **2014**, 136, 13629-13640.

[14] Chai, G.; Qiu. K; Qiao.M; Titirici, M. M.; Shang, C.; Guo, Z. Active sites engineering leads to exceptional ORR and OER bifunctionality in P, N Co-doped graphene frameworks. *Energy Environ. Sci.* **2017**, 10, 1186-1195.

[15] Chai, G.; Guo, Z. Highly effective sites and selectivity of nitrogen-doped graphene/CNT catalysts for CO_2 electrochemical reduction. *Chem. Sci.* **2016**, 7, 1268-1275.

[16] Pei, A.; Xie, R.; Zhang, Y., Feng, Y., Wang, W., Zhang, S., Huang, Z., Zhu, L., Chai, G., Yang, Z. and Gao, Q. Effective electronic tuning of Pt single atoms via heterogeneous atomic coordination of $(Co,Ni)(OH)_2$ for efficient hydrogen evolution. *Energy Environ. Sci.* **2023**, 16, 1035-1048.

[17] Luo, Y.; Li, X.; Cai, X.; Zou, X.; Kang, F.; Cheng, H. M.; Liu, B. Two-dimensional MoS_2 confined $Co(OH)_2$ electrocatalysts for hydrogen evolution in alkaline electrolytes. *ACS Nano.* **2018**, 12, 4565-4573.

[18] Fang, S.; Zhu, X.; Liu, X.; Gu, J.; Liu, W.; Wang, D.; Zhang, W.; Lin, Y.; Lu, J.; Wei, S.; Li, Y. Uncovering near-free platinum single-atom dynamics during electrochemical hydrogen evolution reaction. *Nat. Commun.* **2020**, 11, 1029.

[19] Dai, J.; Zhu, Y.; Tahini, H. A.; Lin, Q.; Chen, Y.; Guan, D.; Zhou, C.; Hu, Z.; Lin, H. J.; Chan, T. S.; Chen, C.T. Single-phase perovskite oxide with super-exchange induced atomic-scale synergistic active centers enables ultrafast hydrogen evolution. *Nat. Commun.* **2020**, 11, 5657.

[20] Zhang, C.; Shi, Y.; Yu, Y.; Du, Y.; Zhang, B. Engineering sulfur defects, atomic thickness, and porous structures into cobalt sulfide nanosheets for efficient electrocatalytic alkaline hydrogen evolution. *ACS Catal.* **2018**, 8, 8077-8083.

[21] Xu, K.; Sun, Y.; Sun, Y.; Zhang, Y.; Jia, G.; Zhang, Q.; Gu, L.; Li, S.; Li, Y.; Fan, H. J. Yin-yang harmony: metal and nonmetal dual-doping boosts electrocatalytic activity for alkaline hydrogen evolution. *ACS Energy Lett.* **2018**, 3, 2750-2756.

[22] Li, Z.; Feng, Y.; Liang, Y. L.; Cheng, C. Q.; Dong, C. K.; Liu, H.; Du, X. W. Stable rhodium (IV) oxide for alkaline hydrogen evolution reaction. *Adv. Mater.* **2020**, 32, 1908521.

[23] Baek, D. S.; Jung, G. Y.; Seo, B.; Kim, J. C.; Lee, H. W.; Shin, T. J.; Jeong, H. Y.; Kwak, S. K.; Joo, S. H. Ordered mesoporous metastable α-MoC_{1-x} with enhanced water dissociation capability for boosting alkaline hydrogen evolution activity. *Adv. Funct. Mater.* **2019**, 29, 1901217.

[24] Hu, C.; Song, E.; Wang, M.; Chen, W.; Huang, F.; Feng, Z.; Liu, J.; Wang, J. Partial-single-atom, partial-nanoparticle composites enhance water dissociation for hydrogen evolution. *Adv. Sci.* **2018**, 8, 2001881.

[25] Yu, L.; Mishra, I.K.; Xie, Y.; Zhou, H.; Sun, J.; Zhou, J.; Ni, Y.; Luo, D.; Yu, F.; Yu, Y.; Chen, S. Ternary $Ni_{2(1-x)}$ $Mo_{2x}P$ nanowire arrays toward efficient and stable hydrogen evolution electrocatalysis under large-current-density. *Nano Energy.* **2018**, 53, 492-500.

[26] Zhao, D.; Sun, K.; Cheong, W.C.; Zheng, L.; Zhang, C.; Liu, S.; Cao, X.; Wu, K.; Pan, Y.; Zhuang, Z.; Hu, B. Synergistically interactive pyridinic-N-MoP sites: identified active centers for enhanced hydrogen evolution in alkaline solution. *Angew. Chem. Int. Ed.* **2020**, 132, 9067-9075.

[27] Chen, Z. J.; Cao, G. X.; Gan, L.Y.; Dai, H.; Xu, N.; Zang, M. J.; Dai, H. B.; Wu, H.; Wang, P. Highly dispersed platinum on honeycomb-like NiO@Ni film as a synergistic electrocatalyst for the hydrogen evolution reaction. *ACS Catal.* **2018**, 8, 8866-8872.

[28] Yao, N.; Li, P.; Zhou, Z.; Zhao, Y.; Cheng, G.; Chen, S.; Luo, W. Synergistically tuning water and hydrogen binding abilities over Co_4N by Cr doping for exceptional alkaline hydrogen evolution electrocatalysis. *Adv. Energy Mater.* **2019**, 9, 1902449.

[29] Shao, Q.; Wang, Y.; Yang, S.; Lu, K.; Zhang, Y.; Tang, C.; Song, J.; Feng, Y., Xiong, L., Peng, Y.; Li, Y. Stabilizing and activating metastable nickel nanocrystals for highly efficient hydrogen evolution electrocatalysis. *ACS Nano.* **2018**, 12, 11625-11631.

[30] Xiong, P.; Zhang, X.; Wan, H.; Wang, S.; Zhao, Y.; Zhang, J.; Zhou, D.; Gao, W.; Ma, R.; Sasaki, T.; Wang, G. Interface modulation of two-dimensional superlattices for efficient overall water splitting. *Nano Lett.* **2019**, 19, 4518-4526.

[31] Cao, D.; Ye, K.; Moses, O. A.; Xu, W.; Liu, D.; Song, P.; Wu, C.; Wang, C.; Ding, S.; Chen, S.; Ge, B. Engineering the in-plane structure of metallic phase molybdenum disulfide via Co and O dopants toward efficient alkaline hydrogen evolution. *ACS Nano*. **2019**, 13, 11733-11740.

[32] Yin, J; Jin, J; Zhang, H; Lu, M; Peng, Y; Huang, B; Xi, P; Yan, C. Atomic arrangement in metal-doped NiS_2 boosts the hydrogen evolution reaction in alkaline media. *Angew. Chem. Int. Ed.* **2019**, 58, 18849-18855.

[33] Liu, S.; Hu, Z.; Wu, Y.; Zhang, J.; Zhang, Y.; Cui, B.; Liu, C.; Hu, S.; Zhao, N.; Han, X.; Cao, A. Dislocation-strained IrNi alloy nanoparticles driven by thermal shock for the hydrogen evolution reaction. *Adv. Mater.*, **2020**, 32, 2006034.

[34] Zhu, Z.; Yin, H.; He, C. T.; Al-Mamun, M.; Liu, P.; Jiang, L.; Zhao, Y.; Wang, Y.; Yang, H. G.; Tang, Z.; Wang, D. Ultrathin transition metal dichalcogenide/3d metal hydroxide hybridized nanosheets to enhance hydrogen evolution activity. *Adv. Mater.* **2018**, 30, 1801171.

[35] Zhou, M.; Weng, Q., Popov, Z.I., Yang, Y., Antipina, L.Y., Sorokin, P.B., Wang, X., Bando, Y. and

Golberg, D. Construction of polarized carbon-nickel catalytic surfaces for potent, durable, and economic hydrogen evolution reactions. *ACS Nano*. **2018**, 12, 4148-4155.

[36] Li, J.; Zuo, Y.; Liu, J.; Wang, X.; Yu, X.; Du, R.; Zhang, T.; Infante-Carrió, M.F.; Tang, P.; Arbiol, J.; Llorca, J. Superior methanol electrooxidation performance of (110)-faceted nickel polyhedral nanocrystals. *J. Mater. Chem. A*. **2019**, 7, 22036-22043.

[37] Li, J.; Wei, R.; Wang, X.; Zuo, Y.; Han, X.; Arbiol, J.; Llorca, J.; Yang, Y.; Cabot, A.; Cui, C. Selective methanol-to-formate electrocatalytic conversion on branched nickel carbide. *Angew. Chem. Int. Ed.* **2020**, 132, 21012-21016.

[38] Zhang, H., Gu, C.D., Huang, M.L., Wang, X.L.; Tu, J.P. Anchoring three-dimensional network structured Ni-P nanowires on reduced graphene oxide and their enhanced electrocatalytic activity towards methanol oxidation. *Electrochem. Commun.* **2013**, 35, 108-111.

[39] Cui, X.; Guo, W.; Zhou, M.; Yang, Y.; Li, Y.; Xiao, P.; Zhang, Y.; Zhang, X. Promoting effect of Co in Ni_mCo_n (m+n= 4) bimetallic electrocatalysts for methanol oxidation reaction. *ACS Appl. Mater. Interfaces*. **2015**, 7, 493-503.

[40] Zhao, S.; Wang, T.; Ji, Z.; Song, Y.; Li, Y.; Liu, J.; Hu, W. Spatial decoupling of dehydrogenation and CO oxidation by Ni-Co-Ti hierarchical trimetallic catalyst for electrocatalytic oxidation of methanol. *Appl. Catal. B: Environ.* **2023**, 320, 122024.

[41] Li, J.; Xing, C.; Zhang, Y.; Zhang, T.; Spadaro, M. C.; Wu, Q.; Yi, Y.; He, S.; Llorca, J.; Arbiol, J.; Cabot, A. Nickel iron diselenide for highly efficient and selective electrocatalytic conversion of methanol to formate. *Small*. **2021**, 17, 2006623-2006634.

[42] Wu, Y. P.; Tian, J. W.; Liu, S.; Li, B.; Zhao, J.; Ma, L. F.; Li, D. S.; Lan, Y. Q.; Bu, X. Bi-Microporous metal–organic frameworks with cubane ($M_4(OH)_4$) (M= Ni, Co) clusters and pore-space partition for electrocata-lytic methanol oxidation reaction. *Angew. Chem., Int. Ed.* **2019**, 58, 12313-12317.

[43] Wala, M.; Szewczyk, M.; Leśniak-Ziółkowska, K.; Kazek-Kęsik, A.; Simka, W. Preparation of NiCuGO composite and investigation of its electrocatalytic properties in methanol oxidation. *Electrochim. Acta.* **2022**, 425, 140743-140755.

[44] Malik, B.; Majumder, S.; Lorenzi, R.; Perelshtein, I.; Ejgenberg, M.; Paleari, A.; Nessim, G. D. Promising Electrocatalytic Water and Methanol Oxidation Reaction Activity by Nickel Doped Hematite/Surface Oxidized Carbon Nanotubes Composite Structures. *ChemPlusChem.* **2022**, 87, e202200036-202200047.

[45] Li, S.; Ma, R.; Hu, J.; Li, Z.; Liu, L.; Wang, X.; Lu, Y.; Sterbinsky, G. E.; Liu, S.; Zheng, L.; Liu, J.; Liu, D.; Wang, J. Coordination environment tuning of nickel sites by oxyanions to optimize methanol electro-oxidation activity. *Nat. Commun.* **2022**, 13, 1-11.

[46] Rezaee, S.; Shahrokhian, S. Facile synthesis of petal-like NiCo/NiO-CoO/nanoporous carbon composite based on mixed-metallic MOFs and their application for electrocatalytic oxidation of methanol. *Appl. Catal. B: Environ.* **2019**, 244. 802-813.

[47] Sheikhi, S.; Jalali, F. Hierarchical $NiCo_2O_4$/CuO-C nanocomposite derived from copper-based metal organic framework and Ni/Co hydroxides: Excellent electrocatalytic activity towards methanol oxidation. *J. Alloys Compd.* **2022**, 7, 164510-164523.

[48] Yang, W.; Yang, X.; Jia, J.; Hou, C.; Gao, H.; Mao, Y.; Wang, C.; Li, J.; Luo, X. Oxygen vacancies confined in ultrathin nickel oxide nanosheets for enhanced electrocatalytic methanol oxidation. *Appl. Catal. B: Environ.* **2019**, 244, 1096-1102.

[49] Wang, Z.; Zhang, M.; Song, Z.; Yaseen, M.; Huang, Z.; Wang, A.; Zhu, G.; Shao, S. Synergistic catalytic enhancement of metal-organic framework derived nanoarchitectures decorated on graphene as a high-efficiency bifunctional electrocatalyst for methanol oxidation and oxygen reduction. *J. Colloid Interf. Sci.* **2022**, 624, 88-99.

[50] Guo, X.; Liang, T.; Zhang, D.; Zhang, M.; Lin, Y.; Lai, C. Facile fabrication of 3D porous nickel networks for electro-oxidation of methanol and ethanol in alkaline medium. *Mater. Chem. Phys.* **2019**, 221, 390-396.

[51] Xu, Y.; Liu, M.; Wang, M.; Ren, T.; Ren, K.; Wang, Z., Li, X.; Wang, L.; Wang, H. Methanol electroreforming coupled to green hydrogen production over bifunctional NiIr-based metal-organic framework nanosheet arrays. *Appl. Catal. B: Environ.* **2022**, 300, 120753.

[52] Zhao, Q.; Zhao, B.; Long, X.; Feng, R.; Shakouri, M.; Paterson, A.; Xiao, Q.; Zhang, Y.; Luo, J. L. Interfacial electronic modulation of dual-monodispersed Pt-Ni_3S_2 as efficacious bi-functional electrocatalysts for concurrent H_2 evolution and methanol selective oxidation. *Nano-Micro Lett.* **2024**, 16, 1-17.

[53] Ling, Q.; Zhao, Z.; Li, Z.; Yan, K.; Ding, C.; Chen, P.; Sun, Z.; He, G.; Zhang, M. FeRu-based metal-organic framework bifunctional catalysts for hydrogen production and methanol electroreforming. *J. Mater. Chem. A.* **2023**, 11, 2876-2888.

[54] Ma, G.; Zhang, X.; Zhou, G.; Wang, X. Hydrogen production from methanol reforming electrolysis at NiO nanosheets supported Pt nanoparticles. *Chem. Eng. J.* **2021**, 411, 128292.

[55] Guo, Y.; Yang, X.; Liu, X.; Tong, X.; Yang, N. Coupling methanol oxidation with hydrogen evolution on bifunctional Co-doped Rh electrocatalyst for efficient hydrogen generation. *Adv. Funct. Mater.* **2023**, 33, 2209134.

[56] Feng, Y.; Zhu, L.; Pei, A.; Zhang, S.; Liu, K.; Wu, F.; Li, W. Platinum-palladium-on-reduced graphene oxide as bifunctional electrocatalysts for highly active and stable hydrogen evolution and methanol oxidation reaction. *Nanoscale*. **2023**, 15, 16904-16913.

[57] Jiang, Y. C.; Sun, H. Y.; Li, Y. N.; He, J. W.; Xue, Q.; Tian, X.; Li, F.; Yin,S.; Li, D.; Chen, Y. Bifunctional Pd@RhPd core-shell nanodendrites for methanol electrolysis. *ACS Appl. Mater. Interfaces*. **2021**, 13, 35767-35776.

[58] Jin, Y.; Zhang, Z., Yang, H., Wang, P., Shen, C., Cheng, T.; Huang, X.; Shao, Q. Boosting hydrogen production with ultralow working voltage by selenium vacancy-enhanced ultrafine platinum-nickel nanowires. *SmartMat*. **2022**, 3, 130-141.

[59] Yin, S.; Liu, S.; Wang, Z.; Xu, Y.; Li, X.; Wang, H.; Wang, L. Methanol-assisted energy-saving hydrogen production over defect-rich perforated PdIn bimetallene. *Chem. Eng. J.* **2022**, 435, 134711.

[60] Zhou, Y.; Kuang, Y.; Hu, G.; Wang, X.; Feng, L. An effective Pt-CoTe/NC catalyst of bifunctional methanol electrolysis for hydrogen generation. *Mater. Today Phys.* **2022**, 27, 100831.

[61] Wu, X.; Zhang, Y.; Yang, Y.; Fu, G.; Si, F.; Chen, J.; Ahmad, M.; Zhang, Z.; Ye, C.; Zhang, J.; Fu, X.; Luo, J. L. Ni_2P with phosphorus vacancy supported Pt clusters for efficiently electrocatalytic co-production of hydrogen and value-added chemicals from methanol-water at low potential. *Chem. Eng. J.* **2023**, 452, 139057.
